# Supplementary material for: Sitravatinib in combination with nivolumab plus ipilimumab in patients with advanced clear cell renal cell carcinoma: a phase 1 trial
Source: Nat Commun. 2025 Jan 10;16:578. doi: 10.1038/s41467-024-55642-8 (PMC11724043; doi:10.1038/s41467-024-55642-8)
Supplement: Supplementary file 1 — Supplementary Information [file 41467_2024_55642_MOESM1_ESM.pdf]

# Supplementary Information

**Sitravatinib in combination with nivolumab plus ipilimumab in patients with advanced clear cell renal cell carcinoma: a phase 1 trial**


Pavlos Msaouel,[1,2,3,*] Kai Yu,[4] Ying Yuan,[5] Jianfeng Chen,[1,3] Xinmiao Yan,[4] Menuka Karki,[1,3] Fei Duan,[1,3] Rahul A. Sheth,[6] Priya Rao,[7] Kanishka Sircar,[2,7] Amishi Y. Shah,[1] Amado J. Zurita,[1] Giannicola Genovese,[1,3,4] Min Li,[1,3] Chih-Chen Yeh,[1,3] Minghao Dang,[4] Guangchun Han,[4] Yanshuo Chu,[4] Max Hallin,[8] Peter Olson,[8] Rui Yang,[8] Daniela Slavin,[8] Hirak Der-Torossian,[8] Curtis D. Chin,[8] Nizar M. Tannir,[1] Linghua Wang[4,9,*] Jianjun Gao,[1,3,*]

[1]Department of Genitourinary Medical Oncology, The University of Texas MD Anderson Cancer Center, Houston, TX 77030, USA.
[2]Department of Translational Molecular Pathology, The University of Texas MD Anderson Cancer Center, Houston, TX 77030, USA.
[3]David H. Koch Center for Applied Research of Genitourinary Cancers, The University of Texas, MD Anderson Cancer Center, Houston, TX 77030, USA.
[4]Department of Genomic Medicine, The University of Texas MD Anderson Cancer Center, Houston, USA.
[5]Department of Biostatistics, The University of Texas MD Anderson Cancer Center, Houston, TX 77030, USA.
[6]Department of Interventional Radiology, The University of Texas MD Anderson Cancer Center, Houston, TX 77030, USA.
[7]Department of Pathology, The University of Texas MD Anderson Cancer Center, Houston, TX 77030, USA.
[8]Mirati Therapeutics, Inc., San Diego, CA, 92121, USA
[9]The University of Texas MD Anderson Cancer Center UT Health Houston Graduate School of Biomedical Sciences (GSBS), Houston, TX 77030, USA.

Corresponding Authors:
Dr. Pavlos Msaouel, M.D., Ph.D.
Department of Genitourinary Medical Oncology, Unit 1374
The University of Texas MD Anderson Cancer Center
1155 Pressler St
Houston, TX 77030-3721
Fax: (713) 745-7575
E-mail: PMsaouel@mdanderson.org

Dr. Jianjun Gao, M.D., Ph.D.
Department of Genitourinary Medical Oncology, Unit 1374
The University of Texas MD Anderson Cancer Center
1155 Pressler St
Houston, TX 77030-3721
Fax: (713) 745-1625
E-mail: jgao1@mdanderson.org

Dr. Linghua Wang, M.D., Ph.D.
Department of Genomic Medicine, Unit 1954
The University of Texas MD Anderson Cancer Center,
1515 Holcombe Blvd
Houston, TX 77030-4009
E-mail: lwang22@mdanderson.org

## Table of Contents

**Supplementary Tables:**
Supplementary Table S1. DLTs and other notable AEs in all four cohorts. Page 3
Supplementary Table S2. Dose interruptions, reductions or discontinuations due to treatment-related adverse events. Page 4
Supplementary Table S3. Duration of steroid use in patients who developed immune-related adverse events. Page 5
Supplementary Table S4. Clinical efficacy by dose cohort. Page 6
Supplementary Table S5. Patient samples used for single-cell RNA sequencing. Page 7
Supplementary Table S6. Multivariable model of disease-specific survival in the TCGA KIRC dataset. Page 8
Supplementary Table S7. Multivariable model of overall survival in the TCGA KIRC dataset. Page 8

**Supplementary Figures:**
Supplementary Figure S1. Sitravatinib plasma concentrations for each treatment cohort at predose, 30 minutes and 7 hours after triplet therapy cycle. Page 9
Supplementary Figure S2. Single-cell landscape of tumor microenvironment and epithelial cells from longitudinally collected tumor samples at baseline and during trial therapy Page 10
Supplementary Figure S3. Characterization of malignant cell clusters. Page 12
Supplementary Figure S4. Longitudinal CD4+ T cell changes during trial therapy. Page 14
Supplementary Figure S5. Longitudinal CD8+ T cell changes during trial therapy. Page 15
Supplementary Figure S6. Longitudinal myeloid cell changes during trial therapy. Page 16
Supplementary Figure S7. Longitudinal single-cell transcriptomic changes during trial therapy in paired baseline and EOT samples from the same patients. Page 18
Supplementary Figure S8. Longitudinal single-cell transcriptomic changes during trial therapy in tumor samples from the 8 patients enrolled in cohort 4. Page 20
Supplementary Figure S9. Single-cell transcriptomic differences in baseline clear cell renal cell carcinoma with sarcomatoid and/or rhabdoid dedifferentiation compared with baseline clear cell renal cell carcinoma without Sarcomatoid or rhabdoid dedifferentiation Page 22

**Clinical Trial Protocol** Page 24

**Supplementary Table S1.** DLTs and other notable AEs in all four cohorts.

| Patient ID | Cohort | DLT | DLT type | Other notable AEs |
|---|---|---|---|---|
| 001 | 1 | No | N/A | None |
| 002 | 1 | No | N/A | Grade 3 immune-related colitis (biopsy confirmed) |
| 003 | 1 | No | N/A | Grade 2 immune-related myocarditis (biopsy confirmed) |
| 004 | 1 | Yes | Grade 3 immune-related myositis / myasthenia gravis (biopsy confirmed) | None |
| 005 | 1 | No | N/A | Grade 3 immune-related myalgias/arthralgias; fatal acute hepatitis possibly related to trial therapy |
| 007 | 1 | No | N/A | Grade 3 immune-related colitis (biopsy confirmed) |
| 009 | 1 | No | N/A | Grade 3 immune-related pneumonitis; grade 1 immune-related nephritis |
| 011 | 2 | No | N/A | None |
| 012 | 2 | No | N/A | None |
| 013 | 2 | No | N/A | None |
| 014 | 3 | No | N/A | None |
| 015 | 3 | No | N/A | None |
| 016 | 3 | No | N/A | None |
| 018 | 4 | No | N/A | None |
| 019 | 4 | Yes | Grade 3 immune-related myositis / myasthenia gravis (biopsy confirmed) | None |
| 020 | 4 | No | N/A | None |
| 021 | 4 | No | N/A | None |
| 024 | 4 | No | N/A | None |
| 026 | 4 | Yes | Grade 3 hypertension due to sitravatinib (not immune-related) | Grade 3 immune-related pneumonitis |
| 027 | 4 | No | N/A | None |
| 028 | 4 | No | N/A | None |
| 029 | 4 | Yes | Persistent grade 2 transaminase elevation possibly related to trial therapy | None |

AE, adverse event; DLT, dose-limiting toxicity; N/A, not applicable.

**Supplementary Table S2.** Dose interruptions, reductions or discontinuations due to treatment-related adverse events.

| Adverse event category [n (%)] | Cohort 1 (N=7) | Cohort 2 (N=3) | Cohort 3 (N=3) | Cohort 4 (N=9) | Total (N=22) |
|---|---|---|---|---|---|
| Treatment-related adverse events leading to study drug dose reduction or interruption | 6 (85.7) | 0 | 3 (100) | 8 (88.9) | 17 (77.3) |
| Dose reduction or interruption of sitravatinib | 6 (85.7) | 0 | 3 (100) | 8 (88.9) | 17 (77.3) |
| Dose interruption of nivolumab | 1 (14.3) | 0 | 0 | 3 (33.3) | 4 (18.2) |
| Dose interruption of ipilimumab | 1 (14.3) | 0 | 0 | 1 (11.1) | 2 (9.1) |
| Treatment-related adverse events leading to study drug discontinuation | 6 (85.7) | 0 | 0 | 4 (44.4) | 10 (45.5) |
| Discontinuation of sitravatinib | 0 | 0 | 0 | 1 (11.1) | 1 (4.5) |
| Discontinuation of nivolumab | 6 (85.7) | 0 | 0 | 3 (33.3) | 9 (40.9) |
| Discontinuation of ipilimumab | 4 (57.1) | 0 | 0 | 3 (33.3) | 7 (31.8) |
| Discontinuation of all study drugs | 0 | 0 | 0 | 0 | 0 |

**Supplementary Table S3.** Duration of steroid use in patients who developed immune-related adverse events.

| Patient ID | Cohort | Duration of steroid use (days) |
|---|---|---|
| 001 | 1 | 0 |
| 002 | 1 | 132 |
| 003 | 1 | 124 |
| 004 | 1 | 11 |
| 005 | 1 | 94 |
| 007 | 1 | 28 |
| 009 | 1 | 77 |
| 011 | 2 | 0 |
| 012 | 2 | 0 |
| 013 | 2 | 0 |
| 014 | 3 | 0 |
| 015 | 3 | 5 |
| 016 | 3 | 0 |
| 018 | 4 | 0 |
| 019 | 4 | 34 |
| 020 | 4 | 0 |
| 021 | 4 | 59 |
| 024 | 4 | 0 |
| 026 | 4 | 90 |
| 027 | 4 | 0 |
| 028 | 4 | 0 |
| 029 | 4 | 0 |

**Supplementary Table S4.** Clinical efficacy by dose cohort.

| Cohort | Endpoint | Result | p-value (versus cohort 4) |
|---|---|---|---|
| 1 | ORR | 5/7 (71.4%) | 0.32* |
| 2 | ORR | 0/3 (0%) | 0.51* |
| 3 | ORR | 1/3 (33%) | 1* |
| 4 | ORR | 3/9 (33%) | - |
| | | | |
| 1 | 1-year PFS probability | 66.7% | 0.25[#] |
| 2 | 1-year PFS probability | 50% | 0.85[#] |
| 3 | 1-year PFS probability | 66.7% | 0.55[#] |
| 4 | 1-year PFS probability | NE | - |
| | | | |
| 1 | 1-year OS probability | 85.7% | 0.51[#] |
| 2 | 1-year OS probability | 66.7% | 0.13[#] |
| 3 | 1-year OS probability | 66.7% | 0.38[#] |
| 4 | 1-year OS probability | 87.5% | - |

NE, not estimable; ORR, objective response rate; OS, overall survival; PFS, progression-free survival; *Two-sided Fisher's exact test; [#]Logrank test

**Supplementary Table S5.** Patient samples used for single-cell RNA sequencing.

| Patient ID | Timepoint | Dose cohort | Biopsy site | Biopsy organ | Presence of sarcomatoid / rhabdoid dedifferentiation | Best tumor change from baseline | Best objective response |
|---|---|---|---|---|---|---|---|
| 012 | Baseline (Pre_NR) | 2 | Retroperitoneal lymph node | Lymph node | Rhabdoid | -25% | SD |
| 013 | Baseline (Pre_NR) | 2 | Mediastinal lymph node | Lymph node | No | -31.4% | SD |
| 014 | Baseline (Pre_R) | 3 | Left renal mass | Kidney | No | -40% | PR |
| 016 | Baseline (Pre_NR) | 3 | Liver nodule | Liver | No | -28.8% | SD |
| 016 | C2 (Post) | 3 | Liver nodule | Liver | No | -28.8% | SD |
| 016 | EOT | 3 | Liver nodule | Liver | No | -28.8% | SD |
| 018 | Baseline (Pre_NR) | 4 | Lung nodule | Lung | Sarcomatoid and rhabdoid | -26.8% | SD |
| 018 | C2 (Post) | 4 | Lung nodule | Lung | Sarcomatoid and rhabdoid | -26.8% | SD |
| 018 | EOT | 4 | Lung nodule | Lung | Sarcomatoid and rhabdoid | -26.8% | SD |
| 019 | Baseline (Pre_NR) | 4 | Peritoneal mass | Peritoneum | No | -26.3% | SD |
| 020 | Baseline (Pre_R) | 4 | Retroperitoneal lymph node | Lymph node | No | -53.3% | PR |
| 020 | EOT | 4 | Right hip nodule | Subcutaneous tissue | No | -53.3% | PR |
| 021 | Baseline (Pre_NR) | 4 | Retroperitoneal lymph node | Lymph node | No | -50% | SD |
| 024 | Baseline (Pre_NR) | 4 | Lung nodule | Lung | No | -26.8% | SD |
| 024 | C2 (Post) | 4 | Lung nodule | Lung | No | -26.8% | SD |
| 026 | Baseline (Pre_NR) | 4 | Retroperitoneal lymph node | Lymph node | No | -16.9% | SD |
| 027 | Baseline (Pre_R) | 4 | Lung nodule | Lung | Rhabdoid | -60% | PR |
| 027 | C2 (Post) | 4 | Lung nodule | Lung | Rhabdoid | -60% | PR |
| 029 | Baseline (Pre_NR) | 4 | Right renal mass | Kidney | No | -25% | PD |

C2 (Post): prior to the second infusion of nivolumab plus ipilimumab; EOT, end of treatment; PD, progressive disease; PR, partial response; Pre_NR, baseline samples from patients that did not demonstrate objective response to trial therapy; Pre_R, baseline samples from patients that demonstrated objective response to trial therapy; SD, stable disease.

**Supplementary Table S6. Multivariable model of disease-specific survival in the TCGA KIRC dataset.** The p-value was calculated using the two-sided Cox regression score test to compare survival distributions between the defined groups without adjustment for multiple comparisons.

| Variable | Hazard ratio | 95% CI | p-value |
|---|---|---|---|
| C8 signature high (Reference "C8 signature low") | 1.67 | 1.56 – 2.43 | 0.0064 |
| Stage (Reference: "all other stages") | | | |
| Stage I | 0.03 | 0.004 – 0.27 | 0.0014 |
| Stage II | 0.16 | 0.019 – 1.24 | 0.079 |
| Stage III | 0.26 | 0.035 – 1.91 | 0.18 |
| Stage IV | 1.06 | 0.15 – 7.73 | 0.96 |
| History of neoadjuvant treatment: Yes (Reference: "No") | 1.33 | 0.64 – 2.76 | 0.45 |

**Supplementary Table S7. Multivariable model of overall survival in the TCGA KIRC dataset.** The p-value was calculated using the two-sided Cox regression score test to compare survival distributions between the defined groups without adjustment for multiple comparisons.

| Variable | Hazard ratio | 95% CI | p-value |
|---|---|---|---|
| C8 signature high (Reference "C8 signature low") | 1.53 | 1.14 – 2.07 | 0.0052 |
| Stage (Reference: "all other stages") | | | |
| Stage I | 0.16 | 0.02 – 1.16 | 0.07 |
| Stage II | 0.21 | 0.03 – 1.59 | 0.13 |
| Stage III | 0.39 | 0.05 – 2.83 | 0.35 |
| Stage IV | 1.05 | 0.15 – 7.67 | 0.96 |
| History of neoadjuvant treatment: Yes (Reference: "No") | 1.65 | 0.88 – 3.08 | 0.12 |

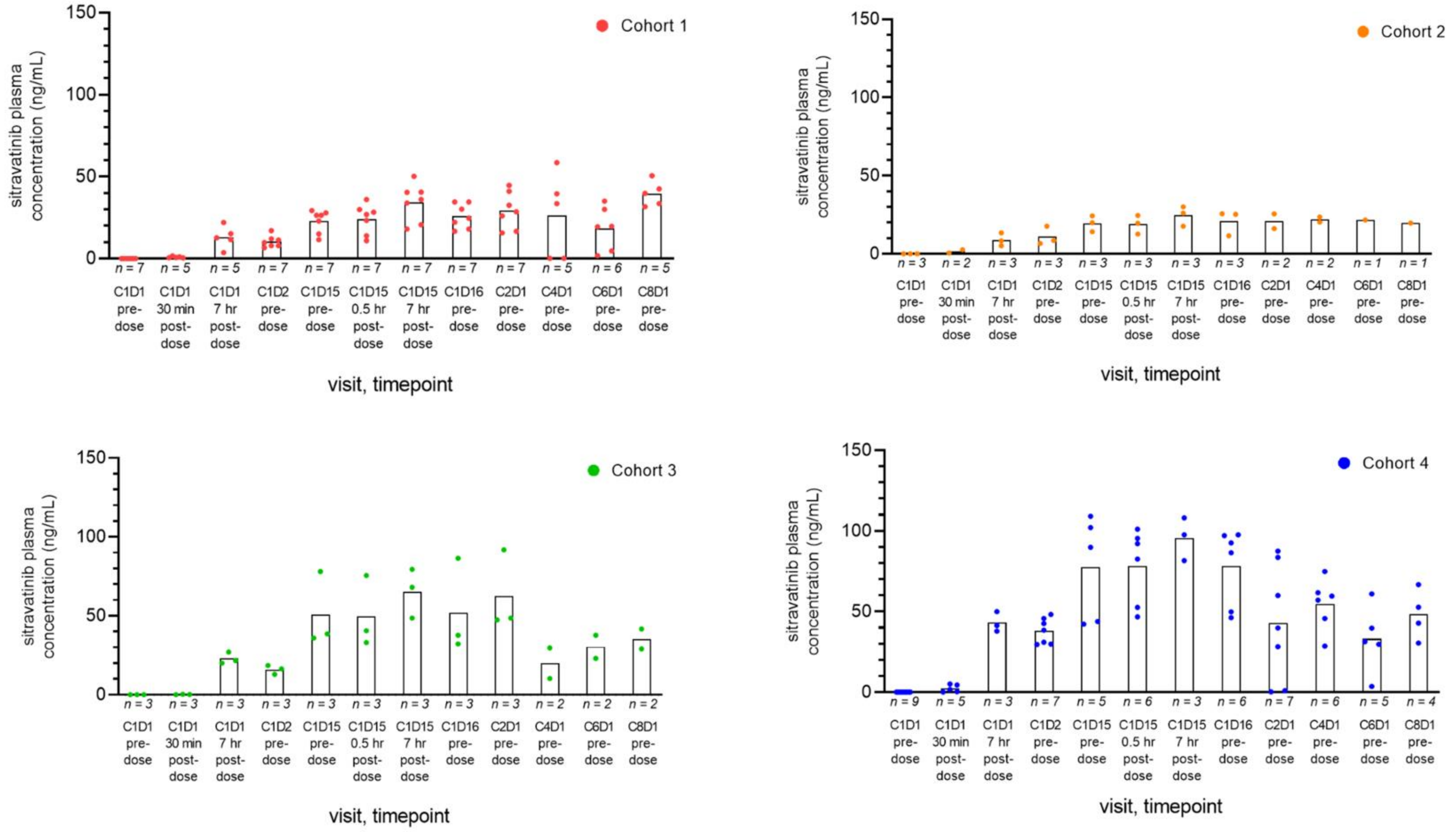


**Supplementary Figure S1.** Sitravatinib plasma concentrations for each treatment cohort at predose, 30 minutes and 7 hours after triplet therapy cycle. Source data are provided in the Source Data file.

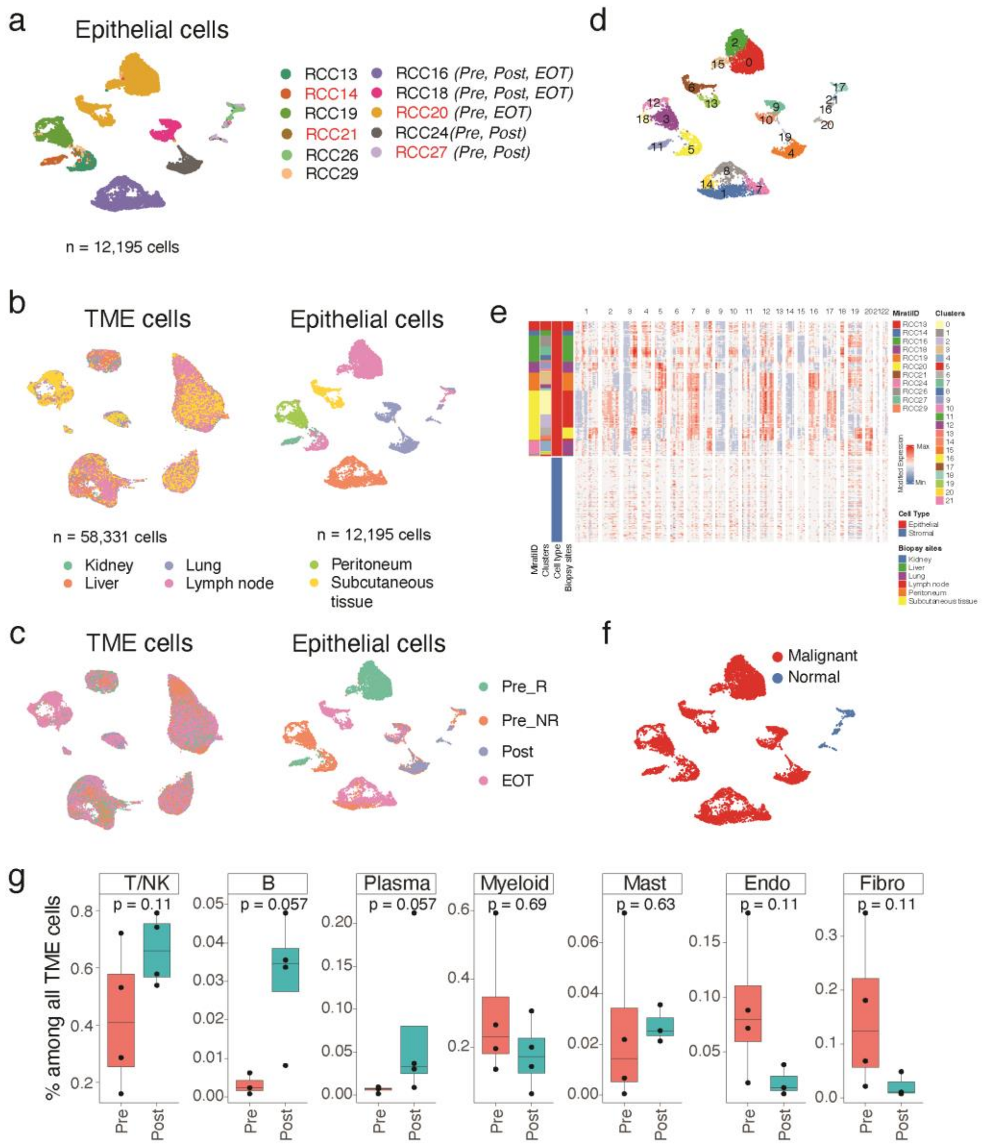


**Supplementary Figure S2.** Single-cell landscape of tumor microenvironment (TME) and epithelial cells from longitudinally collected tumor samples at baseline and during trial therapy. **(a)** Uniform manifold approximation and projection (UMAP) scRNA-seq data from epithelial cells. Colors and numbers indicate different patients. Patients marked in red are PR patients. **(b)** UMAP of scRNA-seq data from TME cells and epithelial cells. Colors indicate biopsy sites. **(c)** UMAP of scRNA-seq data from TME cells and epithelial cells. Colors indicate collection point and treatment response. Pre_R (n=3; baseline samples from patients that demonstrated an objective response to trial therapy), Pre_NR (n=9; baseline samples from patients that did not demonstrate an objective response to trial therapy), Post (n=4; samples collected at the C2 timepoint), and EOT (n=3; samples collected at the EOT timepoint). (**d**) UMAP of scRNA-seq data from epithelial cells. Colors indicate scRNA-seq clusters. (**e**)

Inferred copy number variance (CNV) on epithelial cells to identify malignant cells. (**f**) UMAP of scRNA-seq data from epithelial cells. Colors indicate cell types. (g) Differences in TME cells between different response groups: Pre_R (n=3; baseline samples from patients that demonstrated an objective response to trial therapy), Pre_NR (n=9; baseline samples from patients that did not demonstrate an objective response to trial therapy). Box-and-whisker plots show all values with range (whiskers), interquartile range (box) and median (center line). Source data are provided in the Source Data file.

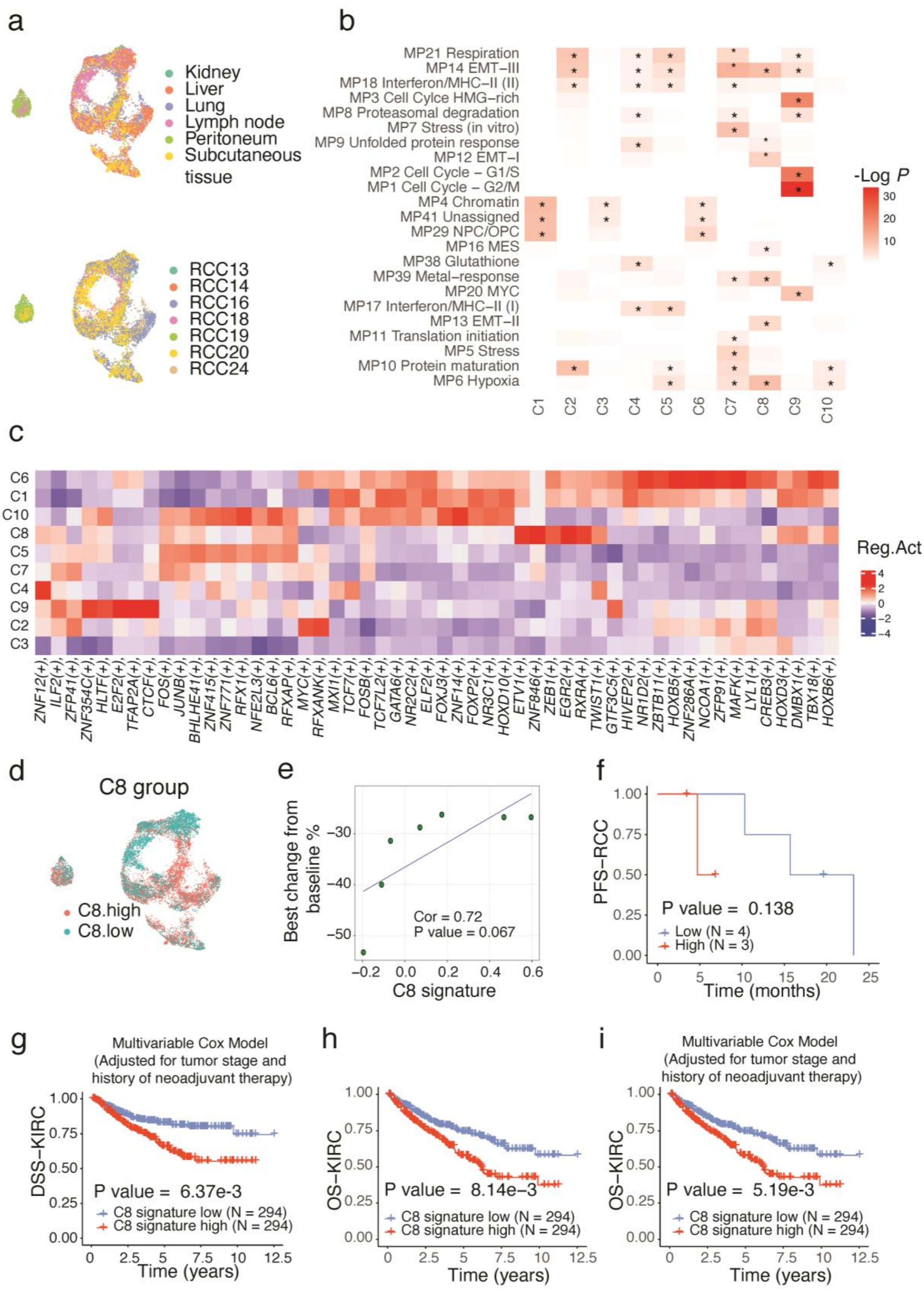


**Supplementary Figure S3.** Characterization of malignant cell clusters. (**a**) Uniform manifold approximation and projection (UMAP) scRNA-seq data from epithelial cells. Colors indicate different biopsy sites and patients. (**b**) Meta-program enrichment analysis for signature genes in different cell

clusters. (**c**) Transcription factor activity analysis reveals differential activity of transcription factors between different clusters. (**d**) UMAP of C8 signature expression groups in the scRNA-seq dataset. (e) Correlation of tumor response (maximum change in the sum of the longest dimensions from baseline) with C8 signature in baseline (pre-treatment) samples. (**f**) Progression-free survival based on C8 signature expression level in baseline (pre-treatment) samples. **(g)** Disease-specific survival (DSS) differences based on C8 signature expression level in patients with clear cell renal cell carcinoma (KIRC) in TCGA dataset following adjustment for tumor stage and history of neoadjuvant therapy. **(h)** Unadjusted overall survival (OS) differences based on C8 signature expression level in patients with KIRC in TCGA dataset. **(i)** OS differences based on C8 signature expression level in patients with KIRC in TCGA dataset following adjustment for tumor stage and history of neoadjuvant therapy. Source data are provided in the Source Data file.

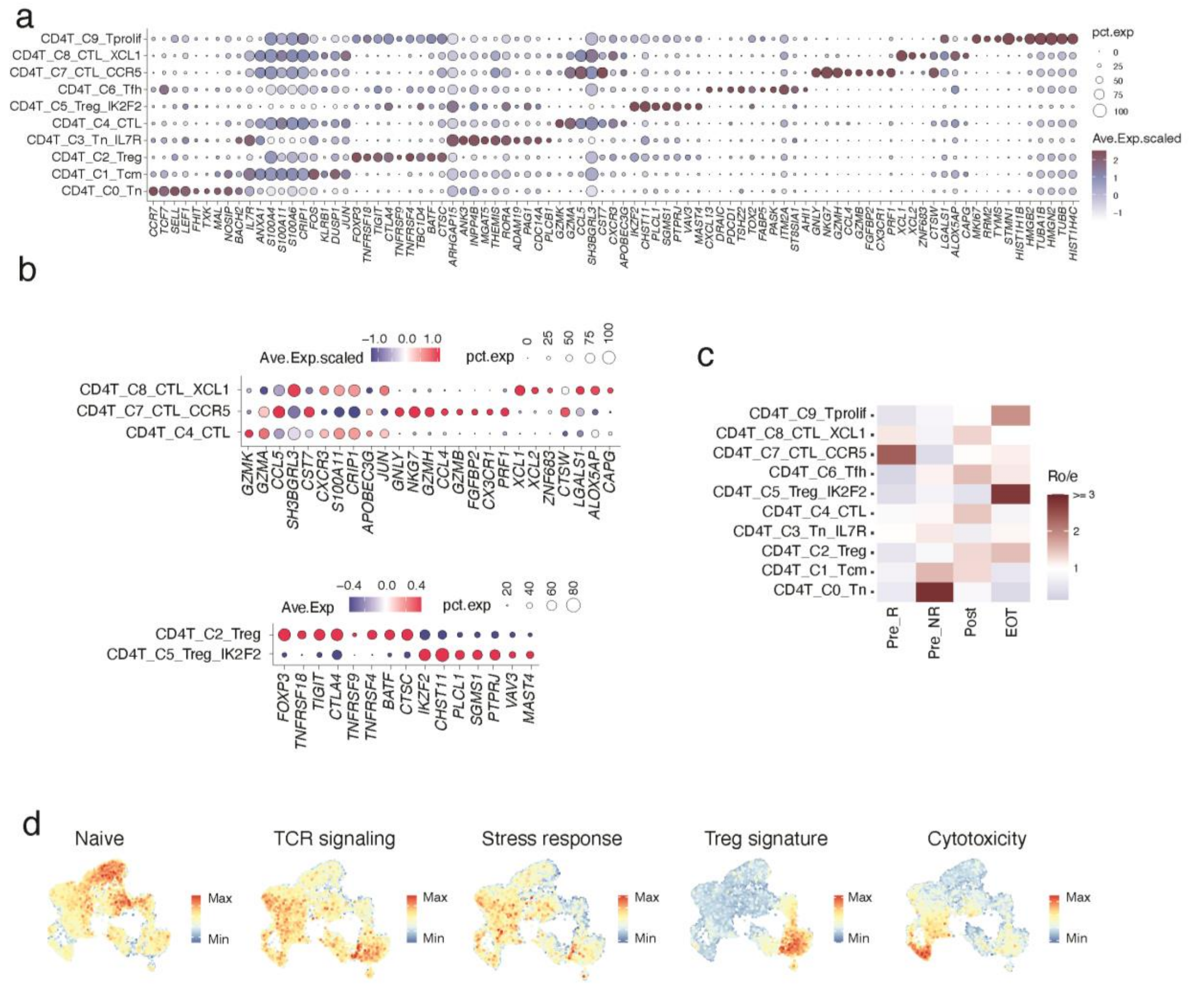


**Supplementary Figure S4.** Longitudinal CD4+ T cell changes during trial therapy. (**a**) Expression levels and frequencies of top 10 significant markers across CD4+ T cell clusters. (**b**) Bubble plot showing key marker gene expression between the three CD4+ CTL T cell clusters and two Treg T cell clusters. (**c**) Distribution of CD4+ T cell states across tissue groups in all TME cells. Heat map showing tissue prevalence estimated by Ro/e. Pre_R (n=3; baseline samples from patients that demonstrated an objective response to trial therapy), Pre_NR (n=9; baseline samples from patients that did not demonstrate an objective response to trial therapy), Post (n=4; samples collected at the C2 timepoint), and EOT (n=3; samples collected at the EOT timepoint). (**d**) Expression levels of selected pathway signatures across CD4+ T cell clusters. Source data are provided in the Source Data file.

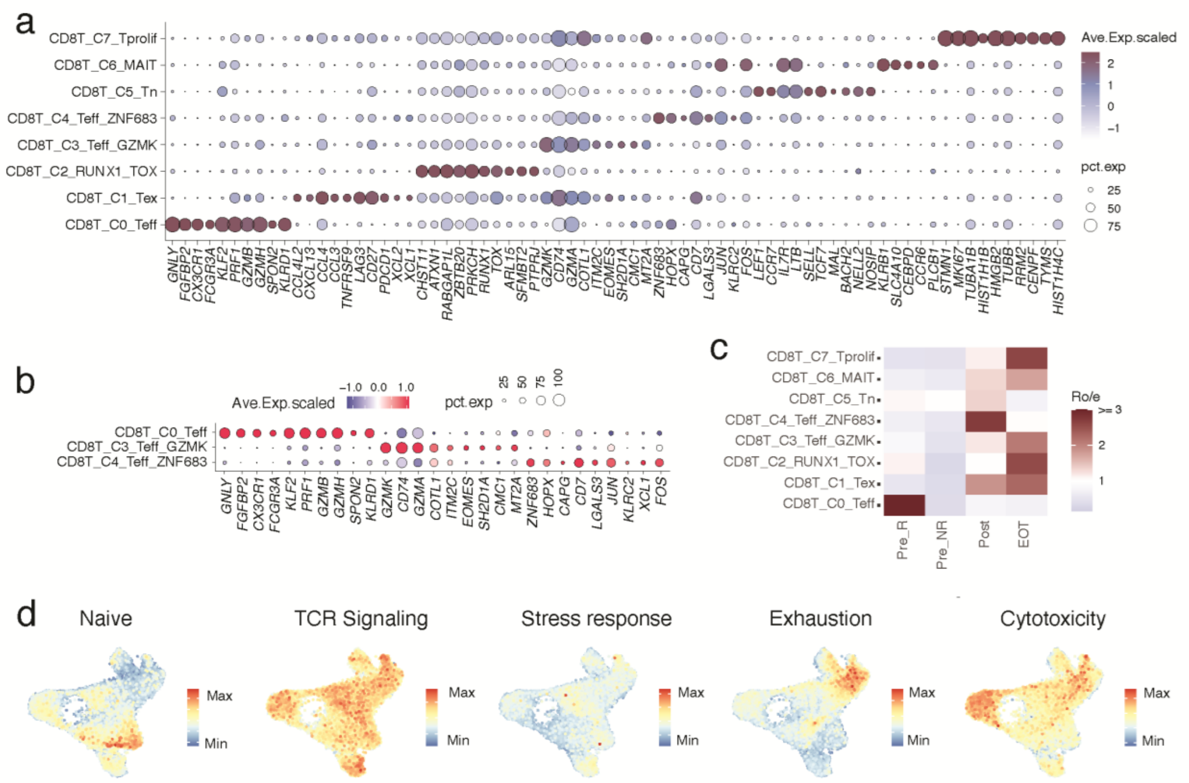


**Supplementary Figure S5.** Longitudinal CD8+ T cell changes during trial therapy. (**a**) Expression levels and frequencies of top 10 significant markers across CD8+ T cell clusters. (**b**) Bubble plot showing key marker gene expression between the three CD8+ Teff T cell clusters. (**c**) Distribution of CD8+ T cell states across tissue groups in all TME cells. Heat map showing tissue prevalence estimated by Ro/e. Pre_R (n=3; baseline samples from patients that demonstrated an objective response to trial therapy), Pre_NR (n=9; baseline samples from patients that did not demonstrate an objective response to trial therapy), Post (n=4; samples collected at the C2 timepoint), and EOT (n=3; samples collected at the EOT timepoint). (**d**) Expression levels of selected pathway signatures across CD8+ T cell clusters. Source data are provided in the Source Data file.

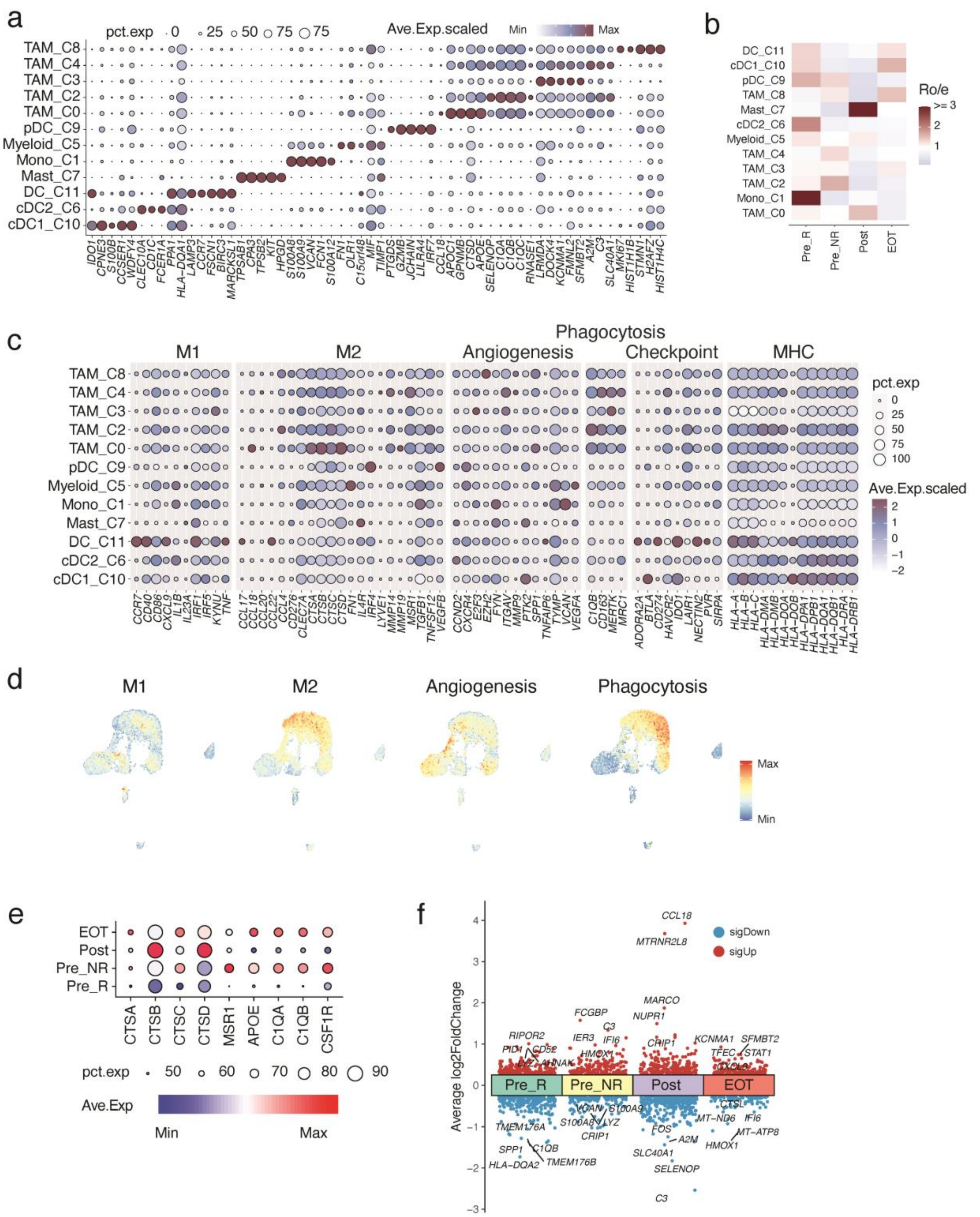


**Supplementary Figure S6.** Longitudinal myeloid cell changes during trial therapy. (**a**) Expression levels and frequencies of top 10 significant markers across myeloid cell clusters. (**b**) Distribution of myeloid cell clusters across tissue groups in all TME cells. Heat map showing tissue prevalence estimated by the ratio of observed to expected cell number (Ro/e). Pre_R (n=3; baseline samples from patients that demonstrated an objective response to trial therapy), Pre_NR (n=9; baseline samples from patients that did not demonstrate an objective response to trial therapy), Post (n=4; samples collected at

the C2 timepoint), and EOT (n=3; samples collected at the EOT timepoint). (**c**) Expression levels and frequencies of selected markers across myeloid cell clusters. (**d**) Expression levels of pathway signatures across myeloid cell clusters. (**e**) Selected macrophage M2 polarization signature gene activities across different groups in macrophage and monocytes cell clusters. (**f**) Differentially expressed genes across different groups in macrophage and monocytes cell clusters. Significant up and down regulation are determined by log2FoldChange > 0.25 and adjusted P value < 0.05. Source data are provided in the Source Data file.

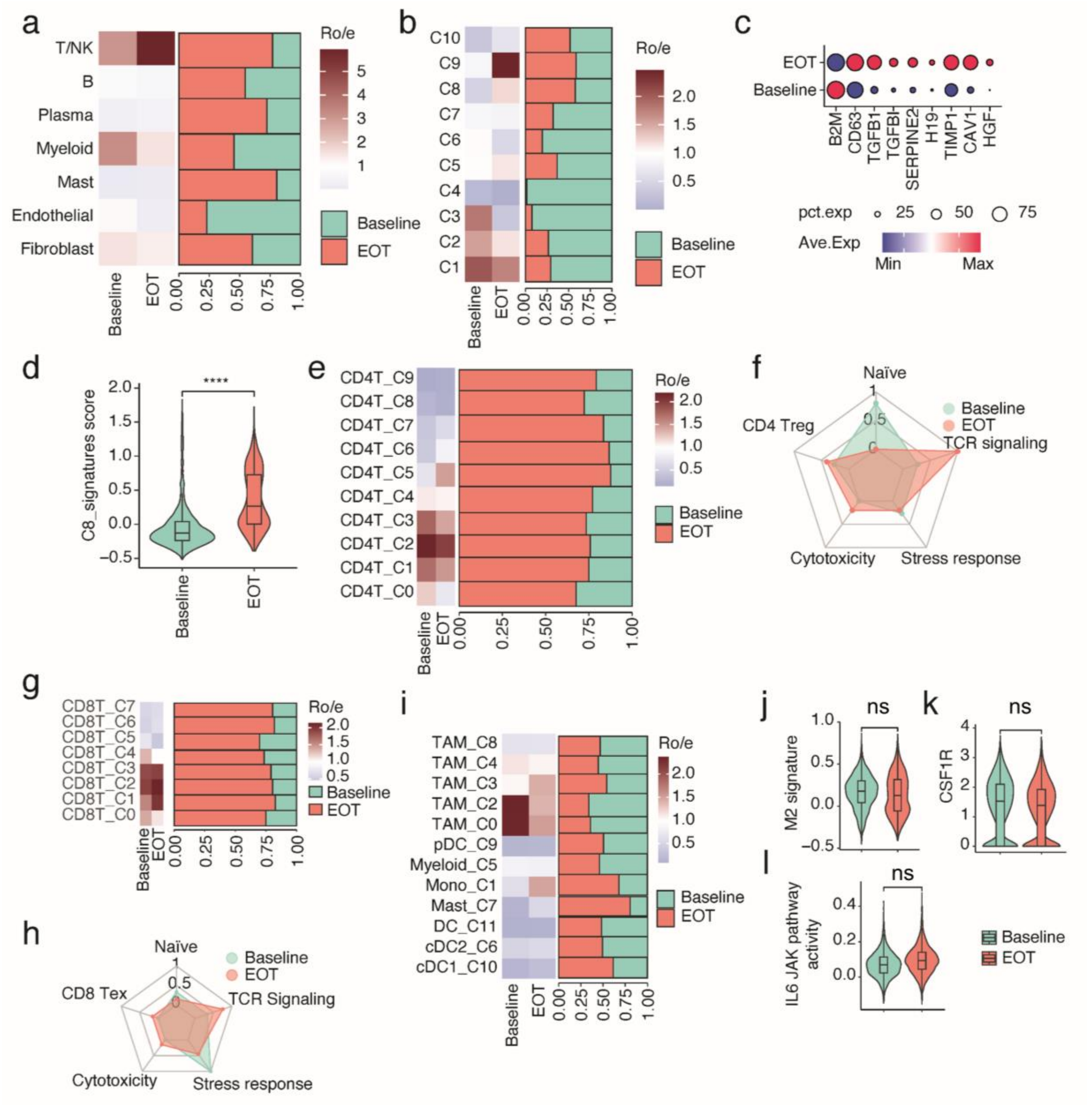


**Supplementary Figure S7.** Longitudinal single-cell transcriptomic changes during trial therapy in paired baseline and end of treatment (EOT) samples from the same patients (n =3). The samples used were from patients ID #016, 018, and 020 listed in Supplementary Table S13. (**a**) Changes in distribution of tumor microenvironment (TME) cell clusters between baseline and EOT timepoints. The heat map on the left shows tissue prevalence estimated by the ratio of observed to expected cell number (Ro/e). The bar plot on the right shows the relative proportion of cells from all patients for each TME cell subset. (**b**) Changes in the distribution of malignant cell clusters between baseline and EOT. The heat map on the left shows tissue prevalence estimated by the ratio of observed to expected cell number (Ro/e). The bar plot on the right shows the relative proportion of cells from all patients for each

malignant cell subset. (**c**) Bubble plot showing differences in expression of key marker genes from the C2 and C8 cell clusters between baseline and EOT timepoints. (**d**) Expression levels of C8 signature in malignant cells at baseline and EOT. N = 5326 cells for the baseline and 2597 cells for the EOT timepoint. (**e**) Distribution of CD4+ T cell clusters at baseline and EOT. The heat map on the left shows tissue prevalence estimated by the ratio of observed to expected cell number (Ro/e). The bar plot on the right shows the relative proportion of cells from all patients for each CD4+ T cell subset. (**f**) Radar plot showing enrichment of selected five CD4+ T cell states at baseline and EOT. (**g**) Distribution of CD8+ T cell clusters at baseline and EOT. The heat map on the left shows tissue prevalence estimated by the ratio of observed to expected cell number (Ro/e). The bar plot on the right shows the relative proportion of cells from all patients for each CD8+ T cell subset. (**h**) Radar plot showing enrichment of selected five CD8+ T cell states at baseline and EOT. (**i**) Distribution of myeloid cell clusters at baseline and EOT. The heat map on the left shows tissue prevalence estimated by the ratio of observed to expected cell number (Ro/e). The bar plot on the right shows the relative proportion of cells from all patients for each myeloid cell subset. (**j**) Expression levels of M2 macrophage related signature in macrophage and monocytes cell clusters between baseline and EOT tissues. N = 3093 cells for the baseline and 2677 cells for the EOT timepoint. (**k**) Expression levels of CSF1R gene in macrophage and monocytes cell clusters between baseline and EOT tissues. N = 3093 cells for the baseline and 2677 cells for the EOT timepoint. (**l**) Expression levels of IL6-JAK pathway genes in macrophage and monocytes cell clusters between baseline and EOT tissues. N = 3093 cells for the baseline and 2677 cells for the EOT timepoint. **** : p-value < 0.0001 ; ns: not significant. Source data are provided in the Source Data file.

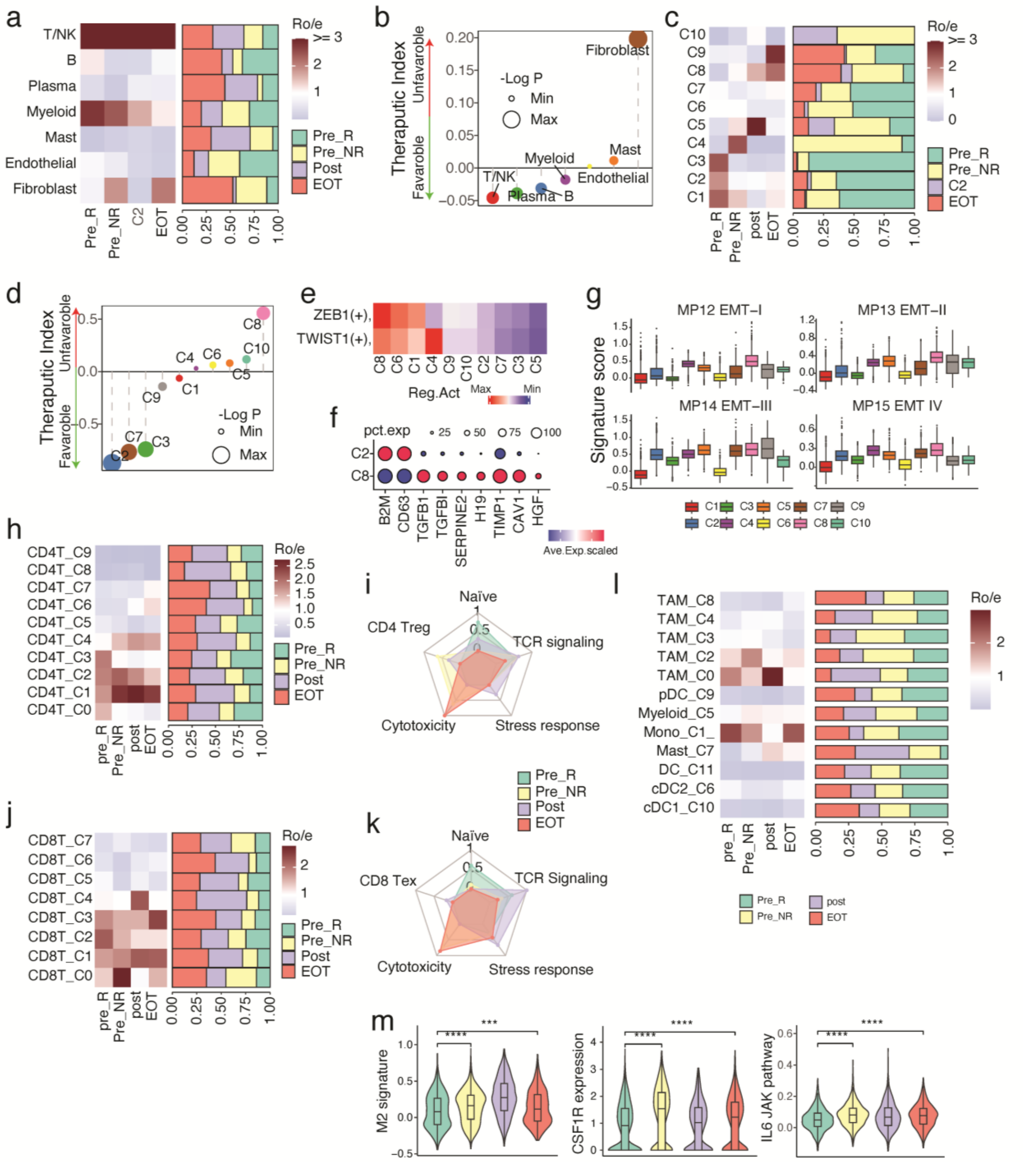


**Supplementary Figure S8.** Longitudinal single-cell transcriptomic changes during trial therapy in tumor samples from the 8 patients enrolled in cohort 4 as listed in Supplementary Table S13. (**a**) Distribution of tumor microenvironment (TME) cell clusters across groups. The heat map on the left shows tissue prevalence estimated by the ratio of observed to expected cell number (Ro/e). The bar plot on the right shows the relative proportion of cells from all patients for each TME cell subset. Pre_R (n=2; baseline samples from patients that demonstrated an objective response to trial therapy), Pre_NR (n=6; baseline samples from patients that did not demonstrate an objective response to trial therapy),

Post (n=3; samples collected at the C2 timepoint), and EOT (n=2; samples collected at the EOT timepoint). (**b**) Therapeutic index of major immune cell types in clear cell renal cell carcinoma (ccRCC) tumors treated with sitravatinib + nivolumab + ipilimumab. Dot size represents the significance evaluated by −Log10 (p value). (**c**) Distribution of malignant cell clusters across groups. The heat map on the left shows tissue prevalence estimated by the ratio of observed to expected cell number (Ro/e). The bar plot on the right shows the relative proportion of cells from all patients for each malignant cell subset. (**d**) Therapeutic index of malignant cell clusters in ccRCC tumors treated with sitravatinib + nivolumab + ipilimumab. Dot size represents the significance evaluated by −Log10 (p value). (**e**) Selected EMT transcription factor activities between different malignant cell clusters. (**f**) Bubble plot showing key marker gene expression between the C2 and C8 cell clusters. (**g**) Expression levels of selected EMT related meta-program signatures across malignant cell clusters. N = 1370 cells for C1, 1079 cells for C2, 1017 cells for C3, 710 cells for C4, 707 cells for C5, 699 cells for C6, 635 cells for C7, 647 cells for C8, 763 cells for C9, and 105 cells for C10. Box-and-whisker plots show all values with range (whiskers), interquartile range (box) and median (center line). (**h**) Distribution of CD4+ T cell clusters across groups. The heat map on the left shows tissue prevalence estimated by the ratio of observed to expected cell number (Ro/e). The bar plot on the right shows the relative proportion of cells from all patients for each CD4+ T cell subset. (**i**) Radar plot showing enrichment of selected five CD4+ T cell states. (**j**) Distribution of CD8+ T cell clusters across groups. The heat map on the left shows tissue prevalence estimated by the ratio of observed to expected cell number (Ro/e). The bar plot on the right shows the relative proportion of cells from all patients for each CD8+ T cell subset. (**k**) Radar plot showing enrichment of selected five CD8+ T cell states. (**l**) Distribution of myeloid cell clusters across groups. The heat map on the left shows tissue prevalence estimated by the ratio of observed to expected cell number (Ro/e). The bar plot on the right shows the relative proportion of cells from all patients for each myeloid cell subset. (**m**) Expression levels of M2 macrophage related signature, CSF1R gene, IL6 JAK pathway in macrophage and monocytes cell clusters between pre and post treatment groups. N = 1406 cells for Pre_R, 1404 cells for Pre_NR, 1076 cells for Post, and 838 cells for the EOT timepoint. *** : p-value < 0.001 ; **** : p-value < 0.0001. Source data are provided in the Source Data file.

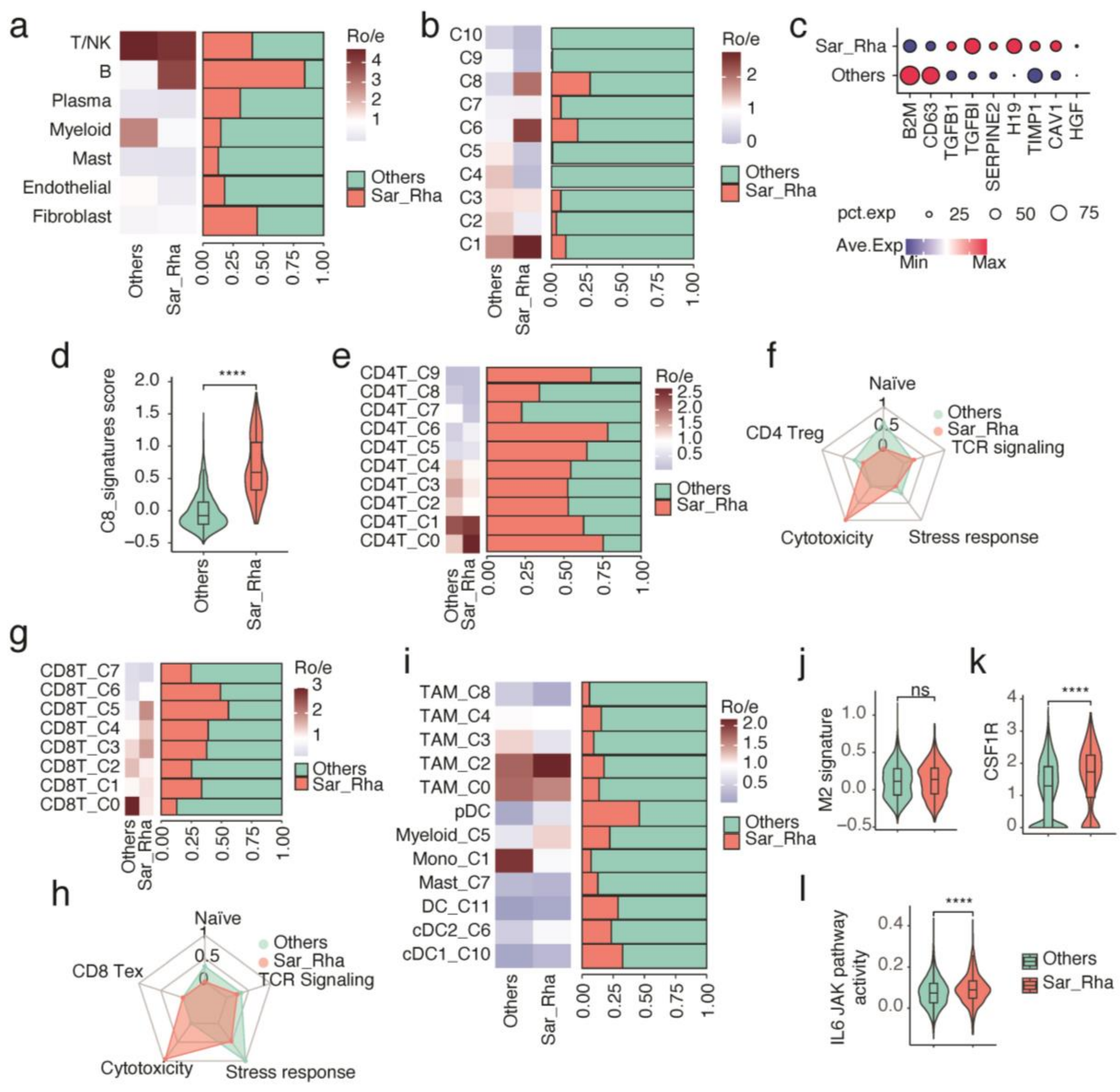


**Supplementary Figure S9.** Single-cell transcriptomic differences in baseline clear cell renal cell carcinoma (ccRCC) with sarcomatoid and/or rhabdoid dedifferentiation ("Sar_Rha"; n=3 patients) compared with baseline ccRCC without sarcomatoid or rhabdoid dedifferentiation ("Others"; n=9 patients) as listed in Supplementary Table S13. (**a**) Changes in distribution of tumor microenvironment (TME) cell clusters between Sar_Rha versus Others. The heat map on the left shows tissue prevalence estimated by the ratio of observed to expected cell number (Ro/e). The bar plot on the right shows the relative proportion of cells from all patients for each TME cell subset. (**b**) Changes in the distribution of malignant cell clusters between Sar_Rha versus Others. The heat map on the left shows tissue prevalence estimated by the ratio of observed to expected cell number (Ro/e). The bar plot on the right shows the relative proportion of cells from all patients for each malignant cell subset. (**c**) Bubble plot

showing differences in expression of key marker genes from the C2 and C8 cell clusters between Sar_Rha versus Others. (**d**) Expression levels of C8 signature in malignant cells in Sar_Rha and Others. N = 7810 cells for Others and 573 cells for Sar_Rha. (**e**) Distribution of CD4+ T cell clusters in Sar_Rha and Others. The heat map on the left shows tissue prevalence estimated by the ratio of observed to expected cell number (Ro/e). The bar plot on the right shows the relative proportion of cells from all patients for each CD4+ T cell subset. (**f**) Radar plot showing enrichment of selected five CD4+ T cell states in Sar_Rha and Others. (**g**) Distribution of CD8+ T cell clusters in Sar_Rha and Others. The heat map on the left shows tissue prevalence estimated by the ratio of observed to expected cell number (Ro/e). The bar plot on the right shows the relative proportion of cells from all patients for each CD8+ T cell subset. (**h**) Radar plot showing enrichment of selected five CD8+ T cell states in Sar_Rha and Others. (**i**) Distribution of myeloid cell clusters in Sar_Rha and Others. The heat map on the left shows tissue prevalence estimated by the ratio of observed to expected cell number (Ro/e). The bar plot on the right shows the relative proportion of cells from all patients for each myeloid cell subset. (**j**) Expression levels of M2 macrophage related signature in macrophage and monocytes cell clusters of Sar_Rha versus Others. N = 5618 cells for Others and 973 cells for Sar_Rha. (**k**) Expression levels of CSF1R gene in macrophage and monocytes cell clusters of Sar_Rha versus Others. N = 5618 cells for Others and 973 cells for Sar_Rha. (**l**) Expression levels of IL6-JAK pathway genes in macrophage and monocytes cell clusters of Sar_Rha versus Others. N = 5618 cells for Others and 973 cells for Sar_Rha. **** : p-value < 0.0001 ; ns: not significant. Source data are provided in the Source Data file.

## Supplementary Note

## Clinical Trial Protocol

# CLINICAL RESEARCH PROTOCOL

| | |
|---|---|
| **DRUGS:** | Sitravatinib (MGCD516)<br>Nivolumab (OPDIVO®)<br>Ipilimumab (YERVOY®) |
| **STUDY NUMBER:** | 516-008 |
| **PROTOCOL TITLE:** | A Phase 1/1b Study of Sitravatinib in Combination with Nivolumab and Ipilimumab in Patients with Advanced or Metastatic Clear-Cell Renal Cell Carcinoma or Other Solid Malignancies |
| **IND NUMBER:** | 138695 |
| **SPONSOR:** | Mirati Therapeutics, Inc.<br>9393 Towne Centre Drive, Suite 200<br>San Diego, California, 92121, USA |
| **ORIGINAL PROTOCOL DATE:** | 21 Feb 2020 |
| **VERSION NUMBER:** | V2.0 |
| **VERSION DATE:** | 30 March 2021 |

CONFIDENTIALITY STATEMENT

This document contains confidential information and/or trade secrets of Mirati Therapeutics, Inc. As such, this document is protected from unauthorized disclosure or use by confidentiality agreement(s) and/or is exempt from public disclosure pursuant to applicable FDA and foreign counterpart regulations.

# SPONSOR INFORMATION

Refer to the Study Manual for complete contact information.

# DOCUMENT HISTORY

| Document | Version Date | Summary of Changes |
|---|---|---|
| Original Protocol, Version 1.0 | 21 Feb 2020 | NA |
| Amendment #1 Version 2.0 | 30 Mar 2021 | Executive summary of main changes in Amendment #1:<br>• Incorporated information from Protocol Administrative letters dated 01 April 2020, 17 June 2020, 07 July 2020, and 11 March 2021. Information from protocol administrative letter dated 21 August 2020 is no longer effective; correct guidance on timing of administering gastric acid medications relative to sitravatinib is given.<br>• Introduced 2 dose de-escalation levels of ipilimumab.<br>• Revised to allow for enrollment of additional patients at Phase 1 dose escalation regimens that are potentially viable Phase 1b regimens to ensure sufficient safety experience and early evidence of clinical activity are available within the previously stated Phase 1 approximate sample size.<br>• Revised to allow for escalation of sitravatinib to 100 mg QD in the event of nivolumab and ipilimumab discontinuation or maintenance nivolumab discontinuation due to adverse events. Dosing at 100 mg QD is both the RP2D for sitravatinib monotherapy and for combinations with PD-(L)1 immune checkpoint inhibitors.<br>• Removed reference to sitravatinib free base formulation, as all patients in this study have received and will continue to receive the sitravatinib malate formulation only.<br>• Addressed clerical errors and made minor clarifications throughout the protocol. |

# STUDY SYNOPSIS

**Title:** A Phase 1/1b Study of Sitravatinib in Combination with Nivolumab and Ipilimumab in Patients with Advanced or Metastatic Clear-Cell Renal Cell Carcinoma or Other Solid Malignancies

**Rationale:** The use of tyrosine kinase inhibitors (TKIs) to treat cancer is well established based on robust clinical efficacy achieved with well tolerated inhibitors directed toward oncogenic tyrosine kinases. Select TKIs have been shown to modulate the immunogenic status of tumors, improve tumor perfusion by reducing intratumoral pressure and modulate subsets of immune cells, thereby increasing the frequency and function of effector immune elements while decreasing the number and function of immune suppressor cells.

Sitravatinib is a spectrum-selective receptor tyrosine kinase (RTK) inhibitor that inhibits several closely related RTKs including the TAM family (Tyro3/Axl/MERTK), VEGFR2, KIT, and MET. RTKs are key regulators of signaling pathways leading to cell growth, survival, and migration. These kinases are dysregulated in many cancers through overexpression, genetic alteration or co-expression with high affinity ligands (Blume-Jensen-2001). Multiple sitravatinib RTK targets are genetically altered in a variety of cancers and act as oncogenic drivers, promoting cancer development and progression. In addition to the immunostimulatory effects of Axl and MET inhibition, sitravatinib may further condition the tumor microenvironment (TME) in favor of antitumor activity by its immunomodulatory effects mediated through VEGFR and KIT inhibition.

The current study is designed to evaluate the safety and antitumor activity of a novel, triple-drug combination of sitravatinib and the well-established combination regimen of checkpoint inhibitor therapies (CITs) nivolumab and ipilimumab (NIVO/IPI). Combining sitravatinib and NIVO/IPI is predicted to have complementary effects in triggering a tumor-directed immune response.

Nivolumab and ipilimumab are monoclonal antibodies (mAbs) that inhibit the immune checkpoint proteins programmed death receptor-1 (PD-1) and cytotoxic T-lymphocyte antigen-4 (CTLA-4), respectively. As negative regulators of T-cell activation, PD-1 and CTLA-4 both play important, nonredundant roles in the co-inhibitory mechanisms of immune responses.

CTLA-4 limits T-cell activation and clonal expansion, whereas PD-1 primarily inhibits effector T-cell function in tissues. Both proteins have been targeted clinically for their potential role in dysregulation of tumor immunogenicity, and the combination of PD-1 and CTLA-4 blockade has been investigated as a way to increase antitumor activity compared to inhibition of either protein alone. Preclinical data have shown that the combined activity of nivolumab (anti-PD-1) and ipilimumab (anti-CTLA-4) leads to enhanced T-cell function in a mixed lymphocyte reaction and increased antitumor activity in several syngeneic mouse systems compared with the effects of either antibody alone (OPDIVO USPI-2019).

The combination of nivolumab and ipilimumab was first approved by the United States (US) Food and Drug Administration (FDA) in 2015 for first-line treatment of melanoma, with improved response rates for the combination regimen compared with either agent alone (OPDIVO USPI-2019; YERVOY USPI-2019). NIVO/IPI treatment is also approved for first-line treatment of intermediate/poor-risk clear-cell renal cell carcinoma (ccRCC). Favorable activity in several other cancer types has been observed with NIVO/IPI treatment as well, including favorable-risk ccRCC (Hammers-2017), advanced hepatocellular carcinoma (HCC) (Yau-2019), metastatic urothelial cancer (UC) (Sharma-2019), and non-small cell lung cancer (NSCLC) (Hellman-2017).

Single-agent sitravatinib has demonstrated activity in VEGF-refractory RCC, and is also being evaluated in several cancer types in combination with PD-1 inhibitors in patients that have progressed on or were refractory to single agent PD-1 pathway inhibitors. Preliminary clinical activity with this combination has been observed in NSCLC, RCC, and UC. Sitravatinib plus the PD-1 inhibitor nivolumab is also being tested in the checkpoint inhibitor naïve setting based on the rationale and the activity observed in the checkpoint inhibitor-refractory setting.

The mechanistic rationale supporting the combination of a PD-1 inhibitor plus a CTLA-4 inhibitor is distinct and largely non-overlapping with the rationale supporting the combination of a PD-1 inhibitor plus sitravatinib. CTLA-4 is a checkpoint expressed on T-cell populations whereas sitravatinib reverses the immunosuppressive function of several innate immune cell types, including macrophages, dendritic cells and myeloid-derived suppressor cells (MDSCs), in addition to regulatory T-cell (Tregs). Given the distinct immune suppressive mechanisms targeted by

| | |
|---|---|
| | CTLA-4 antibodies and sitravatinib, coupled with the observation that both agents effectively augment PD-1 inhibition, the triple combination of nivolumab, sitravatinib and ipilimumab is a rational strategy to treat several cancer types in which PD-1 inhibitors are clinically active as single agents but in a limited number of patients.<br><br>Based on these findings, the current study is designed to evaluate the triple combination of sitravatinib plus NIVO/IPI in patients with solid tumor malignancies that have shown favorable responses to NIVO/IPI combinations in previous clinical trials.<br><br>Initially the study will evaluate sitravatinib plus the fixed regimen NIVO3/IPI1 (3 mg/kg intravenous [IV] nivolumab and 1 mg/kg IV ipilimumab every 3 weeks [Q3W] for 4 doses, then IV nivolumab 240 mg every 2 weeks [Q2W] or 480 mg every 4 weeks [Q4W]) as first-line therapy in patients with intermediate/poor-risk advanced or metastatic ccRCC. The NIVO3/IPI1 regimen is approved for treatment of this patient population. Sitravatinib dose escalation cohorts begin at 35 mg once daily (QD), a dose well below the RP2D of sitravatinib in combination with nivolumab. |
| **Target Population:** | Patients with advanced or metastatic ccRCC or other solid malignancies (indications in which favorable activity has been previously demonstrated with nivolumab/ipilimumab combination treatment). |
| **Number in Trial:** | Phase 1 Dose Escalation: Approximately 30 patients to achieve approximately 27 patients evaluable for dose-limiting toxicity (DLT).<br><br>Phase 1b Dose Expansion: Up to 31 patients per cohort. |
| **Primary Objective:** | • To evaluate the safety and tolerability of sitravatinib in combination with nivolumab and ipilimumab in the study populations. |
| **Secondary Objectives:** | • To evaluate the clinical activity of sitravatinib in combination with nivolumab and ipilimumab.<br><br>• To evaluate the pharmacokinetics (PK) of sitravatinib when administered in combination with nivolumab and ipilimumab. |
| **Exploratory Objectives:** | • To assess the effects of the combination regimen on tumor cell expression of PD-L1, tumor infiltrating immune cell populations, and gene expression signatures. |

| | |
|---|---|
| | • To assess correlations between treatment-related outcomes and tumor immune biomarkers and tumor gene mutations. |
| **Primary Endpoint:** | • Safety characterized by type, incidence, severity, timing, seriousness, and relationship to study treatment of adverse events (AEs), and laboratory abnormalities. |
| **Secondary Endpoints:** | • Clinical Activity Endpoints:<br>○ Objective response rate (ORR) as defined by Response Evaluation Criteria in Solid Tumors version 1.1 (RECIST 1.1);<br>○ Duration of response (DOR);<br>○ Clinical benefit rate (CBR);<br>○ Progression-free survival (PFS);<br>○ One-year survival rate; and<br>○ Overall survival (OS).<br>• Blood plasma concentrations of sitravatinib. |
| **Exploratory Endpoints:** | • Tumor expression of programmed death-ligand 1 (PD-L1);<br>• Immune cell populations in the tumor;<br>• Gene expression signatures in the tumor;<br>• Tumor gene alterations; and<br>• Circulating tumor deoxyribonucleic acid (ctDNA). |
| **Study Design:** | Study 516-008 is an open-label Phase 1 dose escalation/Phase 1b dose expansion study evaluating the safety and tolerability, clinical activity, and PK of sitravatinib in combination with nivolumab and ipilimumab for the treatment of ccRCC and potentially other solid tumor types. The Schedules of Assessments are provided in Table 1, Table 2, and Table 3. |

**Phase 1 Dose Escalation**

The study begins with Phase 1 dose escalation of sitravatinib administered in combination with the NIVO3/IPI1 regimen as first-line therapy in patients with intermediate or poor-risk ccRCC. The starting dose for sitravatinib is 35 mg QD (Cohort 1), and planned dose escalation levels are outlined in Table 4.
Dose escalation of sitravatinib will follow the time-to-event Bayesian optimal interval (TITE-BOIN) design described in Section 9.

If a tolerable dose is identified for sitravatinib in combination with NIVO3/IPI1, the combination of sitravatinib and the alternate regimen NIVO1/IPI3 (1 mg/kg IV nivolumab and 3 mg/kg IV ipilimumab Q3W for 4 doses, then IV nivolumab 240 mg Q2W or 480 mg Q4W may be evaluated through dose escalation via a protocol amendment. In addition, dose de-escalation levels for ipilimumab may be evaluated as outlined in Table 5. Decisions to remain at the current dose level may override decisions to dose escalate per TITE-BOIN design, based on clinical judgment of the Principal Investigator and in consultation with the Sponsor

As potentially viable Phase 1b regimens are identified, additional patients may be enrolled into Phase 1 dose escalation regimens at or below the MTD to ensure sufficient safety experience and/or early evidence of clinical activity are available to recommend Phase 1b regimens. Phase 1 dose escalation and enrollment of additional patients at prior dose levels may proceed in parallel after safety evaluations are sufficiently mature.

**Phase 1b Dose Expansion**

The Phase 1b portion of the study will be initiated following the identification of the recommended dose of sitravatinib in combination with nivolumab and ipilimumab. Depending on aggregate data from the escalation and expansion cohorts, lower doses of sitravatinib in combination with nivolumab and ipilimumab may also be explored during Phase 1b, if warranted.

Initially, the planned dose expansion cohorts are designed to evaluate first-line treatment in patients with intermediate/poor-risk ccRCC (Cohort A) and first-line treatment in patients with favorable-risk ccRCC (Cohort B). The decision to initiate Cohort B will be determined based on Phase 1 dose-escalation findings

| | |
|---|---|
| | and as information becomes available supporting the NIVO3/IPI1 regimen in favorable-risk RCC.<br><br>Future dose expansion cohorts may include other solid malignancies in which favorable activity has been previously demonstrated with nivolumab/ipilimumab combination treatment, such as metastatic colorectal cancer (CRC), melanoma, HCC, NSCLC, and UC. These cohorts would be added via protocol amendment as information supporting expansion of the study becomes available. |
| **Study Treatments:** | All patients will receive sitravatinib, nivolumab, and ipilimumab. Study treatment will be administered as 21-day cycles for Cycles 1-4 and 28-day cycles for Cycles 5+. Sitravatinib capsules are taken PO QD. Nivolumab and ipilimumab will be administered by IV infusion (on the same day) Q3W for Cycles 1-4, followed by nivolumab Q2W or Q4W for Cycles 5+ in accordance with the United States Prescribing Information (USPI) and standard care.<br><br>Phase 1 dose escalation levels are shown in Table 4 for sitravatinib. Dosing will begin at 35 mg QD in Cohort 1 in combination with the fixed NIVO3/IPI1 regimen as shown in Table 5.<br><br>Guidelines for study drug administration and dose modification in the event of toxicity are provided in Section 5. If nivolumab or ipilimumab is interrupted or discontinued, administration of sitravatinib may be continued at the discretion of the Investigator and patient as outlined in Section 5. In the event of treatment interruption/delay, the original cycle length (ie, 21 days for Cycles 1-4, 28 days for Cycles 5+) should be maintained unless all 3 study treatments are interrupted. In this case, the next cycle should start at the resumption of any of the 3 study treatments.<br><br>Patients may continue to receive study treatment at the discretion of the Investigator until disease progression, unacceptable AEs, patient refusal, or death. Patients experiencing clinical benefit in the judgment of the Investigator may continue study treatment beyond disease progression as defined by RECIST 1.1 if the progression is not rapid, symptomatic, or requiring urgent medical intervention. Patients considering continuation of study treatment beyond RECIST-defined disease progression must be provided with and sign an informed consent form (ICF) outlining other available therapies and any potential clinical benefit that the patient may be foregoing by continuing study treatment. |

| | |
|---|---|
| | Patients discontinuing treatment will be followed for subsequent anticancer therapies and survival. |
| **Pharmacokinetic Evaluation:** | The PK of sitravatinib will be evaluated using blood samples collected at specified time points prior to and following study treatment dosing. Every effort will be made to collect these PK samples at the exact nominal times relative to dosing. A variation window is allowed for each time point as outlined in Table 2. |
| **Molecular Marker Evaluation:** | Molecular markers to be investigated include ctDNA assessment and optional tumor tissue assessments in a subset of patients at select sites as specified in Table 1 and Table 3. Tumor tissue assessments include PD-L1 expression, immune cell populations, gene expression signatures, and tumor gene alterations. |
| **Statistical Considerations:** | **Phase 1 Dose Escalation**<br><br>The time-to-event Bayesian optimal interval (TITE-BOIN) design (Yuan-2018) will be used to determine whether sequential dose escalation/de-escalation steps for sitravatinib in combination with nivolumab and ipilimumab described in Table 15 should be undertaken and to identify the maximum tolerated dose (MTD). The TITE-BOIN design is well-suited for use in dose escalation studies involving treatments associated with late-onset toxicity. The TITE-BOIN design allows dose escalation decisions for new patients while some patients continue evaluation for DLT at the previous dose level, thus shortening the overall duration of the trial.<br><br>The TITE-BOIN model to be implemented in the current study is based on the following assumptions:<br><br>• The MTD is defined to have 0.3 probability of DLT;<br>• Initial cohort size is 3 patients;<br>• The overall duration of DLT assessment window is 9 weeks.<br><br>The approximate sample size in the Phase 1 segment of the study is 27 DLT-evaluable patients (refer to Section 9.3.5).<br><br>Appendix 3 describes how dose escalation/de-escalation decisions will be reached during conduct of the Phase 1 portion of this study. Such decisions will be made by the Sponsor in collaboration with |

Investigators and will be communicated to sites via written correspondence prior to implementation. At the completion of the dose escalation portion of the study, the MTD will be identified based on isotonic regression as specified in Yuan-2018.

**Phase 1b Dose Expansion**

Time-to-event Bayesian Optimal Phase 2 (TOP) design will be used for the Phase 1b portion of study. The null hypothesis of 30% true response rate will be tested against alternative hypothesis of 50% response rate. The go/no-go decision at interim will be based on the totality of the data and the posterior probability of $Pr(p_{eff} > 0.3|data)$. If $Pr(p_{eff} > 0.3|data) < \lambda(\frac{n}{N})^{\alpha}$, then potentially stop the study at interim. Otherwise, continue the study. $\lambda$=0.84 and $\alpha$=1 are design parameters optimized to maximize the power under the alternative hypothesis; n and N are interim sample size and maximum sample size. A Beta (0.3,0.7) prior distribution is used to calculate posterior probability of $Pr(p_{eff} > 0.3|data)$. Interim analysis will be conducted after 14 patients are enrolled and treated in each Phase 1b cohort. The go/no-go decision rule is outlined in Table 13.

## Table 1: Schedule of Assessments: Screening and Treatment

Table 1 provides an overview of the protocol visits and procedures. Refer to Sections 6 and 7 for detailed information. Additional, unplanned assessments should be performed as clinically indicated, including for the purpose of fully evaluating AEs.

| **Assessments** | **Screen/ Baseline** | **Cycle 1 (21-day cycle)** | | | **Cycles 2-4 (21-day cycles)** | **Cycles 5+ (28-day cycles)** | |
|---|---|---|---|---|---|---|---|
| | **Within 28 days** | **Day 1** | **Day 8 (± 2 days)[3]** | **Day 15 (± 2 days)** | **Day 1 (± 2 days)** | **Day 1 (± 2 days)** | **Day 15 (± 2 days)** (Only if Q2W NIVO) |
| Study Participation Informed Consent[1] | Before study-specific assessments | | | | | | |
| Medical & Disease History, Prior Therapy | X | | | | | | |
| Collection of Archival Tissue, if available[2] | X | | | | | | |
| Blood sample for ctDNA | X | | | | | | |
| ECOG Performance Status | X | | | | X Cycle 3 only | X[3] Cycle 5 only | |
| Physical Exam[4] | X | | | | | | |
| Abbreviated Physical Exam[4] | | X | X[3] | X | X | X | X |
| Vital Signs[5] | X | X | X[3] | X | X | X | X |
| Pregnancy Test[6] | X | As clinically indicated | | | | | |
| Coagulation[7] | X | As clinically indicated | | | | | |
| Urinalysis[7] | X | As clinically indicated | | | | | |
| Hematology[7] | X | X[8] | X[3] | X | X | X | |
| Serum Chemistry[7] | X | X[8] | X[3] | X | X | X | |
| Thyroid Function Test[7] | | X[8] | | | X | X | |

| | **Screen/ Baseline** | **Cycle 1 (21-day cycle)** | | | **Cycles 2-4 (21-day cycles)** | **Cycles 5+ (28-day cycles)** | |
|---|---|---|---|---|---|---|---|
| **Assessments** | **Within 28 days** | **Day 1** | **Day 8 (± 2 days)[3]** | **Day 15 (± 2 days)** | **Day 1 (± 2 days)** | **Day 1 (± 2 days)** | **Day 15 (± 2 days)** (Only if Q2W NIVO) |
| ECHO (preferred) or MUGA Scan | X (-35 days) | | | | Cycle 3 Day 1, and as clinically indicated | | |
| Disease Evaluation[9] | X | | | | | Week 13 (±10 days); then every 8 weeks (±10 days) for 12 months; then every 16 weeks | |
| Fresh Tumor Tissue Biopsy (optional)[10] | X | | | | Cycle 2 only (±7 days) | After PD but before other anticancer therapy | |
| Single 12-Lead ECG[11] | X | As clinically indicated | | | | | |
| Triplicate 12-Lead ECG[11] | | See Table 2 | | | | | |
| Blood samples for PK[12] | | See Table 2 | | | | | |
| Sitravatinib Dispensing and Reconciliation | | X | | | X | X | |
| Nivolumab Administration | | Throughout as directed in USPI and protocol | | | | | |
| Ipilimumab Administration | | Throughout as directed in USPI and protocol | | | | | |
| Adverse Events[13] and Concomitant Meds | SAEs only | Throughout | | | | | |

Abbreviations: ctDNA = circulating tumor DNA; ECG = electrocardiogram; ECHO = echocardiogram; ECOG = Eastern Cooperative Oncology Group; MUGA = multigated acquisition; NIVO = nivolumab; PD = progressive disease; PK = pharmacokinetics; Q2W = every 2 weeks; SAEs = serious adverse events; USPI = United States Prescribing Information.

1. Study Participation Informed Consent: May be performed more than 28 days prior to enrollment and must be completed prior to initiation of any study specific assessments.
2. Archival tumor tissues (if available) are to be taken from patients who opt out of on-study tumor biopsies.
3. Cycle 1/Day 8 Visit and select ECOG assessment (Cycle 5 Day1) are not required for patients enrolled in Phase 1b Dose Expansion cohorts.
4. Physical Examinations: A complete physical examination is required at Screening. Height will be recorded at Screening only. All other physical examinations during treatment will be symptom-directed, abbreviated evaluations.
5. Vital Signs: Weight, temperature, blood pressure, and pulse rate to be assessed prior to dosing and PK blood sampling, as applicable. Refer to Table 2.

6. Pregnancy Test: If the patient is a woman of childbearing potential, negative serum or urine pregnancy test performed by the local laboratory at screening will be required. The informed consent process must include discussion of the risks associated with pregnancy and adequate contraception methods. Additional pregnancy testing may be necessary if required by local practices or regulations, or if potential pregnancy is suspected.
7. Safety Laboratory Assessments: Hematology, coagulation, chemistry, thyroid function, and urinalysis evaluations (see Table 10) will be performed by local laboratories. Lab assessments may be performed up to 3 days prior to clinic visits.
8. Selected Day 1 Assessments: Repeat assessment not required if screening assessment performed within 7 days before the first dose of study treatment.
9. Disease Evaluations: To be performed at Screening (-28 day window); at Week 13 (±10 days); then every 8 weeks (±10 days) until Week 49 (~12 months); and then every 16 weeks until objective disease progression or the start of subsequent anticancer therapy, whoever occurs first. All on-study disease evaluations should be based on a calendar beginning from the first day of dosing. Computed tomography (CT) scans should be performed with contrast agents unless contraindicated for medical reasons. If IV contrast is medically contraindicated, the imaging modality to be used (either CT without contrast or magnetic resonance imaging [MRI]) should be the modality that best evaluates the disease. The same imaging modality should be used for an individual patient throughout their participation in the study. Refer to Section 7.1.1 for additional guidance. Patients who discontinue the study for reasons other than objective disease progression will continue to have disease assessments at the same frequency until radiologically confirmed progression, initiation of new anticancer therapy, or death.
10. Collection of Fresh Tumor Tissue (optional): At select sites, serial biopsies of tumor tissue will be collected at Baseline, at Cycle 2 Day 1 (±7 days), and at disease progression (clinical or objective). The collection at disease progression is only for patients who have a confirmed response (partial response [PR] and/or confirmed response [CR]) and should be performed as close as possible to the determination of progression, but before the beginning of subsequent anticancer therapy. Tumor tissue samples will be used for mutation gene analysis, immune marker analysis, and gene expression profiling. Collection of optional tissue biopsies will begin at dose regimens that are potentially viable for Phase 1b dose expansion.
11. 12-Lead ECGs: Triplicate ECGs will accompany PK sampling as described in Table 2. In addition, single 12-lead ECGs are to be performed as clinically indicated. Assessments will include an evaluation of heart rate, QT, and QTc intervals. RR interval should be recorded during each ECG assessment in order to calculate QTcF.
12. Pharmacokinetic Samples: PK blood samples to be collected after ECGs and assessment of vital signs as scheduled in Table 2.
13. Adverse Events: SAEs will be reported from the time of informed consent until at least 28 days after the last dose of study treatment, or until the start of subsequent anticancer therapy, whichever occurs first. Non-serious AEs will be reported from the first dose of study treatment until at least 28 days after the last dose of study treatment, or until the start of subsequent anticancer therapy, whichever occurs first. The reporting period for all immune-related AEs (irAEs) will continue until at least 100 days after the last dose of study treatment, or until the start of subsequent anticancer therapy, whichever occurs first. All ongoing SAEs will be followed until resolution or stabilization (see Section 8.4).

## Table 2: Schedule of PK Sample Collection and Triplicate ECGs

| Procedure | Cycle 1 | | | | | | | | Cycles 2, 4, 6, 8 |
|---|---|---|---|---|---|---|---|---|---|
| | Day 1 | | | Day 2 | Day 15 | | | Day 16 | Day 1 |
| | Predose (-30 to 0 min) | 30 min ±10 min | 7 h ±2 h | Predose (-30 to 0 min) | Predose (-30 to 0 min) | 30 min ±10 min | 7 h ±2 h | Predose (-30 to 0 min) | Predose (-30 to 0 min) |
| PK Blood Sample[1,2] | X | X[3] | X[3] | X[3,4] | X[3] | X[3] | X[3] | X[3,4] | X |
| Triplicate ECG[5] | X[6] (-60 min) | | X[3] | | X[3] | | X[3] | | X |

Abbreviations: ECG = electrocardiogram; h = hour(s); min = minute(s); PK = pharmacokinetics.

Note: For all scheduled PK sampling days, patients should be instructed to withhold their daily dose of sitravatinib, as this dose will be taken at the clinic visit according to the timing indicated in Table 2.

1. Scheduled vital signs and triplicate ECGs precede PK blood sample collection in all cases. In addition, sitravatinib dosing, predose PK blood sampling, and 30 min postdose PK blood sampling should precede nivolumab and ipilimumab infusions.
2. In addition to the scheduled samples, an unscheduled PK blood sample should be drawn before a daily sitravatinib dose (trough sample) in the event of any of the following events: 1) as soon as possible after an SAE, and 2) at a clinic visit at least one week following a dose reduction of sitravatinib.
3. For Phase 1 dose escalation patients only.
4. The predose PK blood samples on Cycle 1 Day 2 and Day 16 should be taken 24 hours after the previous dose of sitravatinib.
5. ECGs should be taken in triplicate, each reading approximately 2 minutes apart. One set of triplicate ECGs is required at all other time points. In general, ECGs should be performed prior to the respective PK blood collection. Examples of the schedule are presented below:
   - Example for Cycle 1 Day 1 predose ECG/PK: ~ -60 min (triplicate ECGs); ~ -30 min (triplicate ECGs, if required); ~ -15 min (vitals/PK).
   - Example for all other predose ECG/PK assessments: ~ -30 min (triplicate ECGs); ~ -15 min (vitals/PK).
6. For the first triplicate ECG for each patient collected at baseline, a second set of triplicate ECGs should be done within 1 hour prior to dosing (eg, at -30 min prior to dosing) for those patients who have a difference in any two QT measurements of 15 msec or more in order to firmly establish the baseline for the patient.

## Table 3: Schedule of Assessments: Post-Treatment

Table 3 provides an overview of protocol visits and procedures. Refer to Sections 6 and 7 for detailed information. Additional, unplanned assessments should be performed as clinically indicated, including for the purpose of fully evaluating AEs.

| Assessments | Follow-up Visits | | | Long-term Follow-up |
|---|---|---|---|---|
| | 7-Day Visit[1] | 28-Day Visit[2] | 100-Day Visit[3] | |
| ECOG Performance Status | X | | | |
| ECHO/MUGA Scan | X | | | |
| Single 12-Lead ECG[4] | X | | | |
| Physical Exam | X | | | |
| Abbreviated Physical Exam[5] | | X | X | |
| Vital Signs[6] | X | X | X | |
| Hematology[7] | X | | | |
| Serum Chemistry[7] | X | | | |
| Thyroid Function Test[7] | X | | | |
| Coagulation[7] | As clinically indicated | | | |
| Urinalysis[7] | As clinically indicated | | | |
| Pregnancy Test[8] | As clinically indicated | | | |
| Fresh Tumor Tissue Biopsy (optional)[9] | After PD but before other anticancer therapy | | | |
| Adverse Events[10] | X | X | X | |
| Survival and Subsequent Anticancer Therapy[11] | | | | X |

Abbreviations: ECG = electrocardiogram; ECHO = echocardiogram; ECOG = Eastern Cooperative Oncology Group; MUGA = multigated acquisition; PD = progressive disease; SAEs = serious adverse events; USPI = United States Prescribing Information.

1. The 7-Day Visit is to occur within 7 days of the decision to stop all study treatment and prior to the beginning of subsequent anticancer therapy. Assessments completed in the previous 4 weeks do not need to be repeated, with the exception of AEs (which must be assessed at all visits), and hematology and chemistry (which must be assessed at this visit if not completed in the previous 10 days).

2. The 28-Day Visit is to occur at 28 (+7) days after the last dose of study treatment, or earlier if patient is to begin subsequent anticancer therapy.
3. The 100-Day Visit is to occur at 100 (+7) days after the last dose of study treatment, or earlier if patient is to begin subsequent anticancer therapy.
4. Single 12-lead ECG assessment will include an evaluation of heart rate, QT, and QTc intervals. RR interval should be recorded in order to calculate QTcF.
5. Physical Examinations: A complete physical examination is required at the 7-Day Visit. All other physical examinations will be symptom-directed, abbreviated evaluations.
6. Vital Signs include temperature, blood pressure, and pulse rate. Weight should also be measured.
7. Safety Laboratory Assessments: Hematology, coagulation, chemistry, thyroid function, and urinalysis evaluations (see Table 10) will be performed by local laboratories. Lab assessments may be performed up to 3 days prior to clinic visits.
8. Pregnancy Test: If the patient is a woman of childbearing potential, additional serum or urine pregnancy tests (performed by the local laboratory) may be necessary if required by local practices or regulations, or if potential pregnancy is suspected.
9. Collection of Fresh Tumor Tissue (optional): At select sites, serial biopsies of tumor tissue will be collected after disease progression but before the beginning of subsequent anticancer therapy. The collection at disease progression is only for patients who have a confirmed response (partial response [PR] and/or confirmed response [CR]) and should be performed as close as possible to the determination of progression, but before the beginning of subsequent anticancer therapy. Tumor tissue samples will be used for mutation gene analysis, immune marker analysis, and gene expression profiling. Collection of optional tissue biopsies will begin at dose regimens that are potentially viable for Phase 1b dose expansion.
10. Adverse Events: SAEs will be reported from the time of informed consent until at least 28 days after the last dose of study treatment, or until the start of subsequent anticancer therapy, whichever occurs first. Non-serious AEs will be reported from the first dose of study treatment until at least 28 days after the last dose of study treatment, or until the start of subsequent anticancer therapy, whichever occurs first. The reporting period for all immune-related AEs (irAEs) will continue until at least 100 days after the last dose of study treatment, or until the start of subsequent anticancer therapy, whichever occurs first. All ongoing SAEs will be followed until resolution or stabilization (see Section 8.4).
11. Survival status and subsequent anticancer therapy will be collected every 2 months (±14 days) from the 28-Day Visit until death or lost to follow up. May be performed by telephone or email.

# TABLE OF CONTENTS

CLINICAL RESEARCH PROTOCOL ....................1
SPONSOR INFORMATION ....................2
DOCUMENT HISTORY ....................2
STUDY SYNOPSIS ....................3
TABLE OF CONTENTS ....................17
TABLE OF TABLES ....................21
TABLE OF FIGURES ....................21
LIST OF ABBREVIATIONS ....................22
1 INTRODUCTION AND STUDY RATIONALE ....................26
1.1 Cancer Immunotherapy ....................26
1.1.1 Checkpoint Pathway Inhibition ....................26
1.1.1.1 Programmed Death Receptor-1 (PD-1) and Inhibition by Nivolumab ....................26
1.1.1.2 Cytotoxic T-Lymphocyte Antigen 4 (CTLA-4) and Inhibition by Ipilimumab ....................27
1.1.1.3 Dual Checkpoint Inhibition: Combining Nivolumab and Ipilimumab ....................27
1.1.2 Tyrosine Kinase Inhibition ....................28
1.1.2.1 Tyrosine Kinase Inhibition by Sitravatinib ....................28
1.1.2.2 Previous Experience with Sitravatinib ....................28
1.2 Study Rationale ....................32
1.2.1 Rationale for Combining Nivolumab, Ipilimumab, and Sitravatinib ....................32
1.2.1.1 Clinical Studies Combining Sitravatinib with Checkpoint Inhibitors ....................33
1.2.1.2 Expectations for Safety of the Combination of Nivolumab, Ipilimumab, and Sitravatinib ....................34
1.2.2 Rationale for Treatment in Renal Cell Carcinoma ....................36
1.2.2.1 NIVO/IPI First-Line Treatment of Patients with ccRCC ....................37
1.2.2.2 Sitravatinib Treatment of Patients with ccRCC ....................38
2 STUDY OBJECTIVES AND ENDPOINTS ....................39
2.1.1 Objectives ....................39
2.1.1.1 Primary Objectives ....................39
2.1.1.2 Secondary Objectives ....................39
2.1.1.3 Exploratory Objectives ....................39
2.1.2 Endpoints ....................39
2.1.2.1 Primary Endpoint ....................39
2.1.2.2 Secondary Endpoints ....................39
2.1.2.3 Exploratory Endpoints ....................40
3 STUDY DESIGN ....................40
3.1 Phase 1 Dose Escalation ....................41
3.2 Phase 1b Dose Expansion ....................41

4 SUBJECT SELECTION AND ENROLLMENT ....41
4.1 Inclusion Criteria ....42
4.2 Exclusion Criteria ....43
4.3 Life Style Guidelines ....46
4.4 Randomization ....46
5 STUDY TREATMENTS ....46
5.1 Study Treatment Overview ....46
5.2 Sitravatinib ....48
5.2.1 Sitravatinib Formulation, Packaging, and Storage ....48
5.2.2 Sitravatinib Preparation, Dispensing, and Accountability ....49
5.2.3 Sitravatinib Administration ....49
5.2.4 Sitravatinib Dose Modification or Discontinuation ....50
5.3 Nivolumab ....51
5.3.1 Nivolumab Formulation, Packaging, and Storage ....51
5.3.2 Nivolumab Preparation and Dispensing ....51
5.3.3 Nivolumab Administration ....51
5.3.4 Nivolumab Dose Modification or Discontinuation ....52
5.4 Ipilimumab ....52
5.4.1 Ipilimumab Formulation, Packaging, and Storage ....52
5.4.2 Ipilimumab Preparation and Dispensing ....52
5.4.3 Ipilimumab Administration ....52
5.4.4 Ipilimumab Dose Modification or Discontinuation ....52
5.5 Management of Adverse Events ....53
5.5.1 Sitravatinib-Related Adverse Events ....53
5.5.1.1 General Management of Non-Hematological Toxicities ....53
5.5.1.2 General Management of Hematological Toxicities ....54
5.5.1.3 Management of Selected Adverse Events ....54
5.5.2 Nivolumab Adverse Event Management Guidelines ....57
5.5.3 Ipilimumab Adverse Event Management Guidelines ....57
5.5.4 Management of Immune-Related Adverse Events ....57
5.5.4.1 Diarrhea/Colitis ....58
5.5.4.2 Increased Transaminases ....58
5.6 Assessment of Dose-Limiting Toxicity (Phase 1) ....59
5.6.1 DLT Definition for Non-immune Related AEs ....59
5.6.2 DLT Definition for Immune-Related AEs ....60
5.7 Medication Error ....61
5.8 Concomitant Therapies ....61
5.8.1 Concomitant Medications ....61
5.8.2 Concomitant Surgery or Radiation Therapy ....63
5.8.2.1 Cytoreductive Nephrectomy ....64
5.8.3 Other Anticancer or Experimental Therapy ....64
6 STUDY ASSESSMENTS ....64
6.1 Screening ....64
6.2 Study Period ....65
6.3 Follow-up Visits ....65

**6.4 Long-term Follow-up ..... 65**
**6.5 Patient Discontinuation/Withdrawal ..... 65**
**7 PROCEDURES ..... 66**
**7.1 Efficacy ..... 66**
**7.1.1 Radiographic Disease Assessment ..... 66**
**7.2 Safety Assessments ..... 67**
**7.2.1 Medical History ..... 67**
**7.2.2 Physical Examination, Vital Signs, and Performance Status ..... 68**
**7.2.3 Laboratory Safety Assessments ..... 68**
**7.2.4 Electrocardiograms ..... 69**
**7.2.5 Echocardiogram or Multigated Acquisition Scan ..... 69**
**7.3 Laboratory Evaluations ..... 69**
**7.3.1 Pharmacokinetic Evaluation ..... 69**
**7.3.2 Molecular Marker Evaluation ..... 70**
**7.3.2.1 Circulating Tumor DNA ..... 70**
**7.3.2.2 Markers in Tumor Tissue ..... 70**
**7.4 Post-treatment Follow-up ..... 71**
**8 ADVERSE EVENT REPORTING ..... 71**
**8.1 Sponsor Medical Monitor Personnel ..... 71**
**8.2 Adverse Events ..... 71**
**8.2.1 Laboratory Abnormalities ..... 72**
**8.2.1.1 Hy's Law ..... 72**
**8.2.2 Severity Assessment ..... 73**
**8.2.3 Causality ..... 73**
**8.3 Serious Adverse Events ..... 73**
**8.3.1 Definition of a Serious Adverse Event ..... 73**
**8.3.2 Exposure During Pregnancy ..... 74**
**8.4 Reporting of SAEs and AEs ..... 75**
**8.4.1 Reporting Period ..... 75**
**8.4.2 Reporting Requirements ..... 76**
**9 STATISTICS ..... 76**
**9.1 Hypotheses and Sample Size ..... 76**
**9.1.1 Phase 1 Dose Escalation ..... 76**
**9.1.2 Phase 1b Dose Expansion ..... 78**
**9.2 Data Handling ..... 80**
**9.3 Analysis Populations ..... 80**
**9.3.1 Enrolled Population ..... 80**
**9.3.2 Full Analysis Population ..... 80**
**9.3.3 Clinical Activity Evaluable Population ..... 80**
**9.3.4 Safety Population ..... 81**
**9.3.5 DLT-Evaluable Population ..... 81**
**9.3.6 Pharmacokinetic Evaluable Population ..... 81**
**9.3.7 Molecular Marker Evaluable Population ..... 81**
**9.4 Efficacy Endpoint Definitions and Analyses ..... 81**
**9.4.1 Objective Response Rate ..... 81**

9.4.2 Clinical Benefit Rate ....................81
9.4.3 Duration of Response ....................82
9.4.4 Progression-free Survival ....................82
9.4.5 Overall Survival ....................82
9.4.6 Subgroup Analyses ....................82
9.5 Safety Data Presentations and Summaries ....................82
9.5.1 Adverse Events ....................82
9.5.2 Prior and Concomitant Medications ....................83
9.5.3 Clinical and Laboratory Assessments ....................83
9.5.4 Patient Demographics, Baseline Characteristics and Disposition ....................83
9.5.5 Analysis of Study Treatment Dosing ....................83
9.6 Other Study Endpoints ....................84
9.6.1 Pharmacokinetic Analysis ....................84
9.6.2 Molecular Marker and Exploratory Analyses ....................84
9.7 Interim Analysis ....................84
9.8 Data Monitoring Committee ....................84
10 ETHICS AND RESPONSIBILITIES ....................84
10.1 Ethical Conduct of the Study ....................84
10.2 Obligations of Investigators ....................85
10.3 Institutional Review Board/Ethics Committee/Research Ethics Board ....................85
10.4 Informed Consent Form ....................85
10.5 Confidentiality ....................86
10.6 Reporting of Serious Breaches of the Protocol or ICH GCP ....................86
11 RECORDS MANAGEMENT ....................86
11.1 Source Documentation ....................86
11.2 Study Files and Records Retention ....................87
12 QUALITY CONTROL AND QUALITY ASSURANCE ....................87
12.1 Monitoring Procedures ....................87
12.2 Auditing and Inspection Procedures ....................87
13 CHANGES IN STUDY CONDUCT ....................88
13.1 Protocol Amendments ....................88
13.2 Protocol Deviations ....................88
14 END OF TRIAL ....................88
14.1 End of Trial in a European Union Member State ....................88
14.2 End of Trial in all other Participating Countries ....................89
14.3 Premature Termination ....................89
15 STUDY REPORT AND PUBLICATION POLICY ....................89
16 REFERENCES ....................90
APPENDIX 1. INTERNATIONAL METASTATIC RENAL CELL CARCINOMA DATABASE CONSORTIUM SCORING ....................94
APPENDIX 2. PERFORMANCE STATUS ....................95
APPENDIX 3. IMPLEMENTATION OF TITE-BOIN DOSE ESCALATION/DE-ESCALATION MODEL ....................96

APPENDIX 4. MEDICATIONS OR SUBSTANCES TO BE AVOIDED OR USED WITH CAUTION DURING TREATMENT WITH SITRAVATINIB .....103
APPENDIX 5. ABBREVIATED PRESENTATION OF RECIST VERSION 1.1 GUIDELINES .....110
APPENDIX 6. COVID-19 PANDEMIC CHANGES TO STUDY CONDUCT .....115

# TABLE OF TABLES

Table 1: Schedule of Assessments: Screening and Treatment .....11
Table 2: Schedule of PK Sample Collection and Triplicate ECGs .....14
Table 3: Schedule of Assessments: Post-Treatment .....15
Table 4: Sitravatinib Dose Escalation Levels .....47
Table 5: Nivolumab and Ipilimumab Dose Levels .....47
Table 6: Sitravatinib Sequential Dose Reductions for Individual Patients .....51
Table 7: Sitravatinib Dose Modifications: Non-Hematological Toxicities .....53
Table 8: Sitravatinib Dose Modification for Increased Blood Pressure .....55
Table 9: Sitravatinib Dose Modification for Increased Hepatic Transaminase .....59
Table 10: Laboratory Safety Parameters .....68
Table 11: Operating Characteristics of the TITE-BOIN Design .....77
Table 12: Operating Characteristics of the TOP Design .....79
Table 13: Go/No-Go Rules for Phase 1b Dose Expansion Cohorts .....79
Table 14: ECOG and Karnofsky Performance Status Comparison .....95
Table 15: TITE-BOIN Design of Dose Escalation/ De-escalation Decision Table .....97

# TABLE OF FIGURES

Figure 1: Decision Schema .....96

# LIST OF ABBREVIATIONS

| Abbreviation | Definition |
|---|---|
| AE | adverse event |
| AESI | adverse event of special interest |
| ALT | alanine aminotransferase |
| ANC | absolute neutrophil count |
| ASCO | American Society of Clinical Oncology |
| AST | aspartate aminotransferase |
| AUC | area under the plasma concentration time-curve |
| BCRP | breast cancer resistance protein |
| BP | blood pressure |
| CBR | clinical benefit rate |
| ccRCC | clear-cell renal cell carcinoma |
| CD28/80/86 | cluster of differentiation 28/80/86 |
| CFR | Code of Federal Regulations |
| CIT | checkpoint inhibitor therapy |
| $C_{max}$ | maximum plasma concentration |
| CNS | central nervous system |
| CR | complete response |
| CRC | colorectal cancer |
| CRF | case report form |
| CT | computed tomography |
| CTCAE | Common Terminology Criteria for Adverse Events |
| ctDNA | circulating tumor deoxyribonucleic acid |
| CTLA-4 | cytotoxic T lymphocyte antigen-4 |
| CYP | cytochrome P450 |
| DDR2 | discoidin domain-containing receptor 2 (CD167b) |
| DLT | dose-limiting toxicity |
| DOR | duration of response |
| EC | Ethics Committee |
| ECG | electrocardiogram |
| ECHO | echocardiogram |

| Abbreviation | Definition |
|---|---|
| ECOG | Eastern Cooperative Oncology Group |
| EIU | exposure in-utero |
| FAP | Full Analysis Population |
| FDA | Food and Drug Administration |
| FLT3 | fms-related tyrosine kinase 3 |
| FNA | fine needle aspirate |
| GCP | Good Clinical Practice |
| H2A | H2 antagonist |
| HCC | hepatocellular carcinoma |
| HDPE | high-density polyethylene |
| hERG | human Ether-à-go-go related gene |
| HIF | hypoxia-inducible factor |
| HIV | human immunodeficiency virus |
| IC50 | half maximal inhibitory concentration |
| ICF | informed consent form |
| ICH | International Council for Harmonisation |
| IFN-α/-γ | interferon-alpha/gamma |
| IgG1/G4 | immunoglobulin G1/G4 |
| IL-2 | interleukin-2 |
| ILD | interstitial lung disease |
| IMDC | International Metastatic RCC Database Consortium |
| irAE | immune-related adverse event |
| IRB | Institutional Review Board |
| IV | intravenous(ly) |
| KPS | Karnofsky Performance Status |
| LLN | lower limit of normal |
| LVEF | left ventricular ejection fraction |
| mAb | monoclonal antibody |
| MDSC | myeloid-derived suppressor cells |
| MedDRA | Medical Dictionary for Regulatory Activities |
| MRI | magnetic resonance imaging |

| Abbreviation | Definition |
|---|---|
| mRNA | messenger ribonucleic acid |
| MSI-H/dMMR | microsatellite-instability-high/mismatch repair-deficient |
| MTD | maximum tolerated dose |
| MUGA | multigated acquisition |
| NA | not applicable |
| NCI | National Cancer Institute |
| NE | not evaluable |
| NGS | next generation sequencing |
| NIVO/IPI | nivolumab and ipilimumab combination regimen |
| NIVO1/IPI3 | 1 mg/kg nivolumab plus 3 mg/kg ipilimumab combination regimen |
| NIVO3/IPI1 | 3 mg/kg nivolumab plus 1 mg/kg ipilimumab combination regimen |
| NSCLC | non-small cell lung cancer |
| NYHA | New York Heart Association |
| ORR | objective response rate |
| OS | overall survival |
| PD | progressive disease |
| PD-1 | programmed death receptor-1 |
| PDGFR | platelet-derived growth factor receptor |
| PD-L1/-L2 | programmed death-ligand 1/-ligand 2 |
| PET | positron emission tomography |
| PFS | progression-free survival |
| P-gp | P-glycoprotein |
| PK | pharmacokinetic(s) |
| PK/PD | pharmacokinetic(s)/pharmacodynamic(s) |
| PKAP | Pharmacokinetic Analysis Plan |
| PO | orally (per os) |
| PPE | Palmar-plantar erythrodysesthesia |
| PPI | proton pump inhibitor |
| PR | partial response |
| PT | Preferred Term |
| Q2W | every 2 weeks |

| Abbreviation | Definition |
|---|---|
| Q3W | every 3 weeks |
| Q4W | every 4 weeks |
| QD | once daily |
| RCC | renal cell carcinoma |
| REB | Research Ethics Board |
| RECIST | Response Evaluation Criteria in Solid Tumors |
| RTK | receptor tyrosine kinase |
| SAE | serious adverse event |
| SAP | Statistical Analysis Plan |
| SAR | serious adverse reaction |
| SD | stable disease |
| SOC | System Organ Class |
| SUSAR | serious unexpected serious adverse reaction |
| TAM | TYRO3, Axl, and MER |
| TEAE | treatment-emergent adverse event |
| TKI | tyrosine kinase inhibitor |
| TME | tumor microenvironment |
| Treg | regulatory T-cell |
| TSH | thyroid-stimulating hormone |
| UC | urothelial cancer |
| ULN | upper limit of normal |
| USPI | United States Prescribing Information |
| VEGF | vascular endothelial growth factor |
| VEGFR | vascular endothelial growth factor receptor |
| VHL | von Hippel-Lindau |
| WHO | World Health Organization |
| WOCBP | women of child-bearing potential |

# 1 INTRODUCTION AND STUDY RATIONALE

## 1.1 Cancer Immunotherapy

Cancer immunotherapy comprises therapies that stimulate the immune system to recognize tumors as foreign rather than self and eradicate the tumor. An effective immune response relies on immune surveillance of tumor antigens expressed on cancer cells that are recognized and ultimately killed by the adaptive immune response. Tumor progression likely depends on the tumor acquiring traits that allow it to evade recognition or destruction by the immune system, including the co-opting of immune checkpoint pathways that block activation of key immune cell types. Therapeutic antibodies as well as small molecule inhibitors have been developed to target immune checkpoint pathways and key targets that regulate the critical interactions between cancer cells and various immune cell types. Several of these therapeutic approaches, as single agents and in combination, are proving to be capable of instructing the adaptive immune system to attack tumors and are approved for the treatment of several cancers.

Tumors can avoid immune surveillance by stimulating immune inhibitory receptors that function to turn off established immune responses. By blocking the ability of tumors to stimulate inhibitory receptors on T cells, sustained, antitumor immune responses can be generated in animals. Thus, therapeutic blockade of immune inhibitory checkpoints provides a potential method to boost antitumor immunity.

### *1.1.1 Checkpoint Pathway Inhibition*

#### *1.1.1.1 Programmed Death Receptor-1 (PD-1) and Inhibition by Nivolumab*

Programmed death receptor-1 (PD-1), a 55 kDa type I transmembrane protein, is a member of the CD28 family of T-cell receptors. Programmed death-ligand 1 (PD-L1) and programmed death-ligand 2 (PD-L2) downregulate T-cell activation upon binding to PD-1, thereby creating an important immune checkpoint pathway (Mellman-2011; Topalian-2015). PD-1 is expressed on T cells, and its ligands PD-L1 and PD-L2 are expressed on some immune cell types as well as cancer cells. Upregulation of PD-1 ligands is utilized by some tumors to help evade detection and elimination by the host immune system tumor response. PD-L1 is the predominant ligand expressed in solid tumors and is upregulated by interferon gamma (IFN-γ). PD-L1 functions to limit collateral damage in normal tissues where an immune response has been triggered. Its expression in tumors, including in lung cancer, has been associated with poor survival (Mu-2011), motivating the development of PD-1 pathway inhibitors.

One such inhibitor is nivolumab, a fully human immunoglobulin G4 (IgG4) monoclonal antibody (mAb). Nivolumab binds to PD-1 and selectively blocks its interaction with PD-L1 and PD-L2, thereby releasing PD-1-mediated inhibition of the immune response, including the antitumor immune response. In syngeneic mouse tumor models, blocking PD-1 activity resulted in decreased tumor growth (Johnson-2015). Nivolumab was first

approved in 2014 by the United States (US) Food and Drug Administration (FDA) for advanced melanoma (Robert-2015). Nivolumab is also indicated for the treatment of several cancer types as monotherapy and in combination with other anticancer therapies, including ipilimumab. Additional background information is available in the United States Prescribing Information (USPI) for OPDIVO® (nivolumab).

#### *1.1.1.2 Cytotoxic T-Lymphocyte Antigen 4 (CTLA-4) and Inhibition by Ipilimumab*

Another inhibitory immune checkpoint protein targeted for its antitumor potential is cytotoxic T-lymphocyte antigen-4 (CTLA-4), a type I transmembrane protein in the CD28 receptor family. CTLA-4 is exclusively expressed in T cells and modulates the early stages of T cell activation. CTLA-4 turns off T cell activation, in part, by outcompeting CD28 for binding of the two ligands CD80 (B7.1) and CD86 (B7.2), thereby decreasing CD28-mediated stimulation of T cells (Alegre-1996; Postow-2012). CTLA-4 also dampens T cell activation directly by delivering inhibitory signals within the cell (Pardoll-2012). CTLA-4 is constitutively expressed in regulatory T cells and is upregulated in activated T cells, a phenomenon that is particularly notable in cancer cells. Increased CTLA-4 expression has been demonstrated for some cancer cell types in vitro and in vivo, and certain polymorphisms may be associated with increased cancer susceptibility (Yan-2013). Blocking CTLA-4 function in mouse xenograft studies resulted in decreased tumor growth, seemingly "releasing" the immune system to attack injected tumor cells and pre-established tumors (Leach-1996).

The first anticancer therapy developed to target an immune checkpoint protein was ipilimumab, a fully human IgG1 mAb that binds to CTLA-4 and interferes with its binding of CD80 and CD86. Ipilimumab was first approved by the US FDA in 2011 for the treatment of advanced melanoma (Hodi-2010) and has since been approved for use in several malignancies as a monotherapy and in combination with other anticancer therapies including nivolumab. Additional background information is available in the YERVOY® (ipilimumab) USPI.

#### *1.1.1.3 Dual Checkpoint Inhibition: Combining Nivolumab and Ipilimumab*

As negative regulators of T-cell activation, PD-1 and CTLA-4 both play important, nonredundant roles in the co-inhibitory mechanisms of immune responses. CTLA-4 limits T-cell activation and clonal expansion, whereas PD-1 primarily inhibits effector T-cell function in tissues. Both proteins have been targeted clinically for their potential role in dysregulation of tumor immunogenicity, and the combination of PD-1 and CTLA-4 blockade has been investigated as a way to increase antitumor activity compared to inhibition of either protein alone. Preclinical data have shown that the combined activity of nivolumab (anti-PD-1) and ipilimumab (anti-CTLA-4) leads to enhanced T-cell function in a mixed lymphocyte reaction and increased antitumor activity in several syngeneic mouse systems compared with the effects of either antibody alone (OPDIVO USPI-2019).

The combination of nivolumab and ipilimumab (NIVO/IPI) was first approved by the US FDA in 2015 for first-line treatment of melanoma, with improved response rates for the combination regimen compared with either agent alone. NIVO/IPI treatment is also approved for first-line treatment of intermediate/poor-risk clear-cell renal cell carcinoma (ccRCC) and for second-line treatment of microsatellite-instability-high/mismatch repair-deficient (MSI-H/dMMR) colorectal cancer (CRC) following treatment with fluoropyrimidine, oxaliplatin, and irinotecan (OPDIVO USPI-2019). Favorable activity in several other cancer types has been observed with NIVO/IPI treatment as well, including favorable-risk RCC (Hammers-2017), advanced hepatocellular carcinoma (HCC) (Yau-2019), metastatic urothelial cancer (UC) (Sharma-2019), and non-small cell lung cancer (NSCLC) (Hellmann-2017).

### *1.1.2 Tyrosine Kinase Inhibition*

The use of tyrosine kinase inhibitors (TKIs) to treat cancer is well established based on robust clinical efficacy achieved with well tolerated inhibitors directed toward oncogenic tyrosine kinases. Select TKIs have been shown to modulate the immunogenic status of tumors, improve tumor perfusion by reducing intratumoral pressure and modulate subsets of immune cells, thereby increasing the frequency and function of effector immune elements while decreasing the number and function of immune suppressor cells.

#### *1.1.2.1 Tyrosine Kinase Inhibition by Sitravatinib*

Sitravatinib is a spectrum-selective receptor tyrosine kinase (RTK) inhibitor that inhibits several closely related RTKs including the TAM family (Tyro3/Axl/MERTK), VEGFR2, KIT, and MET. Receptor tyrosine kinases are key regulators of signaling pathways leading to cell growth, survival, and migration (Blume-Jensen-2001). These kinases are dysregulated in many cancers through overexpression, genetic alteration or co-expression with high affinity ligands (Blume-Jensen-2001). Multiple sitravatinib RTK targets are genetically altered in a variety of cancers and act as oncogenic drivers, promoting cancer development and progression. In addition to the immunostimulatory effects of Axl and MET inhibition, sitravatinib may further condition the tumor microenvironment (TME) in favor of antitumor activity by its immunomodulatory effects mediated through VEGFR and KIT inhibition.

#### *1.1.2.2 Previous Experience with Sitravatinib*

Sitravatinib is under development for the treatment of several solid malignancies as a single agent and in combination with checkpoint inhibitor therapies (CITs). For background information in addition to that presented below, refer to the Sitravatinib (MGCD516) Investigator’s Brochure.

##### 1.1.2.2.1 <u>Drug Substance</u>

The chemical structure and chemical formula of sitravatinib (MGCD516) malate is as follows:

**MGCD516 L-Malate**

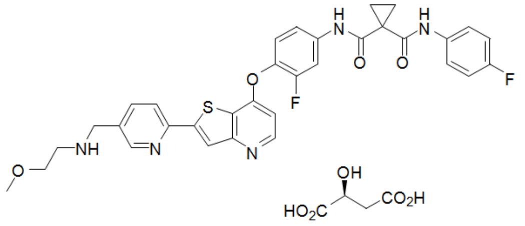

**Chemical Formula:**

MGCD516 Malate: $C_{37}H_{35}F_2N_5O_9S$

**Molecular Weight:**

MGCD516 Malate: 763.76

#### 1.1.2.2.2 Nonclinical Data

Sitravatinib demonstrated potent, concentration-dependent inhibition of the kinase activity of MET, Axl, MERTK, VEGFR family, PDGFR family, KIT, FLT3, Trk family, RET, DDR2, and selected Eph family members in biochemical assays and inhibited phosphorylation and kinase dependent function in cell-based assays. Sitravatinib also inhibited oncogenic functions associated with target RTKs including MET-dependent cell viability and migration and endothelial tube formation and angiogenesis. Consistent with this antitumor and anti-angiogenic mechanism of action, sitravatinib demonstrated antitumor efficacy over a broad spectrum of human tumor xenograft models including robust cytoreductive antitumor activity in a subset of models exhibiting genetic alterations in RTK targets including MET, RET, FLT3, and others.

In vitro results from the hERG (human Ether-à-go-go related gene) assay demonstrate a half maximal inhibitory concentration (IC50) of 0.6 μM on the potassium current, which far exceeds exposures observed clinically. There were no adverse effects on the cardiovascular system, including no effect on the QTc interval, when sitravatinib was administered to dogs at doses up to 4 mg/kg (mean 6-hour concentration of 0.072 μg/mL). Minor increases in vascular pressures were observed during the dog cardiovascular study; however, these were mild and considered of limited biological consequence. Assessment of the neurological functional observation battery and respiratory evaluations (tidal volume, respiration rate, and minute volume) in rats did not reveal any sitravatinib-related effects at doses up to 25 mg/kg.

In a bidirectional permeability study with Caco-2 cell lines, sitravatinib is classified as a highly permeable compound, and not a substrate of P-glycoprotein (P-gp) and breast cancer resistance protein (BCRP). A P-gp and BCRP inhibition study using Caco-2 cells

suggested that MGCD516 is a significant inhibitor of P-gp and BCRP with IC50 value of 0.838 and 1.51 μM (528 and 951 μg/mL), respectively; these values are much higher than the systemic steady state exposure levels observed clinically ($C_{trough}$ of 68 ng/mL).

Using an ultra-centrifugation technique sitravatinib was 98.6% bound to human plasma proteins.

Sitravatinib (MGCD516) was evaluated for cytochrome P450 (CYP)-mediated metabolism using human liver microsomes and recombinant human enzymes. Results suggest that multiple enzymes, including CYP 1A2, 2A6, 2B6, 2C8, 2C9, 2D6, 2E1, and 3A4 are involved in the metabolism of sitravatinib.

The effect of treating primary cultures of cryopreserved human hepatocytes with MGCD516 on the expression of CYP enzymes was investigated. Treatment of cultured human hepatocytes with up to 30 µM MGCD516 caused little or no increase (<2-fold change or <20% of the positive control) in CYP1A2 activity, CYP1A2 messenger ribonucleic acid (mRNA) levels, or CYP3A4 activity. However, MGCD516 (up to 3 and 10 µM, 1889 and 6297 ng/mL) caused concentration dependent increases (>2-fold change and >20% of the positive control) in CYP2B6 activity, CYP2B6 mRNA levels, and CYP3A4 mRNA levels in one or more human hepatocyte cultures. Sitravatinib is not expected to act as a CYP enzyme inducer at the concentration levels observed clinically.

There was little or no evidence of direct inhibition of CYP1A2, CYP2A6 or CYP2E1 by MGCD516 or time- or metabolism-dependent inhibition of any of the CYP enzymes evaluated. Under the experimental conditions examined, MGCD516 demonstrated direct inhibition of CYP2C8, CYP2C9, CYP2C19, CYP2D6 and CYP3A4/5 (as measured by testosterone 6β-hydoxylation and midazolam 1´-hydroxylation) with IC50 values of 2.9, 11, 10, 1.9, 11, and 0.81 μM, respectively. These IC50 values are higher than observed MGCD516 concentration levels using the recommended sitravatinib dose of 120 mg once daily (QD). It is therefore unlikely that inhibition of CYP enzymes tested will be observed during clinical use of sitravatinib.

Because the potency for MGCD516 against its intended clinical targets is generally less than 0.1 μM, it may be unlikely that concentrations required for robust direct systemic inhibition/induction of the tested CYPs will be achieved at projected clinical dose and exposure levels.

For additional nonclinical information, refer to the current sitravatinib Investigator’s Brochure.

#### 1.1.2.2.3 Sitravatinib Clinical Experience

Sitravatinib has been administered to cancer patients in multiple clinical studies, including monotherapy studies (516-001 and BGB-900-104); combination studies with the PD-1 inhibitor nivolumab (MRTX-500, 516-002, 516-003, and 2016-0332); and combination studies with the PD-1 inhibitor tislelizumab (BGB-900-103 and

BGB-900-104). Sitravatinib has also been administered as a single agent in healthy subject studies (516-006 and 516-007).

###### *1.1.2.2.3.1 Sitravatinib Pharmacokinetics*

Complete information concerning sitravatinib pharmacokinetics (PK) is available in the Investigator's Brochure. Investigations of alternative formulations were performed for the purpose of optimizing product characteristics and manufacturing efficiency.

PK evaluation in patients with advanced solid tumor malignancies in Study 516-001 showed that after single-dose administration of sitravatinib free base capsules, sitravatinib reached peak concentration in a median time of approximately 3 to 8 hours. Exposure parameters (maximum plasma concentration [$C_{max}$] and area under the plasma concentration-time cure [AUC]) were approximately dose proportional with single doses up to 200 mg. Median elimination half-life varied between 43 and 58 hours, and the steady-state PK exposure was reached in a mean time of 11 to 15 days. Based on long-term tolerability data, the recommended Phase 2 dose (RP2D) was 120 mg QD sitravatinib free base.

Study 516-006 evaluated the relative bioavailability and PK of sitravatinib free base and malate salt capsule formulations following oral administration in healthy subjects. Following a single oral dose administration, sitravatinib free base and malate capsule formulations exhibited similar PK profiles with a median $T_{max}$ of approximately 8 hours and arithmetic mean elimination half-life of approximately 34 to 35 hours.
Part 1 of the study demonstrated that the bioavailability of sitravatinib for the 80 mg sitravatinib malate capsule formulation is approximately 30% higher than for the 80 mg sitravatinib free base capsule formulation based on sitravatinib primary PK parameters $AUC_{0-\infty}$, $AUC_{0-t}$, and $C_{max}$.

Part 2 of the study further demonstrated that 100 mg sitravatinib malate capsule formulation is bioequivalent to 120 mg sitravatinib free base capsule formulation based on the primary PK parameters ($AUC_{0-\infty}$, $AUC_{0-t}$, and $C_{max}$). Thus, the 100 mg malate capsule formulation administered once daily is expected to achieve similar exposure to the 120 mg freebase capsule formulation previously evaluated in Studies 516-001 and MRTX500.

###### *1.1.2.2.3.2 Sitravatinib Clinical Safety*

As of 26 June 2019, safety data are available for a total of 422 patients treated with sitravatinib, either as a single agent (n = 189), in combination with the PD-1 inhibitor nivolumab (n = 184), or in combination with tislelizumab (n = 49). In addition, safety data are available for 16 healthy male subjects administered single-agent sitravatinib.

Dose-limiting toxicities (DLTs) with single-agent administration of sitravatinib in Study 516-001 included Grade 3 palmar-plantar erythrodysesthesia (PPE) at 80 mg free base, and intolerable Grade 2 neuropathy, intolerable Grade 2 fatigue, and intolerable

Grade 2 mucositis at 200 mg free base. In Study MRTX-500, there were no DLTs observed during the lead-in evaluation of the combination of nivolumab (240 mg IV every 2 weeks [Q2W]) and sitravatinib (120 mg QD free base).

Sitravatinib-related adverse events (AEs) reported in ≥20% of 186 patients treated with sitravatinib monotherapy were diarrhea (50%), fatigue (42%), hypertension (39%), nausea (29%), decreased appetite (27%), vomiting (24%), and PPE syndrome (20%). Treatment-related Grade 3+ AEs reported in ≥5% of patients were hypertension (19%), diarrhea (10%), fatigue (7%), lipase increased (5%), and PPE (5%). Treatment-related Grade 4 AEs were reported in 3 patients and included lipase increased in 2 patients (1%) and febrile neutropenia in 1 patient (1%). A treatment-related Grade 5 AE of cardiac arrest was reported in 1 patient (1%).

Treatment-related AEs reported in ≥20% of 184 patients treated with sitravatinib in combination with nivolumab were diarrhea (50%), fatigue (46%), nausea (34%), decreased appetite (33%), hypertension (27%), weight decreased (26%), dysphonia (24%), vomiting (23%), and hypothyroidism (20%). Treatment-related Grade 3 AEs reported in ≥5% of patients were hypertension (19%), diarrhea (10%), fatigue (7%), and lipase increased (5%). Treatment-related Grade 4 AEs were reported in 4 patients (2%) overall, and included gastric ulcer perforation, hypertensive crisis, lipase increased, and lymphocyte count decreased in 1 patient each (1%). Treatment-related Grade 5 AEs of cardiac arrest were reported in 2 patients (1%).

Based on review of the AEs reported with sitravatinib in context of the mechanism of action, nonclinical data, frequency, and Investigator assessment of causality, the following AEs have been assessed as expected serious adverse reactions (SARs) for sitravatinib, or serious adverse events (SAEs) with at least a reasonable possibility of a causal relationship to sitravatinib administered as monotherapy or in combination with other agents: deep vein thrombosis/embolism/pulmonary embolism, diarrhea, ejection fraction decreased, fatigue, hypertension, nausea, PPE syndrome, and vomiting.

###### *1.1.2.2.3.3 Sitravatinib Clinical Efficacy*

Preliminary evidence of clinical activity with sitravatinib in combination with CITs has been observed in NSCLC, RCC, and UC. Early correlative biomarker and response data support the proposed immunomodulating mechanism of action of sitravatinib, with the potential to reawaken an adaptive immune response and improve response rates and the durability of responses to CIT. Refer to Section 1.2.2.2 for additional details.

## 1.2 Study Rationale

### *1.2.1 Rationale for Combining Nivolumab, Ipilimumab, and Sitravatinib*

The current study is designed to evaluate the safety and antitumor activity of a novel, triple-drug combination of sitravatinib and the well-established combination regimen of CITs nivolumab and ipilimumab (NIVO/IPI). Combining sitravatinib and NIVO/IPI is

predicted to have complementary effects in triggering a tumor-directed immune response. In addition to the mechanistic rationale described herein, previous and ongoing studies that inform the current study design include those investigating NIVO/IPI regimens (described in Sections 1.1.1.3 and 1.2.2.1) and the combination of sitravatinib and CITs, including nivolumab (described in Sections 1.1.2.2.3 and 1.2.2.2).

As a spectrum-selective TKI, sitravatinib inhibits several closely related RTKs, including the TAM family, VEGFR2, KIT, and MET. Based on the role of these receptors in key immune cell types, sitravatinib and CITs are predicted to have complementary effects in triggering a tumor-directed immune response. Moreover, VEGF/VEGFR2-targeting therapies are used for the treatment of several cancers as a single agent or in combination with a broad range of cancer therapeutics, including checkpoint inhibitors.

The mechanistic rationale supporting the combination of PD-1 inhibition by nivolumab and CTLA-4 inhibition by ipilimumab is distinct and largely non-overlapping with the rationale supporting the combination of PD-1 inhibition by nivolumab plus RTK inhibition by sitravatinib. CTLA-4 is a checkpoint expressed on T-cell populations, whereas sitravatinib reverses the immunosuppressive function of several innate immune cell types, including macrophages, dendritic cells and myeloid-derived suppressor cells (MDSCs), in addition to regulatory T-cell (Tregs). Given the distinct immune suppressive mechanisms targeted by CTLA-4 antibodies and sitravatinib, coupled with the observation that both agents effectively augment PD-1 inhibition, the triple combination of nivolumab, sitravatinib, and ipilimumab is a rational strategy to treat several cancer types in which checkpoint inhibitors are clinically active, but only in a limited number of patients.

#### *1.2.1.1 Clinical Studies Combining Sitravatinib with Checkpoint Inhibitors*

The combination of sitravatinib and a PD-1 inhibitor (nivolumab or tislelizumab) is being evaluated in multiple types of cancers in patients who have progressed on or were refractory to single-agent PD-1 pathway inhibitors. Preliminary clinical activity has been observed in NSCLC, RCC, and UC. The combination of sitravatinib plus nivolumab is also being tested in the checkpoint inhibitor naïve setting based on the rationale and the activity observed in the checkpoint inhibitor-refractory setting. Refer to Section 1.2.2.2 for preliminary findings of clinical activity in RCC. Overall, preliminary safety data suggest that sitravatinib in combination with nivolumab is tolerable.

Although no data exist for the combination of sitravatinib with NIVO/IPI, there is evidence of activity for the combination of a sitravatinib-like drug, cabozantinib (CABOMETYX®), with NIVO/IPI. The TKI cabozantinib has a similar target profile compared to sitravatinib in that both inhibit VEGFR2 and the TAM receptors. Cabozantinib has been explored in a Phase 1 study in combination with NIVO/IPI in several cancer types. Based on the initial safety and clinical activity data from this Phase 1 trial, a large, randomized Phase 3 study has been initiated in RCC (COSMIC-313) exploring this triple combination therapy. This provides some

evidence that the combination of nivolumab, ipilimumab, and a spectrum-selective, VEGFR2/TAM-targeting TKI may be safe and effective for the treatment of select cancer types.

### *1.2.1.2 Expectations for Safety of the Combination of Nivolumab, Ipilimumab, and Sitravatinib*

#### 1.2.1.2.1 **Potential for Drug-Drug Interactions**

Sitravatinib administered in combination with nivolumab and ipilimumab is unlikely to result in clinically relevant drug-drug interactions based on absorption, metabolism, elimination or protein binding. Nivolumab and ipilimumab are mAbs that are intravenously administered, whereas sitravatinib is a small molecule therapeutic administered orally; no absorption interactions are expected.

No studies on the metabolism of nivolumab or ipilimumab have been reported in in vitro or in human studies. Like most mAbs, nivolumab and ipilimumab are not expected to be metabolized by liver CYP or other drug-metabolizing enzymes and are unlikely to have an effect on CYPs or other metabolizing enzymes in terms of inhibition or induction.

#### 1.2.1.2.2 Evaluation of Potential for Increased Toxicities with Combination Use of Sitravatinib, Nivolumab, and Ipilimumab

Frequent AEs, such as fatigue, decreased appetite, nausea and diarrhea, which are non-specific and typical of cancer treatment regimens have been observed with nivolumab, ipilimumab, and sitravatinib monotherapy. Potential exists for these AEs to be observed with increased severity or frequency during use of the agents in combination. Management of these effects in patients receiving cancer therapy is well precedented.

Importantly, immune-related adverse events (irAEs) based on observed safety events using nivolumab and/or ipilimumab monotherapy include pneumonitis, enterocolitis/colitis, hepatitis, endocrinopathy, nephritis/renal dysfunction, rash/dermatitis, encephalitis, and neuropathy. While sitravatinib may have immunostimulatory effects, significant autoimmune AEs have not been recognized as class effects for this agent. The potential for sitravatinib to exacerbate or promote irAEs when administered in combination with checkpoint inhibitors should be borne in mind. AE incidence data presented herein are as reported in the OPDIVO® (nivolumab) USPI dated September 2019, YERVOY® (ipilimumab) USPI dated May 2019, and the Sitravatinib (MGCD516) Investigator’s Brochure, with sitravatinib cumulative safety data as of 26 June 2019. Updates to these data during the conduct of this clinical trial will be found in the current USPIs for OPDIVO® and YERVOY®, and the current Sitravatinib (MGCD516) Investigator’s Brochure.

###### 1.2.1.2.2.1 Immune-Related Enterocolitis/Colitis

A clinically relevant overlap in toxicity may arise between the immune-related enterocolitis/colitis attributed to NIVO/IPI and the non-specific, most often mild to moderate diarrhea observed with sitravatinib.

Immune-mediated colitis occurred in 10% (52/547) of patients with RCC and 7% (8/119) of patients with CRC who received the NIVO3/IPI1 regimen (nivolumab 3 mg/kg plus ipilimumab 1 mg/kg every 3 weeks [Q3W]). Median time to onset of immune-mediated colitis was 1.7 months (range: 2 days to 19.2 months) in patients with RCC and 2.4 months (range: 22 days to 5.2 months) in patients with metastatic CRC (OPDIVO USPI-2019; YERVOY USPI-2019).

Sitravatinib-related diarrhea has been reported in approximately 46% (192/422) of all patients treated with sitravatinib monotherapy or in combination with CITs, most often beginning within the first month of the start of treatment. Of the 223 patients receiving sitravatinib in combination with a checkpoint inhibitor, immune-related diarrhea was reported for 7 patients (3%). Diarrhea (any grade) is common with NIVO/IPI, occurring in 38% of patients with RCC (OPDIVO USPI-2019; YERVOY USPI-2019). The time to onset may be helpful in distinguishing diarrhea that may be attributed to autoimmune effects versus non-specific toxicity.

###### 1.2.1.2.2.2 Immune-Related Hepatitis

A clinically relevant overlap in toxicity may arise between the immune-related hepatitis attributed to NIVO/IPI and the non-specific, most often mild to moderate elevation in aspartate transaminase (AST) and alanine transaminase (ALT) observed with TKIs in general, and MET inhibitors in particular. Sitravatinib-related changes in transaminases were observed for ALT increased in 19% (78/422) and AST increased in 18% (75/422) of all patients treated with sitravatinib monotherapy or in combination with CITs. The elevations observed with sitravatinib generally occur within the first 28 days of treatment and resolve with interruption of treatment. Of the 223 patients receiving sitravatinib in combination with a checkpoint inhibitor, immune-related transaminase changes included ALT increased in 7 patients (3%), AST increased in 6 patients (3%), and transaminase increased in 2 patients (1%).

Immune-mediated hepatitis has been observed in 7% (38/547) of patients with RCC and 8% (10/119) with CRC receiving the NIVO3/IPI1 regimen. Median time to onset was 2 months (range: 14 days to 26.8 months) in patients with RCC and 2.2 months (range: 22 days to 10.5 months) in patients with CRC (OPDIVO USPI-2019; YERVOY USPI-2019).

###### 1.2.1.2.2.3 Autoimmune Thyroid Disorders

A clinically relevant overlap in toxicity may arise between the autoimmune thyroid disorders observed with treatment with NIVO/IPI or with sitravatinib. Sitravatinib-related

hypothyroidism has been reported in approximately 12% (50/422) of all patients treated with sitravatinib. Of the 223 patients receiving sitravatinib in combination with a checkpoint inhibitor, immune-related thyroid events included hypothyroidism in 24 patients (10%), hyperthyroidism in 9 patients (4%), and blood thyroid-stimulating hormone increased and thyroiditis in 4 patients each (2%).

Hypothyroidism or thyroiditis resulting in hypothyroidism occurred in 22% (119/547) of patients with RCC and 15% (18/119) of patients with CRC who received the NIVO3/IPI1 regimen. Median time to onset was 2.2 months (range: 1 day to 21.4 months) in patients with RCC and 2.3 months (range: 22 days to 9.8 months) in patients with CRC. Hyperthyroidism occurred in 12% (66/547) of patients with RCC and 12% (14/119) of patients with CRC who received the NIVO3/IPI1 regimen. Median time to onset was 1.4 months (range: 6 days to 14.2 months) in RCC and 1.1 months (range: 21 days to 5.4 months) in CRC (OPDIVO USPI-2019; YERVOY USPI-2019).

###### *1.2.1.2.2.4 Immune-Related Rash/Dermatitis*

A clinically relevant overlap in toxicity may arise between the immune-related rash/dermatitis attributed to NIVO/IPI treatment and the non-specific, most often mild (Grade 1) rash observed with sitravatinib. Immune-related rash occurred in 16% (90/547) of patients with RCC and 14% (17/119) of patients with CRC who received NIVO3/IPI1. Median time to onset was 1.5 months (range: 1 day to 20.9 months) in RCC and 26 days (range: 5 days to 9.8 months) in CRC (OPDIVO USPI-2019; YERVOY USPI-2019). Sitravatinib-related events of rash have been reported in 8% (34/422) and rash maculopapular in 5% (22/422) of all patients treated with sitravatinib. Of the 223 patients receiving sitravatinib in combination with a checkpoint inhibitor, immune-related skin reactions included rash maculopapular in 5 patients (2%), rash in 3 patients (1%), and dermatitis acneiform and pruritus in 2 patients each (1%).

### 1.2.2 Rationale for Treatment in Renal Cell Carcinoma

RCC that originates within the renal cortex is responsible for 80% to 85% of all primary renal neoplasms (Garfield-2018). In the United States, kidney cancer (including RCC) is the eighth most common cancer for men and the ninth most common cancer for women (ACS-2020). An estimated 73,750 new cases of kidney and renal pelvis cancer and 14,830 deaths are expected in 2020 (ACS-2020). Incidence rates of RCC have been rising over the past 50 years, with most of the increases occurring in early-stage tumors (Garfield-2018). Incidence rates of RCC have been rising over the past 50 years, with most of the increases occurring in early-stage tumors (Garfield-2018). Clear-cell RCC (ccRCC) is the most common RCC histologic type, representing between 70% and 85% of all RCCs, and is overwhelmingly a sporadic (nonhereditary) cancer (Muglia-2015; Escudier-2014).

ccRCC is characterized by two important features. First, approximately 50% of sporadic ccRCC is associated with inactivating mutations of the von Hippel-Lindau (VHL) tumor

suppressor gene, which leads to upregulation of hypoxia-inducible factors (HIFs) and to downstream upregulation of VEGF, resulting in increased angiogenesis (Rini-2005; Cohen-2012; Fischer-2015). VHL inactivation also leads to overexpression of RTKs MET and AXL, which have been implicated in RCC pathogenesis, prognosis, and resistance to standard anti-angiogenic therapies (Tannir-2017). Second, ccRCC is often susceptible to immune regulation (Cho-2017). These two features have led to the use of several active agents that either target the VEGF receptor (VEGFR) pathway (eg, sunitinib, pazopanib) or modulate immune response (eg, interferon-alpha [IFN-α], interleukin-2 [IL-2], nivolumab) (Rini-2016).

#### *1.2.2.1 NIVO/IPI First-Line Treatment of Patients with ccRCC*

Treatment options in the first-line setting for advanced and metastatic ccRCC have evolved rapidly with the development of novel therapies targeting VEGF and immune pathways. Recent developments have included the approval of combinations of VEGF inhibitors with anti-PD-1 CITs in ccRCC, dual checkpoint blockade with nivolumab plus ipilimumab, particularly as a preferred treatment option for poor/intermediate risk ccRCC (NCCN-2019).

In an open-label, multicenter, Phase 3 study in advanced ccRCC (CheckMate 214), patients received either the NIVO3/IPI1 regimen (nivolumab 3 mg/kg plus ipilimumab 1 mg/kg IV Q3W for 4 doses followed by nivolumab 3 mg/kg Q2W) or standard of care with sunitinib monotherapy 50 mg (4 weeks on and 2 weeks off schedule). Patients with poor/intermediate risk ccRCC who received the NIVO3/IPI1 combination (n = 425) experienced a higher overall response rate (ORR) (42% vs. 27%), higher complete response (CR) rate (9% vs. 1%), and higher 18-month overall survival (OS) rate (75% vs. 60%) compared to those receiving sunitinib (n = 422) (Motzer-2018). Results were mixed for patients with favorable risk ccRCC, with lower ORR (29% and 52%) and lower 18-month OS rates (88% vs. 93%) for the NIVO3/IPI1 group (n = 125) versus the sunitinib group (n = 124); however, CR rates favored NIVO3/IPI1 treatment (11% vs. 6%) (Motzer-2018).

Results from a separate Phase 1 study (CheckMate 016) support the use of NIVO/IPI combination treatment in ccRCC for all risk groups and for treatment-naïve and previously treated patients (Hammers-2017). Patients in this study received either the NIVO3/IPI1 or the NIVO1/IPI3 regimen (nivolumab 1 mg/kg plus ipilimumab 3 mg/kg IV Q3W for 4 doses followed by nivolumab 3 mg/kg Q2W), with favorable-risk patients comprising 47% of the total population (N = 100). Although data for the favorable risk patients alone were not published, results for the total population included 2-year OS of 67.3% and 69.6% for NIVO3/IPI1 and NIVO1/IPI3, respectively, and the confirmed ORR was the same in both arms (40.4%) (Hammers-2017).

Based on these data, the NCCN Kidney Cancer Panel has listed NIVO/IPI as a category 1, preferred treatment option for first-line treatment for intermediate- and poor-risk patients with previously untreated advanced ccRCC, and category 2A other

recommended treatment option for first-line treatment in favorable risk ccRCC. The US FDA approval for nivolumab plus ipilimumab is narrower, only including patients with intermediate- or poor-risk ccRCC (NCCN-2019).

#### *1.2.2.2 Sitravatinib Treatment of Patients with ccRCC*

Ongoing clinical studies have observed favorable activity with sitravatinib treatment in the neoadjuvant and metastatic RCC settings. In Study 516-002, patients receive neoadjuvant sitravatinib alone for 2 weeks followed by sitravatinib plus nivolumab for 4 to 6 weeks prior to planned nephrectomy. Preliminary data are suggestive of clinical activity, with correlative biomarker data supporting the reversal of immunosuppressive cells in the TME in responders.

In Study 516-001, sitravatinib monotherapy in the metastatic setting, particularly in later line VEGF-refractory setting, is under evaluation in an RCC expansion cohort. As of 24 July 2019, a total of 8 confirmed responses out of 32 evaluable patients (25% ORR) has been observed. This is comparable to response rates with other single-agent TKIs in RCC, such as axitinib (19% ORR) (Rini-2012; Motzer-2013) and cabozantinib (21% ORR) (Choueiri-2015), and with single-agent checkpoint protein inhibitors, such as nivolumab (25% ORR) (Motzer-2015a).

In the Investigator-initiated study 2016-0332, the combination of sitravatinib and nivolumab is being evaluated in the VEGF-refractory RCC setting. As of 29 August 2019, a total of 13 responses out of 33 patients (39% ORR) has been observed, which is comparable to other combination treatments in RCC, such as NIVO/IPI (40% ORR [Hammers-2017]) and lenvatinib plus everolimus (40% ORR [Motzer-2015b; Motzer-2016]).

Based on these findings, the current study is designed to evaluate the triple combination of sitravatinib plus NIVO/IPI in patients with solid tumor malignancies that have shown favorable responses to NIVO/IPI combinations in previous clinical trials. Initially the study will evaluate sitravatinib plus NIVO3/IPI1 as first-line therapy in patients with intermediate/poor-risk advanced or metastatic ccRCC. The NIVO3/IPI1 regimen is approved for treatment of this patient population. Sitravatinib dose escalation cohorts begin at 35 mg QD, a dose well below the RP2D of sitravatinib in combination with nivolumab.

# 2 STUDY OBJECTIVES AND ENDPOINTS

### *2.1.1 Objectives*

#### *2.1.1.1 Primary Objectives*

- To evaluate the safety and tolerability of sitravatinib in combination with nivolumab and ipilimumab in the study populations.

#### *2.1.1.2 Secondary Objectives*

- To evaluate the clinical activity of sitravatinib in combination with nivolumab and ipilimumab.
- To evaluate the PK of sitravatinib when administered in combination with nivolumab and ipilimumab.

#### *2.1.1.3 Exploratory Objectives*

- To assess the effects of the combination regimen on tumor cell PD-L1 expression, tumor infiltrating immune cell populations, and gene expression signatures.
- To assess correlations between treatment-related outcomes and tumor immune biomarkers and tumor gene mutations.

### *2.1.2 Endpoints*

#### *2.1.2.1 Primary Endpoint*

- Safety characterized by type, incidence, severity, timing, seriousness, and relationship to study treatment of AEs, and laboratory abnormalities.

#### *2.1.2.2 Secondary Endpoints*

- Clinical Activity Endpoints:
  - Objective response rate (ORR) as defined by Response Evaluation Criteria in Solid Tumors version 1.1 (RECIST 1.1);
  - Duration of response (DOR);
  - Clinical benefit rate (CBR);
  - Progression-free survival (PFS);
  - One-year survival rate; and

    - Overall survival (OS).

- Blood plasma concentrations of sitravatinib.

#### *2.1.2.3 Exploratory Endpoints*

- Tumor PD-L1 expression;
- Immune cell populations in the tumor;
- Gene expression signatures in the tumor;
- Tumor gene alterations; and
- Circulating tumor deoxyribonucleic acid (ctDNA).

# 3 STUDY DESIGN

Study 516-008 is an open-label Phase 1 dose escalation/Phase 1b dose expansion study evaluating the safety and tolerability, clinical activity, and PK of sitravatinib in combination with nivolumab and ipilimumab for the treatment of ccRCC and potentially other solid tumor types. The Schedule of Assessments is provided in Table 1.

Initially, Phase 1 dose escalation cohorts will evaluate sitravatinib administered in combination with nivolumab and ipilimumab in the NIVO3/IPI1 regimen as first-line therapy in patients with intermediate or poor-risk ccRCC. As potentially viable Phase 1b regimens are identified, additional patients may be enrolled into Phase 1 dose escalation regimens at or below the MTD to ensure sufficient safety experience and/or early evidence of clinical activity are available to recommend Phase 1b regimens. Phase 1 dose escalation and enrollment of additional patients at prior dose levels may proceed in parallel after safety evaluations are sufficiently mature as determined by the Investigator, in consultation with the Sponsor. In addition, decisions to remain at the current dose level may override decisions to dose escalate per TITE-BOIN design, based on clinical judgment of the Principal Investigator and in consultation with the Sponsor. Phase 1b dose expansion cohorts will evaluate sitravatinib at the recommended dose in combination with nivolumab and ipilimumab as first-line therapy in patients with intermediate/poor-risk ccRCC (Cohort A), and potentially in patients with favorable-risk ccRCC (Cohort B). In addition, future dose expansion cohorts may be added via protocol amendment to include other solid malignancies in which favorable activity has been previously demonstrated with nivolumab/ipilimumab combination treatment, such as metastatic CRC, melanoma, HCC, NSCLC, and UC.

If a tolerable dose is identified for sitravatinib in combination with NIVO3/IPI1 and evidence of activity is observed, the combination of sitravatinib and the alternate regimen NIVO1/IPI3 (1 mg/kg nivolumab and 3 mg/kg ipilimumab Q3W for 4 doses, then

nivolumab 240 mg every 2 weeks [Q2W] or 480 mg every 4 weeks [Q4W]) may be evaluated through dose escalation and dose expansion via a protocol amendment.

### 3.1 Phase 1 Dose Escalation

The study will begin with Phase 1 dose escalation of sitravatinib administered in combination with the NIVO3/IPI1 regimen as first-line therapy in patients with intermediate or poor-risk ccRCC. The starting dose for sitravatinib is 35 mg QD (Cohort 1), and planned dose escalation levels are outlined in Table 4. Dose de-escalation levels for ipilimumab may be evaluated as outlined in Table 5. Dose escalation/de-escalation will follow the time-to-event Bayesian optimal interval (TITE-BOIN) design described in Section 9.

Depending on the outcome of sitravatinib in combination with NIVO3/IPI1, Phase 1 dose escalation using the alternate regimen NIVO1/IPI3 may be implemented via a protocol amendment.

### 3.2 Phase 1b Dose Expansion

The Phase 1b portion of the study will be initiated following the identification of the recommended dose of sitravatinib in combination with nivolumab and ipilimumab. Depending on aggregate data from the escalation and expansion cohorts, lower doses of sitravatinib in combination with nivolumab and ipilimumab may also be explored during Phase 1b, if warranted.

Initially, the planned dose expansion cohorts will evaluate first-line treatment in patients with intermediate/ poor-risk ccRCC (Cohort A) and first-line treatment in patients with favorable-risk ccRCC (Cohort B). The decision to initiate Cohort B will be determined based on Phase 1 dose-escalation findings and as information becomes available supporting the NIVO3/IPI1 regimen in favorable-risk RCC.

Future dose expansion cohorts may include other solid malignancies in which favorable activity has been previously demonstrated with nivolumab/ipilimumab combination treatment, such as metastatic CRC, melanoma, HCC, NSCLC, and UC. These cohorts would be added via protocol amendment as information supporting expansion of the study becomes available.

## 4 SUBJECT SELECTION AND ENROLLMENT

Patient eligibility must be reviewed and documented by an appropriately qualified member of the Investigator’s study team before patients are enrolled into the study. No exceptions to the patient eligibility requirements will be granted by the Sponsor.

## 4.1 Inclusion Criteria

Patients must meet all of the following inclusion criteria to be eligible for enrollment into the study:

1. Histologically or cytologically confirmed diagnosis of advanced (not amenable to surgery or radiation therapy with curative intent) or metastatic disease, as follows:
    a. Dose escalation cohorts and Cohort A: Poor- or intermediate-risk, as per the International Metastatic RCC Database Consortium (IMDC) criteria (Appendix 1), RCC with a clear-cell component.
    b. Cohort B: Favorable-risk (as per IMDC criteria in Appendix 1) RCC with a clear-cell component.
2. Prior treatment history, as follows:
    a. Dose escalation cohorts and dose expansion Cohorts A and B: No prior treatment with systemic therapy, with the following exception: One prior adjuvant or neoadjuvant therapy for completely resectable RCC is allowed if such therapy did not include an agent that targets VEGF or VEGF receptors or any other antibody or drug targeting T-cell co-stimulation or checkpoint pathways (including, but not limited to: an anti-PD-1, anti-PD-L1, anti-PD-L2, anti-CD137, or anti-CTLA-4 antibody), and if recurrence occurred at least 6 months after the last dose of adjuvant or neoadjuvant therapy.
3. Measurable disease per RECIST 1.1.
4. Age ≥18 years.
5. Adequate bone marrow and organ function demonstrated by:
    a. Absolute neutrophil count ≥1,500/mm$^3$ (≥1.5 × 10$^9$/L).
    b. Hemoglobin ≥9.0 g/dL not dependent on transfusion support.
    c. Platelet count ≥100 × 10$^9$/L (≥100,000/mm$^3$).
    d. Alanine aminotransferase (ALT) and aspartate aminotransferase (AST) ≤2.5 × upper limit of normal [ULN], or ≤5.0 × ULN for patients with documented liver metastases.
    e. Serum bilirubin ≤1.5 × ULN or ≤3.0 × ULN for patients with Gilbert Syndrome or documented liver metastases.
    f. Serum creatinine ≤1.5 × ULN or creatinine clearance ≥40 mL/min, using the Cockcroft-Gault formula.

6. Eastern Cooperative Oncology Group (ECOG) performance status 0 or 1 (refer to Appendix 2).

7. Most recent prior therapy (eg, systemic or radiation therapy) discontinued at minimum of 2 weeks before the date of first on-study treatment.

8. Recovery from adverse effects of prior therapy to baseline or Grade 1 (excluding any grade alopecia, Grade ≤2 dysgeusia, and Grade ≤2 peripheral neuropathy).

9. Life expectancy of at least 3 months.

10. Women of child-bearing potential (WOCBP) or men whose partner is a WOCBP must agree to use contraception while participating in this study, and for a period of 6 months following the last dose of study treatment.

11. Completed informed consent process, including signing the Institutional Review Board (IRB)/Ethics Committee (EC)-approved informed consent form (ICF).

12. Willing to comply with clinical trial instructions and requirements.

## 4.2 Exclusion Criteria

Patients with any of the following will be excluded from participation in the study:

1. Known or suspected presence of another malignancy that could be mistaken for the malignancy under study during disease assessments or can negatively impact the prognosis of the disease under study.

2. Brain metastases.

3. Carcinomatous meningitis.

4. Active or prior documented autoimmune disease, as follows:

    a. Inflammatory bowel disease (eg, Crohn's disease, ulcerative colitis).

    b. Interstitial lung disease (ILD), drug-induced ILD, radiation pneumonitis which required steroid treatment, or any evidence of clinically active ILD.

    c. Other medically important autoimmune disease within 2 years before the first dose of study treatment.
    NOTE: Patients with Type 1 diabetes, vitiligo, Graves' disease requiring only hormone replacement, residual hypothyroidism requiring only hormone replacement, or psoriasis or Sjögren's syndrome not requiring systemic treatment (within the past 2 years) are permitted.

5. Immunocompromising conditions, as follows:

    a. Current or prior use of immunosuppressive medication within 28 days before the first dose of study treatment, with the exceptions of topical, ocular, intranasal and inhaled corticosteroids (with minimal systemic absorption) or systemic corticosteroids at physiological doses, which are not to exceed 10 mg/day of prednisone, or an equivalent corticosteroid. A brief course (≤3 days) of systemic corticosteroids >10 mg/day of prednisone (or equivalent corticosteroid) for prophylaxis (eg, for contrast dye allergy) or for treatment of non-immune conditions (eg, delayed-type hypersensitivity reaction caused by a contact allergen) is permitted within the 28 days.

    b. Known acute or chronic human immunodeficiency virus (HIV) infection.

    c. History of primary immunodeficiency.

    d. History of allogeneic transplant.

6. Uncontrolled adrenal insufficiency.

7. Use of live-attenuated vaccines that have the potential to replicate in humans against infectious disease (eg, varicella) within 28 days before the first dose of study treatment.

8. Known acute or chronic hepatitis B or hepatitis C infection. Patients treated for hepatitis C with no detectable viral load are permitted.

9. History of severe hypersensitivity reaction to any monoclonal antibody (mAb) or any study treatment excipients.

10. History of significant hemoptysis or hemorrhage within 4 weeks before the first dose of study treatment.

11. History of stroke or transient ischemic attack within 6 months before the first dose of study treatment.

12. History of deep vein thrombosis within the past 6 months unless adequately treated with appropriate anticoagulation (with the exception of warfarin treatment, which is prohibited on study).

13. History of pulmonary embolism within the past 6 months unless stable, asymptomatic, and treated with appropriate anticoagulation for at least 6 weeks (with the exception of warfarin treatment, which is prohibited on study).

14. Any of the following cardiac abnormalities:
    - Unstable angina pectoris within the past 6 months.
    - Symptomatic or uncontrolled atrial fibrillation within the past 6 months.
    - Congestive heart failure ≥NYHA Class 3 within the past 6 months.
    - Prolonged QTc interval on electrocardiogram (ECG) >480 msec.
    - Left ventricular ejection fraction (LVEF) <40%.
15. Uncontrolled arterial hypertension (>150 mm Hg systolic or >100 mm Hg diastolic) on multiple observations despite standard of care treatment.
16. History of abdominal fistula, gastrointestinal perforation, or intra-abdominal abscess within the past 6 months.
17. Need for treatment with proton pump inhibitors (PPIs). Patients may switch to use of antacids and/or H2 antagonist (H2A) medications.
18. Concomitant medication known to cause prolonged QTc that cannot be discontinued or changed to a different medication prior to enrollment (refer to Appendix 4, Examples of Drugs with a Known Risk of Torsades de Pointes).
19. Need for anticoagulation treatment with warfarin.
20. Major surgery within 4 weeks before the first dose of study treatment.
21. Serious, non-healing wound or ulcer.
22. Pregnancy. WOCBP must have a negative serum or urine pregnancy test documented within the screening period prior to start of study drug.
23. Breast-feeding or planning to breast-feed during the study or within 30 days after the last dose of sitravatinib, 3 months after the last dose of ipilimumab, or 5 months after the last dose of nivolumab, whichever occurs last.
24. Any serious illness, uncontrolled inter-current illness, psychiatric illness, active or uncontrolled infection, or other medical history, including laboratory results, which, in the Investigator's opinion, would be likely to interfere with the patient's participation in the study, or with the interpretation of the results.

### 4.3 Life Style Guidelines

Patients who are biologically capable of having children and sexually active must agree to use an acceptable method of contraception for the duration of the treatment period and for at least 6 months after the last dose of study treatment. The Investigator will counsel the patient on selection of contraception method and instruct the patient in its consistent and correct use. Examples of acceptable forms of contraception include:

1. Oral, inserted, injected or implanted hormonal methods of contraception, provided it has been used for an adequate period of time to ensure effectiveness.

2. Correctly placed copper containing intrauterine device (IUD).

3. Male condom or female condom used WITH a spermicide.

4. Male sterilization with confirmed absence of sperm in the post-vasectomy ejaculate.

5. Bilateral tubal ligation or bilateral salpingectomy.

The Investigator will instruct the patient to call immediately if the selected birth control method is discontinued or if pregnancy is known or suspected.

Men must not donate sperm while participating in the study.

Note: Women are considered post-menopausal and/or not of child bearing potential if they have had 12 months of natural (spontaneous) amenorrhea with an appropriate clinical profile (eg, age appropriate, history of vasomotor symptoms) or have had surgical bilateral oophorectomy (with or without hysterectomy) or tubal ligation at least 6 months ago. In case of any ambiguity, the reproductive status of the woman should be confirmed by hormone level assessment.

### 4.4 Randomization

Not applicable; patients will not be randomized in this study.

## 5 STUDY TREATMENTS

### 5.1 Study Treatment Overview

All patients will receive sitravatinib, nivolumab, and ipilimumab. Study treatment will be administered as 21-day cycles for Cycles 1-4 and 28-day cycles for Cycles 5+. Sitravatinib capsules are taken orally (PO) QD. Nivolumab and ipilimumab will be administered by IV infusion Q3W for Cycles 1-4, followed by nivolumab Q2W or Q4W for Cycles 5+ in accordance with the USPI and standard care.

On days when all three study drugs are to be administered, sitravatinib should be taken prior to the NIVO/IPI infusions. Other considerations for timing of sitravatinib dose versus study activities (eg, PK blood sampling, ECGs, vital sign assessments), are of particular importance and are outlined in Section 5.2.3 and Table 2.

Phase 1 dose escalation levels are shown in Table 4 for sitravatinib. Dosing will begin at 35 mg QD in Cohort 1 in combination with the fixed NIVO3/IPI1 regimen as shown in Table 5. Dose escalation decisions are outlined in Section 9. Regimens with lower ipilimumab starting doses (labeled as Combination Regimen Dose Levels -1 and -2 in Table 5) may be evaluated. In the event that dose de-escalation is needed per decision by TITE-BOIN, either the starting sitravatinib dose level or the starting ipilimumab dose level may be reduced for the next cohort of patients.

### Table 4: Sitravatinib Dose Escalation Levels

| **Sitravatinib Dose Level** | **Sitravatinib Daily Dose** |
|---|---|
| -1[1] | 20 mg PO QD |
| 1 | 35 mg PO QD |
| -2[1] | 50 mg PO QD |
| 2 | 70 mg PO QD |
| 3 | 100 mg PO QD |

Abbreviations: PO = orally; QD = once daily.

1 Sitravatinib Dose levels -1 and -2 will be enrolled only if de-escalation is needed after assessment of the next higher dose level (Dose Level 1 and Dose Level 2, respectively)

### Table 5: Nivolumab and Ipilimumab Dose Levels

| **Combination Regimen Dose Level** | **Nivolumab[1]** | **Ipilimumab[1]** |
|---|---|---|
| 1 | 3 mg/kg IV Q3W × 4 cycles, then 240 mg Q2W or 480 mg Q4W | 1 mg/kg IV Q3W × 4 cycles, then stop |
| -1[2] | | 0.7 mg/kg IV Q3W × 4 cycles, then stop |
| -2[2] | | 0.3 mg/kg IV Q3W × 4 cycles, then stop |

Abbreviations: IV = intravenous; Q2W = every 2 weeks; Q3W = every 3 weeks; Q4W every 4 weeks.

1 Nivolumab and ipilimumab should be administered according to USPI and standard care.

2 The 0.7 mg/kg and 0.3 mg/kg dose levels of ipilimumab will be enrolled only if de-escalation is needed after assessment of the next higher dose level (1 mg/kg and 0.7 mg/kg, respectively).

Guidelines for study drug administration and dose modification in the event of toxicity are provided below. If nivolumab or ipilimumab is interrupted or discontinued, administration of sitravatinib may be continued at the discretion of the Investigator and patient. In the event that nivolumab and ipilimumab, or maintenance nivolumab, are discontinued due to AEs, administration of sitravatinib may be escalated to dose levels up to and including 100 mg QD at the discretion of the Investigator and patient, after discussion with and agreement of the Sponsor's Medical Monitor. Dosing at 100 mg QD sitravatinib is the RP2D for sitravatinib monotherapy and for combinations with PD-(L)1 immune checkpoint inhibitors. In the event of treatment interruption/delay, the original cycle length (ie, 21 days for Cycles 1-4, 28 days for Cycles 5+) should be maintained unless all 3 study treatments are interrupted. In this case, the next cycle should start at the resumption of any of the 3 study treatments.

Patients may continue to receive study treatment at the discretion of the Investigator until disease progression, unacceptable AEs, patient refusal, or death. Patients experiencing clinical benefit in the judgment of the Investigator may continue study treatment beyond disease progression as defined by RECIST 1.1 if the progression is not rapid, symptomatic, or requiring urgent medical intervention. Patients considering continuation of study treatment beyond RECIST-defined disease progression must be provided with and sign an ICF outlining other approved therapies and any potential clinical benefit that the patient may be foregoing by continuing study treatment.

Patients discontinuing treatment will be followed for receipt of subsequent anticancer therapies and survival.

## 5.2 Sitravatinib

### *5.2.1 Sitravatinib Formulation, Packaging, and Storage*

The sitravatinib capsule product consists of a blend of sitravatinib malate drug substance, microcrystalline cellulose, mannitol, croscarmellose sodium, colloidal silicon dioxide, and magnesium stearate.

The dosage strength is expressed as the amount of sitravatinib equivalent, ie, the free base moiety of sitravatinib malate. Dose strengths of sitravatinib will be provided in the Study Pharmacy Manual.

Sitravatinib drug product is packaged in high-density polyethylene (HDPE), white opaque, round 60 cc bottles. A tamper-proof heat induction seal and a child-resistant closure are used. The provided bottles may be labeled for specific patient use and given to the patient.

Sitravatinib medication labels comply with the legal requirements of the US and all countries in which the clinical trial material will be used and will be printed in the languages required in the countries in which the study is conducted.

Investigational clinical trial material should be stored in an area that is secure, with limited access and monitored for temperature using a calibrated thermostat or thermometer. Sitravatinib capsules should be stored under the conditions stated on the container labels and the Study Pharmacy Manual.

Refer to the Study Pharmacy Manual for additional details.

### *5.2.2 Sitravatinib Preparation, Dispensing, and Accountability*

Only qualified personnel who are familiar with procedures that minimize undue exposure to them and to the environment should undertake the preparation, handling, and safe disposal of chemotherapeutic agents.

Study site personnel will dispense bottles containing sitravatinib capsules on Day 1 of each cycle. Sufficient supply will be provided for each cycle, and extra capsules may be provided to cover an additional 2 days in case of delayed clinic visits or lost capsules.

All sitravatinib study treatment supplies will be accounted for in the drug accountability inventory forms supplied by the Sponsor or using locally approved forms that include all required information. The drug accountability inventory forms must identify the study drug, including batch or lot numbers and account for its disposition on a patient-by-patient basis, including specific dates and quantities. The forms must be signed by the individual who dispensed the drug.

Patients will be asked to record their daily dosing on Sponsor provided diary cards and report any missed doses or lost doses at the next clinic visit. On the back of each Sponsor provided diary card, written dosing instructions for sitravatinib capsules are provided (eg, fasting instructions, take with water, etc). Patients should be told to bring study treatment bottle(s) (empty or not) and completed dosing diaries with them to the clinic visit for a compliance check and capsule count. Study site personnel will retain the bottle(s) until a monitor has completed reconciliation and retain dosing diaries with site study files.

At the end of the study, all unused sitravatinib drug supplies must be destroyed in accordance with local Standard Operating Procedure provided to the Sponsor for the Trial Master File, or returned to the Sponsor or its appointed agent, as directed by the Sponsor.

### *5.2.3 Sitravatinib Administration*

Sitravatinib capsules are taken PO QD in a continuous regimen expressed in cycles of 21 days (Cycles 1-4) or 28 days (Cycles 5+). The starting dose of sitravatinib will be 35 mg QD for Cohort 1.

The following guidelines should be followed for sitravatinib administration:

- Dosing in the morning is preferred.

- Capsules should be taken on an empty stomach (at least 2-hour fast before each dose and no food for a minimum of 1 hour after each dose) unless notified otherwise by the Sponsor. This requirement may be eliminated based on the outcome of ongoing evaluations. Any change in fasting will be implemented by Administrative Letter to Investigators and reiterated in the next protocol amendment required within the study.

- Capsules should be taken with at least 200 mL (approximately 1 cup) of water.

- Patients should swallow the capsules whole and not chew them; capsules should not be broken or crushed.

- If vomiting occurs after dosing, the dose should not be replaced.

- For patients who are on gastric acid medications, refer to Section 5.8.1 for further instructions.

- Missed doses may be taken if within 12 hours of the scheduled time, and the next dose should be taken at its scheduled time.

When sitravatinib and NIVO/IPI dosing are scheduled for the same day, the daily dose of sitravatinib should precede the NIVO/IPI infusions for logistical reasons. This order of dosing is of most interest on days when blood sampling is scheduled for sitravatinib PK, wherein the predose blood sampling, sitravatinib dosing, and 30 minute postdose blood sampling should all occur prior to the NIVO/IPI infusions (see Table 2). For all scheduled PK sampling days, patients should be instructed to withhold their daily dose of sitravatinib, as this dose will be taken at the clinic visit according to the timing indicated in Table 2.

### 5.2.4 Sitravatinib Dose Modification or Discontinuation

Sequential dose reductions are permitted for individual patients as outlined in Table 6. Guidelines for dose modifications to be implemented to manage AEs are described in Section 5.5. Dose reduction below 20 mg QD may be undertaken after discussion with the Sponsor.

Once the dose has been reduced, re-escalation may be considered on a case-by-case basis. If the administration of sitravatinib is interrupted for reasons other than toxicity, then treatment with the study drug may be resumed at the same dose.

**Table 6: Sitravatinib Sequential Dose Reductions for Individual Patients**

| |
|---|
| 100 mg PO QD |
| 70 mg PO QD |
| 50 mg PO QD |
| 35 mg PO QD |
| 20 mg PO QD |

Abbreviations: PO = orally; QD = once daily.

If treatment with sitravatinib is delayed for ≥14 days, then resumption at a reduced dose should be considered. If treatment with sitravatinib is withheld for ≥28 consecutive days for a sitravatinib toxicity, then permanent discontinuation from sitravatinib should be considered. If nivolumab or ipilimumab is interrupted or discontinued, administration of sitravatinib may be continued at the discretion of the Investigator and patient. In the event that nivolumab and ipilimumab, or maintenance nivolumab, are discontinued due to AEs, administration of sitravatinib may be escalated to dose levels up to and including 100 mg QD at the discretion of the Investigator and patient, after discussion with and agreement of the Sponsor's Medical Monitor.

## 5.3 Nivolumab

All administration information, including AE management, should be in accordance with the current OPDIVO® USPI and the Study Pharmacy Manual. Information and guidance outlined herein refers to the OPDIVO® USPI dated September 2019. Refer to the current OPDIVO® USPI provided by the manufacturer for updates during the conduct of this study.

### *5.3.1 Nivolumab Formulation, Packaging, and Storage*

Refer to the Study Pharmacy Manual and the current USPI.

### *5.3.2 Nivolumab Preparation and Dispensing*

Refer to the Study Pharmacy Manual and the current USPI.

### *5.3.3 Nivolumab Administration*

Refer to Table 5 for the nivolumab dose regimen. Nivolumab and ipilimumab will be administered on the same day. Refer to the Study Pharmacy Manual and the current USPI for additional administration details for the NIVO/IPI combination regimen.

Sitravatinib dosing should precede NIVO/IPI infusion. NIVO/IPI can be administered at any time after administration of sitravatinib, except on Cycle 1 Day 1, when the postdose

PK blood sample (at 30 minutes after sitravatinib administration) must be collected before administration of NIVO/IPI (refer to Table 2).

### *5.3.4 Nivolumab Dose Modification or Discontinuation*

Dose modifications (ie, interruption or discontinuation) for nivolumab should be performed per the current OPDIVO® USPI. If nivolumab is withheld, sitravatinib may be continued at the discretion of the Investigator and patient.

## 5.4 Ipilimumab

All administration information, including AE management, should be in accordance with the current YERVOY® USPI and the Study Pharmacy Manual. Information and guidance outlined herein refers to the YERVOY® USPI dated May 2019. Refer to the current YERVOY® USPI provided by the manufacturer for updates during the conduct of this study.

### *5.4.1 Ipilimumab Formulation, Packaging, and Storage*

Refer to the Study Pharmacy Manual and the current USPI.

### *5.4.2 Ipilimumab Preparation and Dispensing*

Refer to the Study Pharmacy Manual and the current USPI.

### *5.4.3 Ipilimumab Administration*

Refer to Table 5 for the ipilimumab dose regimen. Nivolumab and ipilimumab will be administered on the same day. Refer to the Study Pharmacy Manual and the current YERVOY® USPI for additional administration details for the NIVO/IPI combination regimen.

Sitravatinib dosing should precede NIVO/IPI infusion. NIVO/IPI can be administered at any time after administration of sitravatinib, except on Cycle 1 Day 1, when the postdose PK blood sample (at 30 minutes after sitravatinib administration) must be collected before administration of NIVO/IPI (refer to Table 2).

### *5.4.4 Ipilimumab Dose Modification or Discontinuation*

Dose modifications (ie, interruption or discontinuation) for ipilimumab should be performed per the current YERVOY® USPI. If ipilimumab is withheld, sitravatinib may be continued at the discretion of the Investigator and patient.

## 5.5 Management of Adverse Events

### *5.5.1 Sitravatinib-Related Adverse Events*

In the event of symptomatic sitravatinib-related AEs, dose reduction to a level that can be administered continuously is preferred over continued dosing until interruption becomes necessary. Additional guidelines are provided herein for management of non-hematological AEs, hematological AEs, and other potential AEs more specific to treatment with sitravatinib or agents in the same class of cancer treatment.

#### *5.5.1.1 General Management of Non-Hematological Toxicities*

Recommended sitravatinib dose modification guidelines for sitravatinib-related non-hematological AEs are provided in Table 7. Refer to Table 6 for dose reduction levels.

**Table 7: Sitravatinib Dose Modifications: Non-Hematological Toxicities**

| Toxicity[1] | Treatment Interruption | Dose Reduction |
|---|---|---|
| Grade 1 | Continue treatment unchanged. | |
| Grade 2 Asymptomatic | May be implemented based on Investigator discretion. | |
| Grade 2 Symptomatic | May be implemented based on Investigator discretion. | Dose reduction to the next lower dose level is recommended over treatment interruption as early as Cycle 2. |
| Grade 3 or 4 | Hold treatment until Grade ≤1 or return to baseline. Exception presented in footnotes.[2] | Resume at ≥1 dose level below the dose inducing the toxicity. Exceptions presented in footnotes.[2,3] |

1 Management of selected AEs for sitravatinib are presented in Section 5.5.1.3.
2 Neither dose interruption nor dose reduction is required for asymptomatic increases in amylase and/or lipase in the absence of other clinical evidence of pancreatitis (eg, electrolyte abnormalities, radiographic changes).
3 Dose reduction is not required for Grade 3/4 electrolyte abnormality that is not clinically complicated and resolves spontaneously or with conventional medical treatment within 72 hours. Treatment interruption is required.

Symptomatic Grade 2 sitravatinib-related non-hematological AEs occurring as early as Cycle 2 are recommended to be managed using dose reduction to the next lower dose level rather than continued dosing until interruption becomes necessary.

Sitravatinib-related non-hematological toxicities Grade ≥3 should be managed with sitravatinib interruption and dose reduction, until resolution of toxicity to Grade ≤1 or to baseline value as described in Table 7. Recurrence of the toxicity may be managed similarly. If treatment is interrupted for ≥28 days due to sitravatinib toxicity, permanent discontinuation from sitravatinib should be considered. Note the following exceptions:

- Neither dose interruption nor dose reduction is required for asymptomatic increases in amylase and/or lipase in the absence of other clinical evidence of pancreatitis (eg, electrolyte abnormalities, radiographic changes).
- Dose reduction is not required for Grade 3/4 electrolyte abnormality that is not clinically complicated and resolves spontaneously or with conventional medical treatment within 72 hours. Treatment interruption is required.

#### *5.5.1.2 General Management of Hematological Toxicities*

Hematological toxicities are not a frequent cause of treatment interruption or discontinuation of sitravatinib treatment. Observed Grade ≥3 hematological events that are considered to be causally related to sitravatinib should initially be managed using treatment interruption. Treatment may resume once the event returns to Grade 2 or baseline.

In addition, dose reduction of sitravatinib should be implemented in the following cases:

- Grade 3 or 4 febrile neutropenia;
- Grade 4 neutropenia persisting for ≥8 days; or
- Grade 4 thrombocytopenia of any duration or Grade 3 thrombocytopenia with bleeding.

#### *5.5.1.3 Management of Selected Adverse Events*

The following are guidelines for management of potential AEs more specific to treatment with sitravatinib or agents in the same class of cancer treatment.

##### 5.5.1.3.1 Hypertension

Hypertension, including Grade 4 events, has been reported with sitravatinib. Dihydropyridine calcium channel blockers such as nifedipine, amlodipine, and nicardipine may be considered if anti-hypertensive therapy is required and should be considered for patients with Grade 3 hypertension without clinically significant increases in blood pressure (see Table 8).

### Table 8: Sitravatinib Dose Modification for Increased Blood Pressure

| Toxicity | Treatment Interruption | Dose Reduction |
|---|---|---|
| Grade 1 or 2 hypertension | Investigator discretion. | |
| Grade 3 hypertension without clinically significant increases in BP as defined below | Investigator discretion. Consider anti-hypertensives. | |
| Grade 3 hypertension with clinically significant increases in BP, defined as EITHER an increase of ≥30 mmHg in systolic BP to ≥180 mmHg, OR increase of ≥20 mmHg in diastolic BP to ≥110 mmHg, confirmed with repeated testing after at least 5 minutes | Hold until Grade ≤2 or return to baseline. | Investigator discretion. |
| Grade 4 hypertension | Discontinue sitravatinib. | |

Abbreviations: BP = blood pressure.

In cases of Grade 3 hypertension with clinically significant increases in blood pressure, suspension of sitravatinib dosing is recommended until blood pressure is controlled (Grade ≤2 or return to baseline). Treatment with sitravatinib may resume at the same or a lower dose at the discretion of the Investigator. If significant hypertension recurs, options include change in medical management of the patient, reduction of sitravatinib dose, or discontinuation of study treatment, at the discretion of the Investigator. In the event of Grade 4 hypertension, sitravatinib should be permanently discontinued.

#### 5.5.1.3.2 Palmar-Plantar Erythrodysesthesia

PPE has been reported with sitravatinib. Signs and symptoms of PPE include redness, swelling, pain, and less commonly blisters on the palms of the hands and/or the soles of the feet. Patients who develop PPE should be counseled on measures to mitigate the effects of PPE. Such measures include avoidance of exposure of hands and feet to hot water when washing dishes or bathing, or to other sources of heat, avoidance of activities that cause unnecessary force or friction (rubbing) on the hands or feet, avoiding contact with harsh chemicals such as cleaning products, use of tools or household items that result in pressure on the hands, such as garden tools, knives, and screwdrivers, and wearing of loose fitting, well-ventilated shoes and clothes. Treatment may include use of topical moisturizing agents, topical anesthetics, or topical anti-inflammatory medications such as corticosteroid creams. In more severe cases, dose interruption and reduction may be warranted.

#### 5.5.1.3.3 Diarrhea

See Section 5.5.4.1 for additional guidance for patients experiencing diarrhea during study treatment. Diarrhea has been reported with sitravatinib treatment as with other small molecule RTK inhibitors, though the mechanism remains unclear. Patients should be counseled that diarrhea is a possible side effect and advised to take loperamide or a similar medication as needed if diarrhea develops. Diarrhea due to sitravatinib typically improves within several days if sitravatinib treatment is interrupted or dose reduced. Any

patients developing dehydration or clinically significant electrolyte abnormalities should interrupt treatment, but treatment may be restarted once diarrhea is controlled. Investigators should also evaluate whether diarrhea may be attributable to the irAE of colitis as described in Section 5.5.4.1.

#### 5.5.1.3.4 Hemorrhagic Events

The risk of hemorrhagic events with sitravatinib is unknown; however, such events have been reported with inhibitors of VEGFR. Patients with active hemoptysis or gastrointestinal bleeding should not take sitravatinib, and suspension of treatment is recommended for patients developing clinically significant bleeding.

#### 5.5.1.3.5 Thrombotic Events

Arterial and venous thrombotic events have been observed with sitravatinib and described with other inhibitors of the VEGFR pathway. The majority of thrombotic events observed with sitravatinib have been venous thrombotic events. The occurrence of thrombotic events with sitravatinib is being monitored for further characterization. Precautions should be taken in patients with recent, clinically significant thrombotic events, and treatment should be discontinued in patients who develop clinically significant thromboembolic complications.

#### 5.5.1.3.6 Thyroid Dysfunction

Hypothyroidism and blood thyroid-stimulating hormone (TSH) increased have been reported in patients taking sitravatinib, consistent with literature reports of thyroid dysfunction with RTK inhibitors, which are typically reported as hypothyroidism that is sometimes preceded by a brief period of hyperthyroidism. TSH should be monitored during treatment with sitravatinib. Patients diagnosed with hypothyroidism should be treated with thyroid replacement and may continue treatment with sitravatinib. When sitravatinib is used in combination with checkpoint inhibitors, thyroid dysfunction, including thyroiditis and hypothyroidism, may be immune-mediated.

#### 5.5.1.3.7 Decreased Left Ventricular Ejection Fraction (LVEF)

Decreased LVEF has been observed with sitravatinib. In cases where LVEF decreases by ≥20% to an LVEF <50%, the dose of sitravatinib should be interrupted and/or reduced. Permanent discontinuation should be considered for patients requiring acute hospitalization for treatment of congestive heart failure.

#### 5.5.1.3.8 Proteinuria

Proteinuria has been observed with sitravatinib and described with other inhibitors of the VEGFR pathway. Urinalysis for urine protein should be performed prior to treatment and as clinically warranted during treatment. Patients who develop ≥2+ proteinuria should undergo 24-hour urine collection for assessment of urine protein; treatment with

sitravatinib should be discontinued in the presence of ≥2 grams of proteinuria/24 hours and may be restarted when protein levels decrease to <2 grams/24 hours. Patients who develop nephrotic syndrome should be discontinued from treatment with sitravatinib.

##### 5.5.1.3.9 Increased Amylase and Lipase

Increased amylase and lipase have been observed in patients treated with sitravatinib. Although the mechanism has not been fully elucidated, inhibition of VEGF may lead to acinar cell apoptosis resulting in the release of autodigestive enzymes (Sevin-2012). Accordingly, increases in amylase and lipase, and pancreatitis have been reported with other inhibitors of the VEGF pathway. Most sitravatinib treatment-emergent events of increased amylase and lipase were asymptomatic while some were associated with signs and/or symptoms of pancreatitis. Treatment with sitravatinib may continue without dose modification (eg, interruption or reduction) in cases of asymptomatic amylase and/or lipase increases in the absence of other clinical evidence of pancreatitis (eg, symptoms, electrolyte abnormalities, radiographic changes) at the investigator's discretion. Sitravatinib should be interrupted for any grade pancreatitis and the patient managed according to standard of care. After resolution of pancreatitis, sitravatinib resumption is at the discretion of the investigator; if pancreatitis is assessed as sitravatinib treatment-related and treatment is resumed, a dose reduction is recommended.

##### 5.5.1.3.10 Other Sitravatinib-Related Events

Other sitravatinib-related events include decreased appetite, dysphonia, fatigue, nausea, stomatitis, and vomiting. Management should be consistent with standard of care and sitravatinib dose modification as described in the protocol for each study.

### *5.5.2 Nivolumab Adverse Event Management Guidelines*

Refer to the current USPI for OPDIVO® for guidance concerning management of AEs, including irAEs, during treatment with nivolumab. If nivolumab is withheld sitravatinib may be continued at the discretion of the Investigator and patient.

### *5.5.3 Ipilimumab Adverse Event Management Guidelines*

Refer to the current USPI for YERVOY® for guidance concerning management of AEs, including irAEs, during treatment with ipilimumab. If ipilimumab is withheld, sitravatinib may be continued at the discretion of the Investigator and patient.

### *5.5.4 Management of Immune-Related Adverse Events*

An irAE is defined as an AE that is associated with drug exposure and is consistent with an immune-mediated mechanism of action and where there is no clear alternative etiology. Serologic, immunologic, and histologic (biopsy) data, as appropriate, should be used to support an irAE diagnosis. Appropriate efforts should be made to rule out neoplastic, infectious, metabolic, toxin, or other etiologic causes of the irAE

Sitravatinib has not been associated with irAEs. However, the potential exists for sitravatinib to contribute to irAEs associated with nivolumab and ipilimumab treatment. In the event of a Grade ≥2 irAE during study treatment, administration of sitravatinib should be interrupted until the event stabilizes to Grade ≤1. At the time of resumption of sitravatinib administration, a dose reduction may be implemented at the discretion of the Investigator.

Dose modification of nivolumab and ipilimumab for irAEs should be managed as detailed in the current USPIs for each agent.

#### *5.5.4.1 Diarrhea/Colitis*

The management of diarrhea should be guided by clinical judgment and an assessment of the most likely causative etiology, with special consideration given to the potential for immune-mediated colitis. The presence of abdominal pain, mucus or blood in the stool or peritoneal signs should raise the index of suspicion for immune-mediated colitis, as these features are generally not observed with sitravatinib treatment-associated diarrhea. The diarrhea observed with sitravatinib generally improves within several days of interrupting study medication. However, if any features of the clinical presentation, including timing of presentation, failure to improve with dose interruption, laboratory or radiologic tests suggests the presence of immune-mediated colitis, all study medications should be withheld and treatment with immuno-suppressive therapy initiated as detailed in the current USPIs for nivolumab and ipilimumab.

#### *5.5.4.2 Increased Transaminases*

The management of increases in AST and ALT should be guided by the clinical judgment of the Investigator, including an assessment of the most likely causative etiology, with special consideration given to the potential for immune-mediated hepatitis. Increased transaminases should be evaluated to determine whether confounding factors exist, such as viral infection, metastatic lesions or biliary obstruction.

For cases where transaminase increases are not likely to be immune-mediated, treatment management decisions should be made using Investigator discretion in consideration of clinical factors. Recommended treatment modifications for sitravatinib are provided in Table 9. However, if any features of the clinical presentation, including timing of presentation, failure to improve with dose interruption, laboratory or radiologic tests suggests the presence of immune-mediated hepatitis, all study medications should be withheld and treatment with immuno-suppressive therapy initiated as detailed in the current USPIs for nivolumab and ipilimumab.

Study treatment should be permanently discontinued for Hy's Law cases. Refer to Section 8.2.1.1 for Hy's Law criteria and reporting requirements.

**Table 9: Sitravatinib Dose Modification for Increased Hepatic Transaminase**

| Toxicity | Treatment Delay | Dose Modification |
|---|---|---|
| Grade 1 (>ULN to 3.0 × ULN) | May be implemented based on Investigator and patient discretion. | |
| Grade 2 (>3.0 to 5.0 × ULN) | Not required. | Decrease by 1 dose level. |
| Grade 3/4 (>5.0 × ULN) | Hold until Grade ≤1 or return to baseline. | If resolution occurs within 29 days, decrease by 1 dose level.<br>If no resolution within 29 days, discontinue sitravatinib. |

Note: The current USPIs for OPDIVO® and YERVOY® must be consulted to determine appropriate dose modifications for nivolumab and ipilimumab, respectively.
Abbreviations: ULN = upper limit of normal.

## 5.6 Assessment of Dose-Limiting Toxicity (Phase 1)

The duration of the DLT observation period is the first 9 weeks of study treatment for patients enrolled in Phase 1. The selection of this relatively long DLT observation period is based on irAEs associated with immunotherapies such as nivolumab and ipilimumab that are typically observed after several weeks of treatment. In contrast, DLTs associated with single-agent sitravatinib (eg, diarrhea, fatigue, PPE) have a more rapid onset, typically presenting within 3 weeks of treatment initiation.

Patients are considered DLT-evaluable if they experience a DLT during the first 9 weeks of treatment or if they received at least 80% of the planned dose of sitravatinib in the first 3 weeks, and at least 3 doses of both nivolumab and ipilimumab in the first 9 weeks. Patients who are not evaluable for DLT may be replaced. Dose escalations and de-escalations will be made based on available evaluable patients according to the BOIN decision rule (Appendix 3, Table 15).

### *5.6.1 DLT Definition for Non-immune Related AEs*

The following non-immune related AEs are considered DLTs if they occur within the first 3 weeks of treatment and are considered related to sitravatinib, regardless of relationship to nivolumab and/or ipilimumab. The purpose of this shorter window is to capture AEs that fall within the known toxicity profile for sitravatinib. NOTE: The occurrence of these AEs after the first 3 weeks of study treatment will not necessarily qualify as a DLT.

- Grade 5 AE;
- Grade ≥4 hematologic AE lasting ≥4 days;
- Grade ≥3 thrombocytopenia associated with clinically significant bleeding;
- Grade ≥3 febrile neutropenia;

- Grade ≥4 non-hematologic AE;
- Grade 3 hypertension that cannot be controlled with medical therapy, including:
    - Severe hypertension with systolic blood pressure ≥180 mmHg or diastolic blood pressure ≥120 mmHg, on more than one occasion;
    - Sustained uncontrolled hypertension, with systolic blood pressure ≥160 mmHg (but <180 mmHg) or diastolic blood pressure ≥100 mmHg (but <120 mmHg) lasting for ≥14 days or causing treatment delay for ≥4 days.
- Other Grade 3 non-hematologic AEs not related to underlying malignancy and lasting for >3 days despite optimal supportive care, with the following exceptions:
    - Fatigue that persists for ≤7 days;
    - Rash that resolves to Grade ≤1 within 3 weeks;
    - Elevation in serum amylase and/or lipase not associated with clinical or radiological evidence of pancreatitis;
    - Tumor flare (defined as local pain, irritation, or rash localized at sites of known or suspected tumor).
- Any other toxic effect during the first 3 weeks may be assessed as a DLT if, upon review by the Investigators and Sponsor, it is agreed that the toxicity was of sufficient severity to be considered dose limiting.

### 5.6.2 DLT Definition for Immune-Related AEs

The following irAEs are considered DLTs if they occur within the first 9 weeks of treatment and are considered related to nivolumab and/or ipilimumab, regardless of relationship to sitravatinib. The purpose of this longer window is to capture irAEs that fall within the known, late-onset toxicities for nivolumab and/or ipilimumab.

- Grade 5 irAE;
- Grade 4 hematologic irAE, with the exception of:
    - Lymphopenia;
    - Neutropenia lasting ≤2 days that is not associated with fever or other clinically significant symptoms.
- Grade 4 non-hematologic irAE;

- Grade 3 non-hematologic irAE that does not resolve to Grade ≤1 or baseline with immunosuppressive therapy within 3 weeks, with the following exceptions:
    - Any endocrinopathy (thyroid, pituitary, and/or adrenal insufficiency) that is adequately controlled by hormonal replacement;
    - Tumor flare (defined as local pain, irritation, or rash localized at sites of known or suspected tumor);
    - Infusion-related reaction that resolves to Grade ≤2 within 6 hours;
    - Electrolyte imbalance/abnormality that is not associated with clinical sequelae and resolves within 3 days spontaneously or with supplementation/appropriate management.
- Grade 3 central nervous system (CNS)-related irAE regardless of duration or reversibility.
- Any Grade ≥2 irAE of eye pain or reduction of visual acuity that does not respond to topical therapy and does not improve to Grade 1 severity within 2 weeks of the initiation of topical therapy or requires systemic treatment.
- Any other toxic effect during the first 9 weeks may be assessed as a DLT if, upon review by the Investigators and Sponsor, it is agreed that the toxicity was of sufficient severity to be considered dose limiting.

## 5.7 Medication Error

Medication errors may involve patient exposure to a wrong study drug, at a wrong dosing frequency, or at a wrong dose level (eg, a dose that is not planned in the study). Medication errors occurring during the conduct of this study will be documented as AEs (regardless of whether clinical signs or symptoms are observed) and if serious consequences are observed, will be reported on SAE forms. In all cases of medication error, the Sponsor should be notified immediately.

There is currently no specific treatment in the event of an overdose of sitravatinib, nivolumab, or ipilimumab. The Investigator will use clinical judgment to treat any overdose.

## 5.8 Concomitant Therapies

### *5.8.1 Concomitant Medications*

Prior medications, including medications administered long term, used within the 28-day period preceding Day 1 of the study, will be reviewed and recorded. Anti-cancer treatment will not be recorded as a prior medication but will be listed separately.

Concomitant medications must be locally approved and used at doses and regimens that are considered standard-of-care for the treated indication. Treatment for comorbidities, disease signs and symptoms and AEs should be provided as necessary in the judgment of the Investigator. Patients may continue to use any ongoing medications not prohibited by the inclusion/exclusion criteria or treatment plan. Refer to Appendix 4 for medications to be avoided or used with caution during sitravatinib treatment. Refer to the current USPIs for medications to be avoided or used with caution during treatment with nivolumab and ipilimumab.

**Anti-diarrheals:** In general, patients should be counseled that diarrhea is a possible side effect of the study treatments and advised to take loperamide or a similar medication as needed if diarrhea develops.

**Anti-emetics:** Patients may be premedicated for nausea and vomiting. Recommended anti-emetic agents include granisetron 1 mg as premedication, and then granisetron and/or prochlorperazine as needed.

**Gastric Acid Medications with Sitravatinib:** PPIs should be avoided during on-study treatment with sitravatinib. Switching from PPIs to H2 antagonists (H2As) or antacids is preferred. The use of H2As/antacids should adhere to the following guidance:

- H2A: sitravatinib should be taken at least 2 hours before and 10-12 hours after H2A administration
- Antacids: sitravatinib should be taken at least 2 hours before or 2 hours after antacid administration

**Medications with QTc Prolonging Activity with Sitravatinib:** The risk of QTc prolongation in patients receiving sitravatinib has not been characterized. Use of medications known to prolong QTc and pose risk of Torsades de Pointes (examples listed in Appendix 4, Drugs with a Known Risk of Torsades de Pointes) is to be avoided during sitravatinib treatment. Use of medications with conditional risk of Torsades de Pointes should be used with caution during sitravatinib treatment (examples listed in Appendix 4, Drugs with Conditional Risk of Torsades de Pointes).

**P-gp and BCRP substrates with Sitravatinib:** Sitravatinib is a strong inhibitor of P-gp and BCRP transporters (Section 1.1.2.2.2). Concomitant medications that are sensitive substrates or substrates with narrow therapeutic index for these transporters (examples listed in Appendix 4) should be used with caution during sitravatinib treatment.

**CYP3A4 Substrates with Sitravatinib:** In vitro data imply that sitravatinib is a strong direct inhibitor of CYP3A4 (Section 1.1.2.2.2). Concomitant medications that are sensitive substrates or substrates with narrow therapeutic index for CYP3A4 (examples listed in Appendix 4) should be used with caution during sitravatinib treatment.

**Herbal Medications/Preparations:** Herbal medications and preparations should be avoided throughout the study. Herbal medications include, but are not limited to: St. John's wort, Kava, ephedra (ma huang), gingko biloba, dehydroepiandrosterone (DHEA), yohimbe (yohimbine), saw palmetto, and ginseng.

**Transfusions:** Patients may receive transfusions as necessary.

**Antibiotics:** Antibiotics should be used as needed. Patients with neutropenic fever or infection should be treated promptly.

**Supportive Care/Palliative Care:** Supportive and palliative care* for disease-related symptoms may be administered at the Investigator's discretion, including the use of analgesics.

> *Bisphosphonates (eg, zolendronic acid) and RANK-L inhibitors (eg, denosumab) should be initiated prior to the first dose of study treatment.

**Growth Factors:** Therapeutic colony-stimulating factors should be used in accordance with American Society of Clinical Oncology (ASCO) guidelines.

**Immunosuppressive Medications:** Use of immunosuppressive mediations should be limited to the extent possible to allow testing of the immune-stimulatory mechanisms proposed in this clinical trial. Immunosuppressive medications should be used as needed to manage irAEs and the extent required to manage comorbidities and symptoms of disease.

**Vaccines:** Live attenuated vaccines that have the potential to replicate in humans are to be avoided within 100 days following the last dose of nivolumab or ipilimumab, whichever is later.

### 5.8.2 Concomitant Surgery or Radiation Therapy

The use of surgery to manage cancer lesions during study treatment is discouraged, with the exception of cytoreductive nephrectomy performed per the guidance outlined in Section 5.8.2.1. The impact of sitravatinib on wound healing has not yet been characterized. For patients with bone involvement, any foreseeable need for palliative radiotherapy should be addressed before study entry, if possible and clinically appropriate (eg, bone lesions at risk for spontaneous micro-fractures or painful lesions). However, these treatments may be used in cases where it is medically necessary. If radiotherapy is required, the Sponsor will provide guidance on duration of sitravatinib interruption.

In the event that major surgery is needed during study treatment, the patient should, if possible, interrupt dosing with sitravatinib 2 weeks in advance of the surgery and resume dosing 2 weeks after the surgery and adequate wound healing.

#### 5.8.2.1 *Cytoreductive Nephrectomy*

Cytoreductive nephrectomy is allowed after 6 months of study treatment in patients with RCC who continue to have a confirmed partial response [PR]. The decision to perform cytoreductive nephrectomy should be based on the following guidelines on a case-by-case basis in consultation with the Sponsor.

Relative indications:

- Ongoing symptoms such as hematuria; and
- Persistent presence of inferior vena cava thrombus despite study treatment.

Relative contraindications:

- Liver, bone, or CNS metastases;
- ECOG ≥2;
- Inadequate organ function, such as liver failure; or
- Less than 75% of the tumor bulk will be removed by the nephrectomy.

### *5.8.3 Other Anticancer or Experimental Therapy*

Use of approved or investigational anticancer treatment will not be permitted during the study treatment period, including chemotherapy, biological response modifiers, hormone therapy*, or immunotherapy. No other investigational drug may be used during treatment on this protocol. Concurrent participation in another therapeutic clinical trial is not allowed.

*Certain ongoing hormonal therapies taken to prevent recurrence of a malignancy not under study (eg, tamoxifen/aromatase inhibitor for breast cancer) may be permitted after discussion and agreement with the Sponsor.

# 6 STUDY ASSESSMENTS

## 6.1 Screening

Voluntary, written, dated, and signed informed consent must be obtained for each patient before any study-specific procedures are performed.

## 6.2 Study Period

For details on procedures during the study period, refer to Table 1, Table 2, and Table 3, and to procedure descriptions in Section 7.

## 6.3 Follow-up Visits

Refer to Table 3 and Section 7.4 for evaluations to be performed at follow-up visits beginning after the decision to stop all study treatment. All patients will be followed for non-irAEs for at least 28 days after the last dose of study treatment and followed for irAEs for at least 100 days after the last dose of study treatment, or until the start of subsequent anticancer therapy, whichever occurs first.

## 6.4 Long-term Follow-up

Survival status and subsequent anticancer therapies will be collected during long-term follow-up as outlined in Table 3 and Section 7.4 until death or lost to follow up. Follow up may be performed by telephone or email. Treatments received following participation in the study will be collected in the case report form (CRF).

## 6.5 Patient Discontinuation/Withdrawal

Patients may discontinue from study treatment or from study follow-up at any time at their own request, or they may be discontinued at any time at the discretion of the Investigator or Sponsor for safety, behavioral reasons, or the inability of the patient to comply with the protocol-required study visits/procedures.

Criteria that may be used to discontinue patients from receipt of study treatment includes, but is not be limited to, the following:

- Objective progressive disease (PD) according to RECIST 1.1 as determined by the Investigator (patients who may derive clinical benefit may continue on treatment at the discretion of the Investigator);
- Global deterioration of health status requiring discontinuation;
- Adverse event;
- Significant protocol violation;
- Lost to follow-up;
- Refusal for further treatment;
- Study termination by Sponsor;
- Pregnancy;
- Death.

Reasons for discontinuation from study follow-up may include:

- Study terminated by Sponsor;
- Lost to follow-up;
- Refusal for further follow-up for survival;
- Death.

If a patient does not return for a scheduled visit, every effort should be made to contact the patient. At least 2 attempts should be made to contact the patient, and each attempt should be recorded in the source documents. In any circumstance, every effort should be made to document patient outcome, if possible. The Investigator should inquire about the reason for withdrawal, request that the patient returns for a final visit, and if applicable, follow-up with the patient regarding any unresolved AEs.

If the patient withdraws from the study treatment and also withdraws consent for disclosure of future information, no further evaluations should be performed, and no additional data should be collected. The Sponsor may retain and continue to use any data collected before such refusal for further follow-up.

# 7 PROCEDURES

Every effort should be made to ensure that the protocol-required tests and procedures are completed as described. However, it is anticipated that there may be circumstances outside of the control of the Investigator that may make it infeasible to perform a protocol-specified assessment. In these cases, the Investigator will take all steps necessary to ensure the safety and well-being of the patient. When a protocol required test cannot be performed, the Investigator will document in the source document and CRF the reason and any corrective and preventive actions which he/she has taken to ensure that normal processes are adhered to as soon as possible. The study team will be informed of these incidents in a timely fashion.

## 7.1 Efficacy

### *7.1.1 Radiographic Disease Assessment*

All patients enrolled in the study are to be evaluated for disease activity as outlined in the Table 1. All known and suspected sites of disease should be evaluated at each assessment. At screening/baseline tumor assessments are to include computed tomography (CT) scan of the chest; CT or magnetic resonance imaging (MRI) of abdomen and pelvis; whole body bone scan (or positron emission tomography [PET] or PET/CT if local standard for clinical trials); CT with contrast or MRI of the brain; and evaluation of any superficial lesions. The subsequent on-study disease assessments will include imaging of all known and suspected sites of disease. In particular, in patients with bone disease at baseline, bone scans should be performed at suspicion of progression of bone lesions and during assessment for confirmation of CR.

CT scans should be performed with contrast agents unless contraindicated for medical reasons. If IV contrast is medically contraindicated, the imaging modality to be used (either CT without contrast or MRI) should be the modality that best evaluates the disease. The same imaging modality should be used for an individual patient throughout their participation in the study. Depending on the adequacy for evaluation of disease, a combination of CT without contrast and MRI should most often be used. CT without contrast is preferred for evaluation of lesions in lung parenchyma. MRI is not adequate for evaluation of lung parenchyma but should also be performed to evaluate all other aspects of the chest. MRI of the abdomen should substitute for CT with contrast unless the method does not adequately depict the individual's disease, in which case CT without contrast is preferred.

Disease response will be assessed in accordance with RECIST 1.1 (Eisenhauer-2009). Appendix 5 provides guidance in using the response criteria and includes modifications to RECIST 1.1 to address potential temporary treatment effects including tumor necrosis, cavitation, flare response, or pseudoprogression. The Investigator's assessment of disease response and progression will be the basis for patient management. Assessments will be performed until objective disease progression is documented by the Investigator, or until the beginning of subsequent anticancer therapy.

Patients experiencing tumor response (PR or CR) should undergo confirmatory assessment no sooner than 4 weeks after initial documentation; it is acceptable to perform confirmatory assessments at the next appointed evaluation per protocol (ie, 8 weeks after the observation of tumor response). In patients for whom bone lesions were identified at the baseline, bone scan is required as an element of the confirmation CR.

Potential exists for individual patients to experience tumor flare or pseudoprogression, or for individual tumor lesions to cavitate or become otherwise difficult to evaluate for a period of time as the result of beneficial study treatment impact. For this reason, in patients who are otherwise clinically stable, Investigators may delay reaching the conclusion of disease progression until subsequent on-study disease assessments are performed. Confirmatory assessments may be performed at least 4 weeks after the initial documentation; it is acceptable to perform confirmatory assessments at the next appointed evaluation per protocol (ie, 8 weeks after the observation of tumor increase).

Patients experiencing early radiographic progression accompanied by deteriorating health status should be evaluated for the possibility of hyperprogression (Kurman-2018). Patients experiencing rapid clinical and radiographic progression of disease should be discontinued from study treatment.

## 7.2 Safety Assessments

### *7.2.1 Medical History*

Medical history, including clinically significant past and present medical conditions, will be recorded. Cancer history will be recorded separately. Signs and symptoms of the

patient's cancer diagnosis and/or comorbidities present on Day 1 of study treatment and throughout treatment will be recorded in the CRF as AEs. The actual date of onset should be recorded in all cases. Thus, for signs and symptoms present on Day 1, the date of onset may pre-date start of study treatment.

### 7.2.2 Physical Examination, Vital Signs, and Performance Status

A physical examination including all major body systems is mandated at Screening and at the 7-Day Follow-up visits only. Height will be recorded at Screening only. Symptom-directed physical examinations will be performed at other visits as outlined in Table 1 and Table 3. Vital signs to be assessed include weight, body temperature, blood pressure, and pulse rate. On days when both vital signs and PK sampling are scheduled, the vital signs should be assessed prior to blood sampling as outlined in Table 2.

Clinically significant findings noted during screening will be reflected on the medical history CRF, while those noted on Day 1 of study treatment and throughout study treatment will be collected on the AE CRFs.

ECOG performance status will be assessed at the time points outlined in Table 1 and Table 3 according to guidelines in Appendix 2. Note that ECOG assessment at Screening should correlate with the Karnofsky Performance Status (KPS) assessment component of IDMC staging for patients with RCC (refer to Table 14).

### 7.2.3 Laboratory Safety Assessments

Laboratory tests will be drawn at the time points described in Table 1 and Table 3 and analyzed at local laboratories. Additional laboratory tests may be performed per standard of care, at the Investigator's discretion, for the purpose of planning treatment administration, dose modification, following AEs, or as clinically indicated.

Laboratory safety assessments are outlined in Table 10. In addition, for women of childbearing potential, a serum or urine pregnancy test will be performed by the local laboratory at screening. Pregnancy tests will also be done whenever pregnancy is suspected during the study. Additional pregnancy testing may be necessary if required by local practices or regulations.

**Table 10: Laboratory Safety Parameters**

| **Hematology Panel** | **Blood Chemistry Panel** |
|---|---|
| Hemoglobin | Aspartate aminotransferase (AST) |
| Platelet count | Alanine aminotransferase (ALT) |
| White blood cell count (WBC) | Alkaline phosphatase |
| Neutrophil count | Total bilirubin[1] |
| Lymphocyte count | Lipase |
| | Amylase |

### Table 10: Laboratory Safety Parameters (Continued)

| | |
|---|---|
| **Coagulation** | Sodium |
| International normalized ratio (INR) | Potassium |
| Partial thromboplastin time (PTT) | Chloride |
| | Bicarbonate or $CO_2$ |
| **Urinalysis (dip stick)** | Blood urea nitrogen (BUN) |
| Blood | Creatinine |
| Protein | Albumin |
| | Total Calcium |
| **Thyroid Function Test** | Magnesium |
| Thyroid-stimulating hormone (TSH) | Uric acid |

1 If total bilirubin is ≥2 × ULN and no evidence of Gilbert's syndrome, then fractionate into direct and indirect bilirubin.

### *7.2.4 Electrocardiograms*

Single and triplicate ECGs are to be performed as outlined in Table 1 and Table 3 (single) and Table 2 (triplicate). It is preferable that the machine used has a capacity to calculate the standard intervals automatically. Assessments reported by automated-read as prolongation of QTc should be over-read by a cardiologist to ensure accuracy of interpretation.

### *7.2.5 Echocardiogram or Multigated Acquisition Scan*

Echocardiogram (ECHO) (preferred) or multigated acquisition (MUGA) scan will be performed at screening, and thereafter as indicated in Table 1 and Table 3. Additional assessments of LVEF may be performed as clinically indicated at the Investigator's discretion if there are signs or symptoms of cardiotoxicity. The same imaging modality should be used for an individual patient throughout their participation in the study.

## 7.3 Laboratory Evaluations

Full details on sample collection, processing, storage, and shipment are presented in the Study Laboratory Manual.

### *7.3.1 Pharmacokinetic Evaluation*

The PK of sitravatinib will be determined using blood samples collected at specified time points prior to and following study treatment dosing as outlined in Table 2. Every effort should be made to collect these PK samples at the exact nominal times relative to dosing. A variation window is allowed for each time point. The actual time of each sample collection will be recorded on the source document and CRF.

All plasma samples will be stored frozen and shipped on dry ice according to instructions provided. Analysis of samples will be performed using specific validated bioanalytical

methods. Full details on sample collection, processing, storage and shipment will be provided in the Study Laboratory Manual.

### 7.3.2 Molecular Marker Evaluation

Molecular markers to be investigated include ctDNA assessment and optional tumor tissue assessments from fresh biopsies, which will be performed in a subset of patients at select sites as specified in Table 1 and Table 3. Tumor tissue assessments include PD-L1 expression, immune cell populations, gene expression signatures, and tumor gene alterations. Archival tumor tissues may also be analyzed, if available, for patients opting out of fresh tumor biopsies.

#### *7.3.2.1 Circulating Tumor DNA*

Blood samples for ctDNA analysis will be collected at baseline/screening as outlined in Table 1. Blood samples will be collected into two 10 mL Streck brand Cell-Free DNA Blood Collection tubes allowing shipping and stability at ambient temperatures. Tumor mutation analyses in ctDNA samples will be determined using next generation sequencing (NGS) performed by a central laboratory.

#### *7.3.2.2 Markers in Tumor Tissue*

Tumor tissue will be collected before study treatment, at the beginning of Cycle 2, and at the time of disease progression as specified in Table 1 and Table 3. Tumor tissue collection is optional, and only at select sites as specified in the Study Manual. The final time point is intended for those patients who have progressed after confirmed disease response (CR or PR).

Freshly biopsied tumor tissue is to be collected; however, tumor biopsies having significant risk should not be performed, and tumor lesions evaluated on-treatment as measurable lesions per RECIST 1.1 should not be disturbed for study biopsies if doing so may affect RECIST assessment, in the opinion of the Investigator. For patients who opt out of tumor tissue biopsy, archival tumor tissue will be collected, if available. For patients opting to undergo on-study biopsies, archival tissue will not be accepted as an alternative for the pre-treatment sample.

Samples should be of acceptable quality and quantity for analysis. Samples should be collected via a core needle of 18 gauge or larger with sufficient passes or be collected by an incisional or excisional tumor biopsy. Samples from fine needle aspirates (FNAs) are not appropriate for study evaluations. Further guidance on sample preparation, handling and submission can be found in the Study Laboratory Manual.

Tumor tissue assessments may include, but are not limited to, immunohistochemistry and/or other analytical methods for assessment of PD-L1 status and characterization of immune cell populations; tumor gene expression analysis; and nucleic acid sequencing using NGS analyses. For patients whose tumor tissue has previously been tested using

NGS, presence of specific tumor gene mutations and estimation of total mutation burden will be collected in the CRF.

## 7.4 Post-treatment Follow-up

Post-treatment follow-up consists of up to 3 follow-up visits and long-term follow-up. Refer to Table 3 for the timing of post-treatment activities.

Follow-up visit assessments may include ECOG, ECHO/MUGA scan, single 12-lead ECG, physical exam, vital signs, hematology, chemistry, thyroid function, and AEs as outlined in Table 3. In addition, tumor tissue may be biopsied in a subset of patients as described in Section 7.3.2.2 and Table 3.

During long-term follow-up, survival status and information concerning subsequent anticancer therapies will be collected as outlined in Table 3 until death or lost to follow-up. Follow-up may be performed by telephone or email. In the event a patient discontinues due to lost to follow-up or withdraws consent for use data from medical records, surveillance of public sources of death information (eg, published obituaries) should be undertaken and reported date of death recorded in the CRF.

# 8 ADVERSE EVENT REPORTING

## 8.1 Sponsor Medical Monitor Personnel

The contact information for the Sponsor's Medical Monitor personnel for this trial is available in the study contact list located in the Study Manual.

## 8.2 Adverse Events

An AE is any reaction, side effect or other undesirable medical event that occurs during participation in a clinical trial, regardless of treatment group or suspected causal relationship to study treatment. Assessment of AEs will include type, incidence, severity (graded by the National Cancer Institute [NCI] Common Terminology Criteria for Adverse Events [CTCAE] v5.0), timing, seriousness, and relatedness to study treatment.

All observed or volunteered AEs will be recorded in source documents and reported in the CRF. The best available medical terminology should be used to describe AEs in source documents and CRFs. Terms describing the diagnosis are preferred over individual signs and symptoms of the diagnosis. If determination of the diagnosis is delayed, record signs and symptoms and add the diagnosis as an additional AE when available; follow all recorded AEs to resolution. The actual date of onset should be recorded in all cases. Ongoing AEs that change in attribution or severity should have the date of change entered as the “end date” and a new AE record should be opened with the changed details. Examples of AEs include but are not limited to:

- Signs or symptoms of co-morbidity, illness, or toxicity of study treatment;
- Signs or symptoms of worsening malignancy under study (disease progression assessed by measurement of malignant lesions should not be reported as an AE).
- Laboratory abnormalities (see Section 8.2.1 for guidance for reporting in CRF);
- Hypersensitivity;
- Drug abuse, dependency, overdose, withdrawal or misuse;
- Signs or symptoms of drug interactions;
- Extravasation;
- Exposure during pregnancy or via breastfeeding;
- Medication error; or
- Occupational exposure.

### 8.2.1 Laboratory Abnormalities

An abnormal laboratory test result should be reported as an AE in the CRF only if it is associated with one or more of the following:

- Clinical symptoms;
- Requires additional tests (beyond repeats), treatment, or intervention;
- Results in change in study treatment dosing;
- Requires discontinuation from study treatment; and/or
- Considered by the Investigator or Sponsor to be an AE.

#### 8.2.1.1 Hy's Law

Hepatic function abnormality defined by an increase in AST and/or ALT to ≥ 3 × ULN concurrent with an increase in total bilirubin to ≥ 2 × ULN but without increase in alkaline phosphatase (i.e., ALP <2 × ULN) meets the criteria for Hy's Law and raises the concern for drug-induced liver injury when no other cause of the abnormal laboratory results is identified. Follow-up investigations and inquiries will be initiated promptly by the investigational site to determine whether the findings are reproducible and/or whether there is objective evidence that clearly supports causation by a disease (eg, cholelithiasis

and bile duct obstruction with distended gallbladder) or an agent other than the investigational product. Cases meeting Hy’s Law should be reported as SAEs..

### *8.2.2 Severity Assessment*

AEs occurring during this study will be graded in accordance with the NCI CTCAE v5.0. Documentation of AE grading in the source documents and CRF should be consistent with provided definitions.

### *8.2.3 Causality*

For each AE, the Investigator should determine and document whether there exists a reasonable possibility that any component of the study treatment caused or contributed to the AE. The Investigator’s assessment should be recorded in the source document. The CRF will provide the options for attribution to each study treatment as “related” or “not related.” If the Investigator's causality assessment is "unknown but not related to investigational product," this should be recorded in the CRF as “not related.” If the Investigator does not know whether or not the study treatment is causally related to the event, reporting for study purposes will be as “related” to study treatment.

Collection of causal relationship for AEs associated with study procedures (eg, tumor biopsy) is provided for separately in the CRF.

## 8.3 Serious Adverse Events

### *8.3.1 Definition of a Serious Adverse Event*

An SAE is any event that meets any of the following criteria:

- Results in death;
- Is life-threatening (immediate risk of death);
- Requires inpatient hospitalization or prolongation of existing hospitalization;
- Results in persistent or significant disability/permanent damage (substantial disruption of the ability to conduct normal life functions);
- Results in congenital anomaly/birth defect.
- Other: Important medical events that may not result in death, be life-threatening, or require hospitalization, may be considered an SAE when, based upon appropriate medical judgment, they may jeopardize the patient and may require medical or surgical intervention to prevent one of the outcomes listed in this definition. Examples of such events are:

   - Intensive treatment in an emergency room or at home for allergic bronchospasm;

   - Blood dyscrasias or convulsions that do not result in inpatient hospitalization;

   - Development of drug dependency or drug abuse.

- Progression of the malignancy under study, including any signs or symptoms of progression that may require hospitalization, should not be reported as an SAE unless the outcome is fatal within the safety reporting period.

**Definition of Terms**

Life-threatening: An AE is life threatening if the patient was at immediate risk of death from the event as it occurred; ie, it does not include a reaction that if it had occurred in a more serious form might have caused death. For example, drug-induced hepatitis that resolved without evidence of hepatic failure would not be considered life-threatening even though drug-induced hepatitis can be fatal.

Hospitalization: In general, hospitalization signifies that the patient has been detained (usually involving at least an overnight stay) at the hospital or emergency ward for observation and/or treatment that would not have been appropriate in the physician's office or outpatient setting. When in doubt as to whether 'hospitalization' occurred or was necessary, the AE should be considered serious. Hospitalization for elective surgery or routine clinical procedures that are not the result of AE (eg, elective surgery for a preexisting condition that has not worsened) need not be considered AEs or SAEs. If anything untoward is reported during the procedure, that occurrence must be reported as an AE, either 'serious' or 'non-serious' according to the usual criteria.

Disability/permanent damage: An AE is disabling or caused permanent damage if it resulted in a substantial disruption of a person's ability to conduct normal life functions, eg, a significant, persistent or permanent change, impairment, damage or disruption in body function/structure, physical activities and/or quality of life.

### *8.3.2 Exposure During Pregnancy*

Exposure during pregnancy (ie, exposure in-utero [EIU]) may occur in a female study participant, the female partner of a male study participant or study site personnel working with the investigational product (eg, occupational exposure) if:

- A female becomes or is found to be pregnant during treatment or within 6 months after discontinuing treatment or having been directly exposed to the investigational product.

- A male is exposed to the investigational product prior to or around the time of conception or during the pregnancy of his partner.

If EIU occurs, the Investigator must submit an SAE form and an EIU Supplemental Form within 24 hours of awareness of the exposure, regardless of whether an AE or SAE has occurred.

In the event of pregnancy in a female study participant, if the pregnancy is continued, study treatment will be immediately discontinued.

In the event of exposure of the pregnant partner of a male study participant, the study participant should be asked to deliver an EIU Pregnant Partner Release of Information Form to his partner. The Investigator must document on the EIU Form that the patient was given this letter to provide to his partner.

Follow-up to obtain pregnancy outcome information is to be conducted for all EIU reports. In the case of a live birth, the health of the neonate should be assessed at the time of birth and for up to 3 months after birth. Further follow-up of birth outcomes will be handled on a case-by-case basis (eg, follow-up on preterm infants to identify developmental delays). In the event the pregnancy is terminated, the reason(s) for termination should be reported and, if clinically possible, the structural integrity of the terminated fetus should be assessed by gross visual inspection.

If the outcome of the pregnancy meets the criteria for an SAE (ie, ectopic pregnancy, spontaneous abortion, intrauterine fetal demise, neonatal death, or congenital anomaly), an SAE report should be submitted to the Sponsor.

## 8.4 Reporting of SAEs and AEs

### *8.4.1 Reporting Period*

The reporting period for non-serious AEs begins from the day of the first dose of study treatment and continues until at least 28 days* after the last dose of study treatment. If a patient begins a subsequent anticancer therapy, the AE reporting period ends at the time the new treatment is started.

The active reporting period for SAEs begins from the time that the patient provides informed consent (ie, prior to undergoing any study-specific procedure or assessment) and continues until at least 28 days* after last dose of study treatment. All ongoing SAEs should be followed until they have resolved or stabilized to a chronic condition. If a patient begins a subsequent anticancer therapy, the reporting period for new SAEs ends at the time the new treatment is started.

> *The reporting period for all irAEs will continue until at least 100 days after the last dose of study treatment, or until the start of subsequent anticancer therapy, whichever occurs first.

Death must be reported if it occurs during the active reporting period for SAEs regardless of whether a subsequent anticancer therapy was administered. SAEs occurring after the

active reporting period has ended should be reported to the Sponsor if the Investigator becomes aware of them and if the Investigator assesses at least a reasonable possibility of being related to study treatment. These SAEs should be followed until resolved or stabilized to a chronic condition.

### *8.4.2 Reporting Requirements*

All SAEs must be reported within 24 hours of Investigator/site knowledge of the event, irrespective of the extent of available AE information, by faxing the SAE report to the Sponsor's pharmacovigilance representative designated in the Study Manual. The 24-hour timeframe also applies to additional new information (follow-up) on previously forwarded SAE reports and to the initial and follow-up reporting of exposure during pregnancy and exposure via breastfeeding. The need for an expedited report to regulatory authorities will be determined by the Sponsor and necessary reporting will be performed by the Sponsor. The Sponsor will notify study Investigators of all Suspected, Unexpected (as judged against the Investigator Brochure) Serious Adverse Reaction (SUSAR) reports. The Investigator is responsible for reporting all SUSARs to the IRB/EC.

All AEs (including SAEs) must be documented in source documents and reported in the CRF. Please note that the CRF and SAE report forms may collect information in somewhat different formats. Where the requested data overlap in different formats, the information should be consistent between the two forms.

# 9 STATISTICS

Detailed methodology for summary and statistical analyses of the data collected in this study will be documented in a Statistical Analysis Plan (SAP), which will be maintained by the Sponsor. The SAP may modify the plans outlined in the protocol; however, any major modifications of the primary endpoint definition and/or its analysis will also be reflected in a protocol amendment.

## 9.1 Hypotheses and Sample Size

### *9.1.1 Phase 1 Dose Escalation*

The time-to-event Bayesian optimal interval (TITE-BOIN) design (Yuan-2018) will be used to determine whether sequential dose escalation/de-escalation steps for sitravatinib in combination with nivolumab and ipilimumab described in Table 15 should be undertaken and to identify the maximum tolerated dose (MTD). The TITE-BOIN design is well-suited for use in dose escalation studies involving treatments associated with late-onset toxicity. The TITE-BOIN design allows dose escalation decisions for new patients while some patients continue evaluation for DLT at the previous dose level, thus shortening the overall duration of the trial. The model predicts the DLT outcome for ongoing patients based on their remaining follow-up time. Implementation is similar to

the traditional 3+3 Phase 1 design but is more flexible and possesses superior operating characteristics comparable to more complex model-based designs.

The TITE-BOIN model to be implemented in the current study is based on the following assumptions:

- The MTD is defined to have 0.3 probability of DLT;
- Initial cohort size is 3 patients;
- The overall duration of DLT assessment window is 9 weeks.

The approximate sample size in the Phase 1 segment of the study is 27 DLT-evaluable patients (defined in Section 9.3.5).

Table 11 shows the operating characteristics of the TITE-BOIN design (for dose finding) based on 1000 simulations of the trial. The time to toxicity is simulated from a uniform distribution Unif(0,T), and the patient accrual follows a Poisson process at the rate of 2 patients per month. The operating characteristics show that the design selects the true MTD, if any, with high probability and allocates more patients to the dose levels with the DLT rate closest to the target of 0.3.

**Table 11: Operating Characteristics of the TITE-BOIN Design**

| | Dose Level | | | | Number of Patients | % Early Stopping | Duration (months) |
|---|---|---|---|---|---|---|---|
| | -1 | 1 | 2 | 3 | | | |
| **Scenario 1** | | | | | | | |
| **True DLT Rate** | 0.30 | 0.47 | 0.55 | 0.64 | | | |
| **Selection %** | 58.2 | 28.1 | 3.3 | 0.0 | | 10.4 | 13.7 |
| **% Pts Treated** | 49.3 | 41.9 | 8.1 | 0.7 | 17.0 | | |
| **Scenario 2** | | | | | | | |
| **True DLT Rate** | 0.11 | 0.30 | 0.45 | 0.67 | | | |
| **Selection %** | 16.3 | 64.6 | 18.4 | 0.5 | | 0.2 | 14.0 |
| **% Pts Treated** | 23.8 | 52.6 | 20.7 | 2.9 | 18.5 | | |
| **Scenario 3** | | | | | | | |
| **True DLT Rate** | 0.02 | 0.13 | 0.30 | 0.47 | | | |
| **Selection %** | 0.5 | 30.3 | 55.9 | 13.3 | | 0.0 | 15.0 |
| **% Pts Treated** | 4.9 | 40.4 | 39.2 | 15.5 | 19.8 | | |
| **Scenario 4** | | | | | | | |
| **True DLT Rate** | 0.05 | 0.10 | 0.15 | 0.30 | | | |
| **Selection %** | 0.4 | 11.8 | 32.3 | 55.5 | | 0.0 | 16.1 |
| **% Pts Treated** | 3.4 | 27.8 | 34.0 | 34.7 | 20.6 | | |

Note: "% Early Stopping" refers to early stopping due to excessive toxicity.

Source: TITE BOIN app (v2.2.3.0) available at www.trialdesign.org.

Appendix 3 describes how dose escalation/de-escalation decisions will be reached during conduct of the Phase 1 portion of this study. Such decisions will be made by the Sponsor in collaboration with Investigators and will be communicated to sites via written correspondence prior to implementation. Decisions to remain at the current dose level may override decisions to dose escalate per TITE-BOIN design, based on clinical judgment of the Principal Investigator and in consultation with the Sponsor. In addition, to ensure sufficient safety information and early evidence of clinical activity are available at the dose regimen to be used in the Phase 1b dose expansion, enrollment at any dose regimen under consideration may be expanded to include at least 6 patients.

At the completion of the dose escalation portion of the study, the MTD will be identified based on isotonic regression as specified in Yuan-2018. This computation uses the BOIN Design Desktop Program (Venier-2017). Specifically, select as the MTD the dose for which the isotonic estimate of the toxicity rate is closest to the target toxicity rate. If there are ties, select the higher dose level when the isotonic estimate is lower than the target toxicity rate and select the lower dose level when the isotonic estimate is greater than or equal to the target toxicity rate.

Design and operating characteristics of the TITE-BOIN design are generated using TITE-BOIN app (v2.2.3.0) available at www.trialdesign.org.

### *9.1.2 Phase 1b Dose Expansion*

Time-to-event Bayesian Optimal Phase 2 (TOP) design will be used for Cohort A (first-line treatment in patients with intermediate and poor-risk ccRCC) and Cohort B (first-line treatment in patients with favorable-risk ccRCC) of the Phase 1b portion of study. The null hypothesis of 30% true response rate will be tested against alternative hypothesis of 50% response rate. The go/no-go decision at interim will be based on the totality of the data and the posterior probability of $Pr(p_{eff} > 0.3|data)$. If $Pr(p_{eff} > 0.3|data) < \lambda(\frac{n}{N})^{\alpha}$, then potentially stop the study at interim. Otherwise, continue the study. $\lambda$=0.84 and $\alpha$=1 are design parameters optimized to maximize the power under the alternative hypothesis; n and N are interim sample size and maximum sample size. A Beta (0.3,0.7) prior distribution is used to calculate posterior probability of $Pr(p_{eff} > 0.3|data)$.

Table 12 shows the operating characteristics of the TOP design (for cohort expansion) based on 1000 simulations of the trial. In the simulation study, the assessment window is 12 weeks, and the patient arrival is uniformly distributed with an accrual rate of 4 patients per month. The time to response is simulated from a Uniform distribution by controlling 50% of the responses occurring in the latter half of the response assessment window. The design and operating characteristics of the TOP design is generated using TOP design app (v1.0.2.0) available at www.trialdesign.org.

## Table 12: Operating Characteristics of the TOP Design

| Response rate | Early stopping (%) | Claim promising (%) | Sample size | Average trial duration (month) |
|---|---|---|---|---|
| 0.2 | 79.7 | 0.3 | 16.84 | 5.90 |
| 0.3 | 51.1 | 10.4 | 20.85 | 7.41 |
| 0.4 | 23.8 | 41.5 | 24.67 | 8.84 |
| 0.5 | 8.1 | 78.8 | 26.87 | 9.60 |

Source: TOP design app (v1.0.2.0) available at www.trialdesign.org.

Interim analysis will be conducted after 14 patients are enrolled and treated in each Phase 1b cohort. The go/no-go decision rule is outlined in Table 13.

## Table 13: Go/No-Go Rules for Phase 1b Dose Expansion Cohorts

| No. Patients Treated | No. Observed Responses | No. Pending Patients | Action |
|---|---|---|---|
| 14 | ≤ 3 | ≥ 7 | Suspend |
| 14 | ≤ 2 | ≤ 6 | No go |
| 14 | 3 | ≤ 6 | Go if ESS < 10.57 |
| 14 | ≥ 4 | ≤ 10 | Go |
| | | | |
| | | | |
| 28 | ≤ 28 | ≥ 1 | Suspend |
| 28 | ≤ 11 | 0 | No Go |
| 28 | ≥ 12 | 0 | Go |

ESS: effective sample size

To accelerate the trial, the TOP design makes early decisions by predicting the response of pending patents. It is possible that some pending patients have responses later, leading to the decision of “Go”. Thus, when the decision table indicates “No go”, the action of “Go” is still possible, depending on pending patients’ outcomes.

Suspend means suspending the accrual to wait for more response data available. ESS is the effective sample size, which is defined as

$$\text{ESS} = \text{Number of non} - \text{pending patients} + \frac{\text{sum of the follow} - \text{up time for pending patients}}{\text{length of assessment window}}$$

The length of assessment window is T=12 weeks.

When the total number of treated patients reaches the maximum sample size of 28, we reject the null hypothesis and conclude that the treatment is promising if the number of responses ≥12; otherwise we conclude that the treatment is not promising.

This study design yields a type I error rate at 0.1 level (one-sided) and 82.12% power when the true response rate is 0.5.

## 9.2 Data Handling

Listings of all patient data will be prepared. Data summaries will be presented in tabular and/or graphical format and summarized descriptively, where appropriate. Further details of planned analyses will be described in the SAP.

For all variables, only the observed data from patients will be used in the statistical analyses; there is no plan to estimate missing data. Patients without a valid clinical response assessment will be assigned a best overall response of not evaluable (NE). Data from patients who are lost to follow-up or have missing observations before reaching an endpoint in any of the time-to-event analyses will be treated as censored with specific rules defined in the SAP.

## 9.3 Analysis Populations

### *9.3.1 Enrolled Population*

The enrolled population is defined as all patients who sign an informed consent form for the study and are determined by the Investigator to meet all eligibility criteria during Screening assessments. This population will be used to describe disposition and may include patients who have not received study treatment.

### *9.3.2 Full Analysis Population*

The full analysis population (FAP) is defined as all patients who receive at least one dose of each study treatment drug (sitravatinib, nivolumab, and ipilimumab). FAP will be used for analysis of PFS and OS.

### *9.3.3 Clinical Activity Evaluable Population*

The Clinical Activity Evaluable population will include patients who receive at least one dose of each study treatment drug (sitravatinib, nivolumab, and ipilimumab) and have an evaluable baseline tumor assessment and at least one post-baseline tumor assessment.

This population will be used to present tumor responses data and summary statistics as well as to assist in enrollment decisions for the Phase 1b dose expansion portion of the study as described in Section 9.1.2.

### 9.3.4 Safety Population

The Safety population is defined as all patients who received at least one dose of any study treatment drug (ie, sitravatinib, nivolumab, or ipilimumab). The Safety population will be used for all safety analyses.

### 9.3.5 DLT-Evaluable Population

The DLT Evaluable population is defined as patients enrolled in the Phase 1 dose-escalation portion of the study who experienced a DLT or who cleared the DLT period (defined in Section 5.6). The DLT-evaluable population will be used to present the DLT AEs and for dose escalation decisions during Phase 1.

### 9.3.6 Pharmacokinetic Evaluable Population

The PK Evaluable population will consist of all patients who received treatment with sitravatinib and had sufficient concentration-time data to permit calculation of PK parameters for sitravatinib. For patients who were noncompliant with respect to administration of sitravatinib, or for patients with incomplete data, a decision as to their inclusion in the analysis will be made on a case-by-case basis.

### 9.3.7 Molecular Marker Evaluable Population

The molecular marker evaluable population will consist of all patients who receive at least one dose of any study treatment drug (ie, sitravatinib, nivolumab, or ipilimumab) for whom results are available.

## 9.4 Efficacy Endpoint Definitions and Analyses

### 9.4.1 Objective Response Rate

Objective disease response will be categorized in accordance with RECIST 1.1 (Appendix 5). Objective response rate (ORR) is defined as the percent of patients documented to have a confirmed CR or PR.

Descriptive statistics (frequency, percentage, and 90% CI) for ORR and best overall response (CR, PR, stable disease [SD], PD, NE) will be presented. The confidence interval of ORR will be calculated using Clopper-Pearson method. Additional details are described in the SAP.

### 9.4.2 Clinical Benefit Rate

CBR is defined as the percent of patients documented to have a best overall response of CR, PR, or SD during at least 1 on-study assessment and time on study of at least 6 weeks.

### *9.4.3 Duration of Response*

DOR is defined as the time from date of the first documentation of objective tumor response (CR or PR) to the first documentation of objective PD or to death due to any cause in the absence of documented PD. DOR will only be calculated for the subgroup of patients achieving a confirmed CR or PR. The Kaplan-Meier method will be used to estimate median DOR. Patients with objective tumor response, and alive without PD at the end of the study will be censored at the date of their last tumor assessment. Other rules and time points for censoring for DOR will be specified in SAP.

### *9.4.4 Progression-free Survival*

PFS is defined as the time from date of first study treatment to first PD or death due to any cause in the absence of documented PD. Censoring for the PFS endpoint will be assigned on the date of the last evaluable tumor assessment if no assessment of tumor progression is identified and the patient does not die while on study. Patients lacking an evaluation of disease after first study treatment will have their PFS time censored on the date of first dose with duration of 1 day. Patients who start a new anticancer therapy prior to documented PD will have the endpoint censored at the date of the last evaluable tumor assessment prior to the start of the new therapy. The Kaplan-Meier method will be used to obtain the estimate of median PFS.

### *9.4.5 Overall Survival*

Overall survival is defined as the time from date of first study treatment to death due to any cause. The Kaplan-Meier method will be used to estimate the median OS and 1-year Survival Rate; the 95% CI of the 1-year survival rate will also be reported. Censoring for the survival endpoint will be assigned on the date of the last on-study follow-up that the patient is reported to be alive.

### *9.4.6 Subgroup Analyses*

There are no planned subgroup analyses.

## 9.5 Safety Data Presentations and Summaries

### *9.5.1 Adverse Events*

For the purposes of this study, the term AE refers to treatment-emergent AEs (TEAEs), which are defined as AEs occurring from the date of the first dose until 28 days after the last dose of study treatment for non-irAEs, and until 100 days after the last dose of study treatment for irAEs.

AEs will be classified using the Medical Dictionary for Regulatory Activities (MedDRA) classification system. Listings will include the verbatim term, Preferred Term (PT), and System Organ Class (SOC). The number of patients with AEs and the incidence of AEs

by SOC and PT will be summarized. AEs will be summarized by maximum intensity and relationship to study therapy. Separate summaries will be provided for AEs, SAEs, treatment-related AEs, irAEs, treatment-related SAEs, and other significant AEs (eg, AEs leading to study discontinuation).

### 9.5.2 Prior and Concomitant Medications

Prior and concomitant medications will be coded using the World Health Organization (WHO) medical dictionary; patients who received these medications will be listed and summarized.

### 9.5.3 Clinical and Laboratory Assessments

Clinical and laboratory assessments include clinical laboratory tests (hematology, coagulation, urinalysis, thyroid function tests, and chemistry), vital signs, physical exams, ECOG performance status, ECHO/MUGA scan, and 12-lead ECGs.

Clinical laboratory results will be listed by patient and, as appropriate, summarized descriptively, which will include a display of change from baseline. Selected parameters will be presented in shift tables of baseline against worst grade test result. Laboratory values outside of the normal ranges will be identified. Laboratory values that meet Grade 3 or 4 criteria according to NCI CTCAE v5.0 will be listed and summarized.

ECG assessments will be evaluated for change of QTc from baseline as an exposure: response analysis. The Investigator's interpretation of QTc will be used in the clinical management of patients. The study analysis will use Fridericia's formula applied programmatically to the ECG data collected in CRFs using the QT interval and either the RR interval or the heart rate if the RR interval is not reported.

Vital signs, ECHO/MUGA results, and ECG measurements will be listed for each patient at each visit. Descriptive statistics of observed values and changes from baseline will be summarized by treatment group.

### 9.5.4 Patient Demographics, Baseline Characteristics and Disposition

Demographic, baseline disease characteristics, medical history, primary disease history and prior systemic therapies/surgeries will be summarized and listed based on safety population. Patient enrollment and disposition including reasons for study withdrawal and reasons for treatment discontinuation will be summarized.

### 9.5.5 Analysis of Study Treatment Dosing

Study treatment administration will be described in terms of the duration of exposure, total number of cycles administered, for each agent separately and for the combination, absolute and relative dose intensity, cumulative dose and reasons for the deviations from planned therapy.

## 9.6 Other Study Endpoints

### *9.6.1 Pharmacokinetic Analysis*

The PK sparse exposure data from this study may be used in the development of population PK and PK/PD models. PK plasma levels and parameters will be determined, listed, and summarized for the PK evaluable population in the PK Analysis Plan (PKAP). Only samples with acceptable PK (as defined in the PKAP) will be included in the summary statistics and a listing of individual data points or patients excluded from the analysis will be presented. Plasma concentrations will be listed by patient for the PK Population. Summary statistics of sitravatinib concentrations will be reported by dose level, Day, and Cycle. The exposure levels as well as the PK parameters of sitravatinib reported in earlier studies will be compared to the current study PK exposure and parameters to evaluate the potential effect of the study population, and concomitant administration of nivolumab on sitravatinib PK. Details of this analysis will be provided in the PKAP. Possible relationships between PK parameters, molecular marker variables, safety, and efficacy may be examined.

### *9.6.2 Molecular Marker and Exploratory Analyses*

No formal statistical analysis of molecular marker or other exploratory endpoints will be performed. Possible relationships between correlative endpoints, molecular marker variables, PK parameters, safety, and clinical activity may be examined if appropriate.

## 9.7 Interim Analysis

The go/no-go decision rule is outlined in Table 13.

## 9.8 Data Monitoring Committee

No Data Monitoring Committee is planned during this study.

# 10 ETHICS AND RESPONSIBILITIES

## 10.1 Ethical Conduct of the Study

This study will be conducted in accordance with International Ethical Guidelines for Biomedical Research Involving Human Patients (Council for International Organizations of Medical Sciences 2002), Guidelines for Good Clinical Practice (GCP) (International Council for Harmonisation [ICH] 1996), ICH E6 (R2) and concepts that have their origin in the Declaration of Helsinki (World Medical Association 1996, 2008, & 2013). Specifically, this study is based on adequately performed laboratory and animal experimentation; the study will be conducted under a protocol reviewed and approved by an IRB/EC; the study will be conducted by scientifically and medically qualified persons; the benefits of the study are in proportion to the risks; the rights and welfare of the

patients will be respected; the physicians conducting the study do not find the hazards to outweigh the potential benefits; and each patient will give his or her written informed consent before any protocol-driven tests or evaluations are performed.

## 10.2 Obligations of Investigators

The Investigator is responsible for complying with the protocol and all applicable regulations and guidelines governing clinical research. Additionally, he/she is responsible for ensuring that all participating staff members are adequately trained and competent to perform his/her assigned tasks.

All Investigators must provide the Sponsor with a current curriculum vitae. Only Investigators and designated Sub-Investigators are permitted to sign CRFs and examination findings (eg, laboratory results or ECGs).

The Investigator or designee is responsible for informing the patient of all available information relevant to his/her safety and obtaining signed, written consent from all participating patients. Additionally, the Investigator is responsible for monitoring patient safety and providing periodic and requested reports to the IRB/EC/Research Ethics Board (REB).

The Investigator is responsible for the accuracy and completeness of all study records including CRFs, source documents, and the Site Trial Master File. The Investigator will allow the Site Monitor, Sponsor, auditor, regulatory agencies, and IRB/EC/REB full access to the study and source documents.

## 10.3 Institutional Review Board/Ethics Committee/Research Ethics Board

Prior to the shipment of clinical supplies or initiation of the study, the clinical trial protocol along with the ICF, Investigator's Brochure, and any other written information or instructions for the patient must be submitted to the IRB/EC/REB for written approval. The Investigator will provide the Sponsor with a copy of the IRB/EC/REB's written approval, as well as the membership list or a compliance statement from the IRB/EC/REB. The Investigator is responsible for notifying the IRB/EC/REB of any Sponsor-approved amendments to the protocol or ICF, SAEs occurring in patients treated at the study site in accordance with local IRB/EC/REB practice, and all expedited safety reports from SAEs occurring at other study sites participating in the drug development program.

## 10.4 Informed Consent Form

The ICF must contain all elements required by the US FDA under 21 Code of Federal Regulations (CFR) Part 50 and the ICH GCP guidelines (ICH E6) in addition to any other elements required by applicable national, state, provincial, and local regulations, or institutional policies.

All patients who choose to participate in the study must provide written consent after having had adequate time to consider whether they will participate in the study. The written consent must be obtained prior to any protocol-related procedures that are not part of the patient's normal medical care. The patient must be advised of his/her right to withdraw from the study at any time.

Written documentation of consent must be recorded in the patient's source documents, study records, and CRF indicating the date the consent was signed. The patient should receive a signed copy of the consent form according to GCP guidelines.

### 10.5 Confidentiality

All information generated in this study is considered confidential, is subject to applicable privacy rules and regulations, and must not be disclosed to any person or entity not directly involved with the study unless prior written consent is gained from the Sponsor and otherwise except in accordance with applicable law or regulations. However, authorized regulatory officials, IRB/EC/REB personnel, the Sponsor and its authorized representatives (as and to the extent authorized in the patient's ICF) are allowed access to the records.

Identification of patients in CRFs shall be by study-assigned patient numbers only. If required, the patient's full name may be made known to an authorized regulatory agency or other authorized official.

### 10.6 Reporting of Serious Breaches of the Protocol or ICH GCP

In the event of any prohibition or restriction (ie, clinical hold) imposed by an applicable Regulatory Authority, or if the Investigator is aware of any new information which might influence the evaluation of the benefits and risks of the investigational product, the Sponsor must be informed immediately. In addition, the Investigator will inform the Sponsor immediately of any serious breaches of this protocol or of ICH GCP of which the Investigator becomes aware.

## 11 RECORDS MANAGEMENT

### 11.1 Source Documentation

Source documents include hospital or clinical patient charts, pertinent historical medical records, laboratory test reports, ECG tracings, pathology reports, radiographs, etc. All source documents must be legible. Data reported in CRFs and evidence of patient's informed consent must be documented in source documents.

### 11.2 Study Files and Records Retention

A CRF must be completed for each patient for whom informed consent for the study is obtained. The CRFs must be maintained by properly trained and delegated site representatives. The Principal Investigator has responsibility for ensuring the authenticity, accuracy, completeness and timeliness of all data collected in the CRF. CRFs must be signed by the Principal Investigator or by an authorized Sub-Investigator to attest that the information included is true.

Each study site will maintain a Site Trial Master File in accordance with GCPs.

The Investigator shall retain all records for the longest of the following periods:
(i) 15 years; (ii) the period of time that conforms to ICH GCP guidelines; (iii) the period of time required by applicable law or regulations; or (iv) the period of time specified in the Clinical Research Agreement.

## 12 QUALITY CONTROL AND QUALITY ASSURANCE

### 12.1 Monitoring Procedures

Sponsor appointed Site Monitor(s) must be allowed access to all study records, original source documents, and investigational products throughout the duration of the study. These personnel are responsible to assess compliance with the protocol, appropriate health authority regulations, ICH GCP guidelines, and Sponsor requirements.

The Site Monitor is responsible for complying with the monitoring guidelines established by the Sponsor for the study, assessing the site's needs, and liaising with the assigned Sponsor staff.

If the Investigator withdraws from the study and relinquishes his/her responsibility for the maintenance and retention of records, he/she must notify the Sponsor in writing so arrangements can be made to properly store the study materials.

### 12.2 Auditing and Inspection Procedures

The Sponsor's Quality Assurance representatives, IRB/EC/REB reviewers, or inspectors from regulatory agencies may perform an audit or inspection at any time during or after completion of the clinical study. All study-related documentation must be made available to the designated auditor. In addition, representatives of applicable regulatory health authorities may choose to inspect a study. A Sponsor representative will be available to assist in the preparation for such an inspection.

# 13 CHANGES IN STUDY CONDUCT

## 13.1 Protocol Amendments

Changes to the study protocol, except those intended to reduce immediate risk to study patients, may be made only by the Sponsor. A protocol change intended to eliminate an apparent immediate hazard to patients may be implemented immediately, provided the IRB/EC/REB is notified within 5 days. Any urgent safety measures taken by the Investigator to protect the study patients against any immediately life-threatening hazard must be reported immediately to the Sponsor.

Any permanent change to the protocol must be handled as a protocol amendment. The change will be documented in writing by the Sponsor, as an Administrative Letter or amended protocol. The written Administrative Letter or amendment must be submitted to the IRB/EC/REB by the Investigator, and the Investigator must await approval before implementing the changes. The Sponsor will be responsible for submitting protocol amendments to the appropriate regulatory authorities for approval.

If in the judgment of the IRB/EC/REB, the Investigator, and/or the Sponsor, the amendment to the protocol substantially changes the study design and/or increases the potential risk to the patient and/or has an impact on the patient's involvement as a study participant, the currently approved written ICF will require similar modification. In such cases, informed consents (revised as appropriate to address protocol amendments) will be obtained for patients enrolled in the study before continued participation.

## 13.2 Protocol Deviations

Prospective permission to deviate from the eligibility criteria for this protocol will not be provided by the Sponsor. Study-specified assessments should not be omitted and the study treatment regimen should not deviate from protocol specifications. Minor, occasional adjustments in the clinic visit schedule may be necessary for logistical reasons (eg, due to weather conditions) but must not become routine or systematically alter the study schedule. The IRB/EC/REB should be informed of any deviations that may affect a patient’s treatment or informed consent, especially those increasing potential risks, which must receive prior written approval by the IRB/EC/REB.

# 14 END OF TRIAL

## 14.1 End of Trial in a European Union Member State

End of Trial in a Member State of the European Union is defined as the time at which it is deemed that sufficient patients have been recruited and completed the study as stated in the regulatory application (ie, Clinical Trial Application [CTA]) and ethics application in the Member State.

### 14.2 End of Trial in all other Participating Countries

End of Trial in all other participating countries is defined as the time at which all patients enrolled in the study have completed the last study visit and data from those visits have been reviewed by the Investigator or designee.

### 14.3 Premature Termination

Premature termination of this study may occur at any time because of a regulatory authority decision, change in opinion of the IRB/EC/REB, drug safety concerns, or at the discretion of the Sponsor. In addition, the Sponsor retains the right to discontinue development of sitravatinib at any time. If termination becomes necessary, the Sponsor will inform the appropriate regulatory authorities of the termination and the reason. The Principal Investigator will inform the IRB/EC/REB of the same. In terminating the study, the Sponsor and the Principal Investigator will assure that adequate consideration is given to the protection of the patients' interests.

## 15 STUDY REPORT AND PUBLICATION POLICY

The Sponsor is responsible for preparing and providing the appropriate regulatory authorities with clinical study reports according to the applicable regulatory requirements.

The publication of study results will be governed by the applicable Clinical Research Agreement between the Sponsor and the Study Site and Investigator (as applicable).

# 16 REFERENCES

Alegre ML, Noel PJ, Eisfelder BJ, et al. Regulation of surface and intracellular expression of CTLA4 on mouse T cells. J Immunol. 1996;157(11):4762–4770.

American Cancer Society (ACS). Cancer Statistics Center. http://cancerstatisticscenter.cancer.org. (Accessed Feb 2020).

Blume-Jensen P, Hunter T. Oncogenic kinase signalling. Nature. 2001;411:355–365.

Cho YH, Kim MS, Chung HS, et al. Novel immunotherapy in metastatic renal cell carcinoma. Investig Clin Urol. 2017 Jul;58(4):220–227.

Choueiri TK, Escudier B, Powles T, et al. Cabozantinib versus Everolimus in Advanced Renal-Cell Carcinoma. N Engl J Med. 2015 Nov 5;373(19):1814–1823.

Cohen RB, Oudard S. Antiangiogenic therapy for advanced renal cell carcinoma: management of treatment-related toxicities. Invest New Drugs. 2012 Oct;30(5):2066–2079.

Eisenhauer EA, Therasse P, Bogaerts J, et al. New response evaluation criteria in solid tumours: revised RECIST guideline (version 1.1). Eur J Cancer. 2009;45(2):228–247.

Escudier B, Porta C, Schmidinger M, et al. Renal cell carcinoma: ESMO clinical practice guidelines for diagnosis, treatment and follow-up. Ann Oncol. 2014 Sep;25 Suppl 3:iii49–56.

Fischer S, Gillessen S, Rothermundt C. Sequence of treatment in locally advanced and metastatic renal cell carcinoma. Transl Androl Urol. 2015 Jun;4(3):310–325.

Garfield K, LaGrange CA. Cancer, renal cell. StatPearls [Internet]. Treasure Island (FL): StatPearls Publishing; 06 Jan 2018.

Hammers HJ, Plimack ER, Infante JR, et al. Safety and efficacy of nivolumab in combination with ipilimumab in metastatic renal cell carcinoma: The CheckMate 016 Study. J Clin Oncol. 2017;35(34):3851–3858.

Hellmann MD, Rizvi NA, Goldman JW, et al. Nivolumab plus ipilimumab as first-line treatment for advanced non-small-cell lung cancer (CheckMate 012): results of an open-label, phase 1, multicohort study. Lancet Oncol. 2017;18(1):31–41.

Heng D, Xie W, Regan M, et al. Prognostic factors for overall survival in patients with metastatic renal cell carcinoma treated with vascular endothelial growth factor-targeted agents: results from a large, multicenter study. J Clin Oncol 2009; 27(34):5794–5799.

Hodi FS, O'Day SJ, McDermott DF, et al. Improved survival with ipilimumab in patients with metastatic melanoma [published correction appears in N Engl J Med. 2010 Sep 23;363(13):1290]. N Engl J Med. 2010;363(8):711–723.

Johnson DB, Peng C, Sosman JA. Nivolumab in melanoma: latest evidence and clinical potential. Ther Adv Med Oncol 2015, Vol. 7(2) 97–106.

Karnofsky D, Burchenal J, The clinical evaluation of chemotherapeutic agents in cancer. In: MacLeod C, ed. Evaluation of Chemotherapeutic Agents. New York, NY: Columbia University Press; 1949:191–205.

Kurman JS, Murgu SD. Hyperprogressive disease in patients with non-small cell lung cancer on immunotherapy. J Thorac Dis. 2018;10(2):1124–1128.

Leach DR, Krummel MF, Allison JP. Enhancement of antitumor immunity by CTLA-4 blockade. Science. 1996;271(5256):1734–1736.

Mellman I, Coukos G, Dranoff G. Cancer immunotherapy comes of age. Nature. 2011;480(7378):480–489.

Motzer RJ, Escudier B, McDermott DF, et al. Nivolumab versus Everolimus in Advanced Renal-Cell Carcinoma. N Engl J Med. 2015a;373(19):1803–1813.

Motzer RJ, Escudier B, Tomczak P, et al. Axitinib versus sorafenib as second-line treatment for advanced renal cell carcinoma: overall survival analysis and updated results from a randomised phase 3 trial. Lancet Oncol. 2013;14(6):552–562. [published correction appears in Lancet Oncol. 2013 Jun;14(7):e254].

Motzer RJ, Hutson TE, Glen H, et al. Lenvatinib, everolimus, and the combination in patients with metastatic renal cell carcinoma: a randomised, phase 2, open-label, multicentre trial. Lancet Oncol. 2015b;16(15):1473–1482. [published correction appears in Lancet Oncol. 2016 Jul;17 (7):e270] [published correction appears in Lancet Oncol. 2018 Oct;19(10):e509].

Motzer RJ, Hutson TE, Ren M, et al. Independent assessment of lenvatinib plus everolimus in patients with metastatic renal cell carcinoma. Lancet Oncol. 2016;17(1):e4–e5.

Motzer RJ, Tannir NM, McDermott DF, et al. Nivolumab plus ipilimumab versus sunitinib in advanced renal-cell carcinoma. N Engl J Med. 2018;378(14):1277–1290.

Mu CY, Huang JA, Chen Y, et al. High expression of PD-L1 in lung cancer may contribute to poor prognosis and tumor cells immune escape through suppressing tumor infiltrating dendritic cells maturation. Med Oncol. 2011 Sep;28(3):682–688.

Muglia VF, Prando A. Renal cell carcinoma: histological classification and correlation with imaging findings. Radiol Bras. 2015 May-Jun;48(3):166–174.

National Comprehensive Cancer Network (NCCN) Clinical Practice Guidelines in Oncology (NCC Guidelines®). Kidney Cancer. v2.2020. Aug 2019:16.

Oken M, Creech R, Tormey D, et al. Toxicity and response criteria of the Eastern Cooperative Oncology Group. Am J Clin Oncol. 1982;5:649–655.

OPDIVO® (nivolumab) injection, for intravenous use [package insert]. Bristol-Myers Squibb Company, Princeton, NJ; Sep 2019.

Pardoll DM. The blockade of immune checkpoints in cancer immunotherapy. Nat Rev Cancer. 2012;12(4):252–264.

Postow MA, Harding J, Wolchok JD. Targeting immune checkpoints: releasing the restraints on anti-tumor immunity for patients with melanoma. Cancer J. 2012;18(2):153–159.

Rini BI, Escudier B, Tomczak P, et al. Comparative effectiveness of axitinib versus sorafenib in advanced renal cell carcinoma (AXIS): a randomised phase 3 trial [published correction appears in Lancet. 2012 Nov 24;380(9856):1818]. Lancet. 2011;378(9807):1931–1939.

Rini BI, McDermott DF, Hammers H, et al. Society for immunotherapy of cancer consensus statement on immunotherapy for the treatment of renal cell carcinoma. J Immunother Cancer. 2016 Nov 15;4:81.

Rini BI, Small EJ. Biology and clinical development of vascular endothelial growth factor-targeted therapy in renal cell carcinoma. J Clin Oncol. 2005 Feb 10;23(5):1028–1043.

Robert C, Long GV, Brady B, et al. Nivolumab in previously untreated melanoma without BRAF mutation. N Engl J Med. 2015;372(4):320–330.

Sevin A, Chen A, Atkinson B. Tyrosine kinase inhibitor induced pancreatitis. J Oncol Pharm Practice. 2012;19(3):257–260.

Sharma P, Siefker-Radtke A, de Braud F, et al. Nivolumab alone and with ipilimumab in previously treated metastatic urothelial carcinoma: CheckMate 032 Nivolumab 1 mg/kg plus ipilimumab 3 mg/kg expansion cohort results [published correction appears in J Clin Oncol. 2019 Aug 10;37(23):2094]. J Clin Oncol. 2019;37(19):1608–1616.

Tannir NM, Schwab G, Grünwald V. Cabozantinib: an active novel multikinase inhibitor in renal cell carcinoma. Curr Oncol Rep. 2017 Feb;19(2):14.

Topalian SL, Drake CG, Pardoll DM. Immune checkpoint blockade: a common denominator approach to cancer therapy. Cancer Cell. 2015;27(4):450-461.

Venier JJ, Norris C, Yuan Y. BOIN design desktop program, MD Anderson Cancer Center Software Download Kiosk, 2017. https://biostatistics.mdanderson.org/SoftwareDownload/SingleSoftware/Index/99 (Accessed Feb 2020).

Yan Q, Chen P, Lu A, Zhao P, Gu A. Association between CTLA-4 60G/A and -1661A/G polymorphisms and the risk of cancers: a meta-analysis. PLoS One. 2013;8(12):e83710.

Yau T, Kang Y, Kim TY, et al. Nivolumab (NIVO) + ipilimumab (IPI) combination therapy in patients (pts) with advanced hepatocellular carcinoma (aHCC): Results from CheckMate 040. J Clin Oncol. 2019;37(suppl; abstr 4012).

YERVOY® (ipilimumab) injection, for intravenous use [package insert]. Bristol-Myers Squibb Company, Princeton, NJ; May 2019.

Yuan Y, Lin R, Li D, et al. Time-to-event bayesian optimal interval design to accelerate phase I trials. Clin Cancer Res. 2018 Oct 15;24(20):4921-4930.

# APPENDIX 1. INTERNATIONAL METASTATIC RENAL CELL CARCINOMA DATABASE CONSORTIUM SCORING

To be eligible for an intermediate/poor-risk ccRCC cohort, at least one of the following prognostic factors must be present, as per the International Metastatic RCC Database Consortium (IMDC) criteria. If none of these factors are present, patients may be eligible for enrollment into the favorable-risk RCC cohort.

## IMDC Adverse Prognostic Factors

| **Parameter** | **Value** |
|---|---|
| KPS[1] | < 80% |
| Time since diagnosis | < 1 year from initial diagnosis to first dose of study treatment |
| Hemoglobin | < LLN |
| Corrected calcium[2] | > ULN |
| ANC | > ULN |
| Platelet count | > ULN |

Abbreviations: ANC = absolute neutrophil count; KPS = Karnofsky Performance Status; LLN = lower limit of normal; ULN = upper limit of normal.

1. Patients with KPS <70% are not eligible for the study based on the equivalent ECOG score of ≥2. See Appendix 2.
2. Corrected calcium (mg/dL) = measured total Ca (mg/dL) + 0.8 (4.0 - serum albumin [g/dL]), where 4.0 represents the average albumin level in g/dL.
   Corrected calcium (mmol/L) = measured total Ca (mmol/L) + 0.02 (40 - serum albumin [g/L]), where 40 represents the average albumin level in g/L.

## RCC Risk Group per IMDC Criteria

| **Number of Adverse Prognostic Factors Present** | **Risk Group** |
|---|---|
| 0 | Favorable |
| 1-2 | Intermediate |
| 3-6 | Poor |

Source: Heng-2009

# APPENDIX 2. PERFORMANCE STATUS

The ECOG Performance Status and the Karnofsky Performance Status are two widely used methods to assess the functional status of a patient. Table 14 outlines one commonly used comparison as presented by the ECOG-ARIN Cancer Research Group.

## Table 14: ECOG and Karnofsky Performance Status Comparison

| ECOG PERFORMANCE STATUS[1] | KARNOFSKY PERFORMANCE STATUS[2] |
|---|---|
| 0: Fully active, able to carry on all pre-disease performance without restriction | 100%: Normal, no complaints; no evidence of disease |
| | 90%: Able to carry on normal activity; minor signs or symptoms of disease |
| 1: Restricted in physically strenuous activity but ambulatory and able to carry out work of a light or sedentary nature, eg, light house work, office work | 80%: Normal activity with effort, some signs or symptoms of disease |
| | 70%: Cares for self but unable to carry on normal activity or to do active work |
| 2: Ambulatory and capable of all self-care but unable to carry out any work activities; up and about more than 50% of waking hours | 60%: Requires occasional assistance but is able to care for most of personal needs |
| | 50%: Requires considerable assistance and frequent medical care |
| 3: Capable of only limited self-care; confined to bed or chair more than 50% of waking hours | 40%: Disabled; requires special care and assistance |
| | 30%: Severely disabled; hospitalization is indicated although death not imminent |
| 4: Completely disabled; cannot carry on any self-care; totally confined to bed or chair | 20%: Very ill; hospitalization and active supportive care necessary |
| | 10%: Moribund |
| 5: Dead | 0%: Dead |

Derived from: https://ecog-acrin.org/resources/ecog-performance-status (accessed Mar 2021).
1. Source: Oken-1982
2. Source: Karnofsky-1949

# APPENDIX 3. IMPLEMENTATION OF TITE-BOIN DOSE ESCALATION/DE-ESCALATION MODEL

## Figure 1: Decision Schema

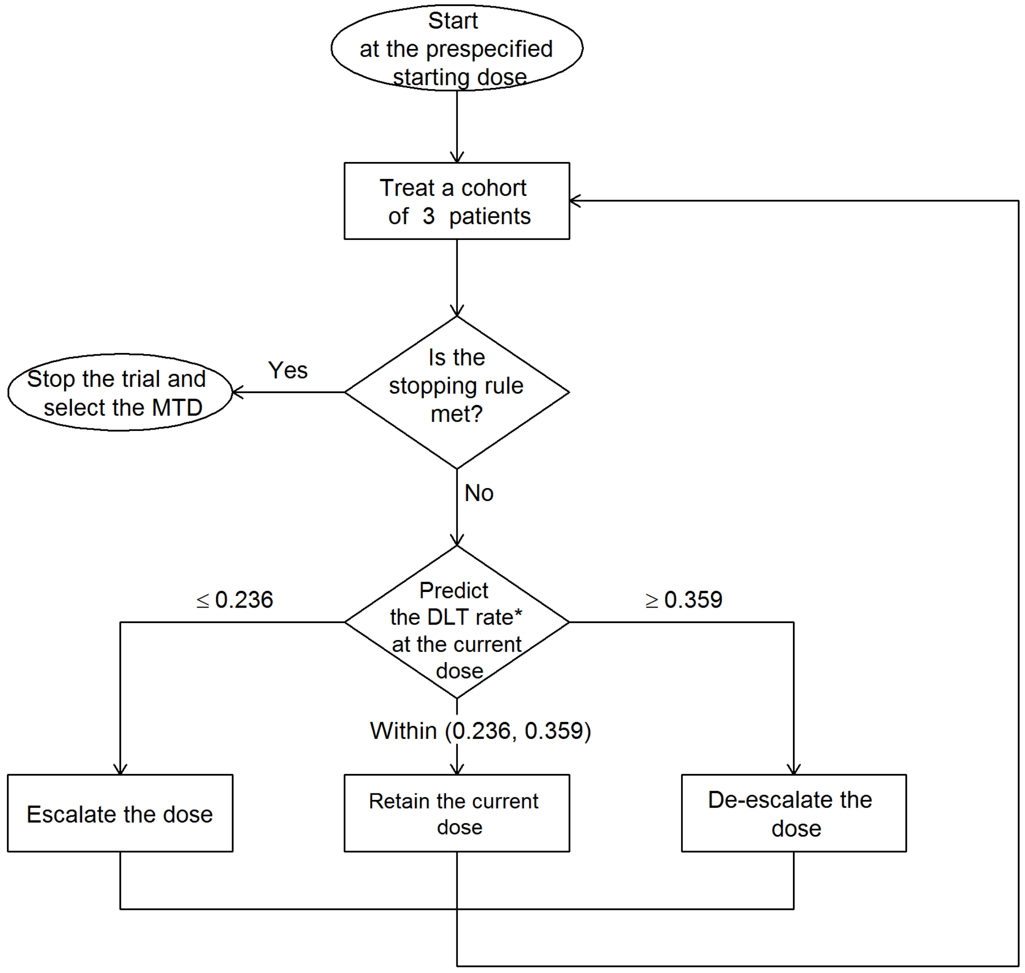


$$\text{* Predicted DLT rate} = \frac{\text{Predicted total number of patients who will experience DLT at the current dose}}{\text{Total number of patients treated at the current dose}}$$

Abbreviations: DLT = dose-limiting toxicity; MTD = maximum tolerated dose.

## Table 15: TITE-BOIN Design of Dose Escalation/ De-escalation Decision Table

| No. Treated | No. DLTs | No. Data Pending | STFT | | |
|---|---|---|---|---|---|
| | | | Escalate | Stay | De-escalate |
| 2 | 0 | ≤1 | Y | | |
| 2 | 0 | 2 | | Suspend accrual | |
| 2 | ≥1 | ≤1 | | | Y |
| 3 | 0 | ≤1 | Y | | |
| 3 | 0 | ≥2 | | Suspend accrual | |
| 3 | 1 | 0 | | Y | |
| 3 | 1 | 1 | | >0.88 | ≤0.88 |
| 3 | 1 | ≥2 | | Suspend accrual | |
| 3 | 2 | ≤1 | | | Y |
| 3 | 3 | 0 | | | Y&Elim |
| 4 | 0 | ≤2 | Y | | |
| 4 | 0 | ≥3 | Suspend accrual | | |
| 4 | 1 | ≤2 | | Y | |
| 4 | 1 | ≥3 | Suspend accrual | | |
| 4 | 2 | ≤2 | | | Y |
| 4 | ≥3 | ≤1 | | | Y&Elim |
| 5 | 0 | ≤2 | Y | | |
| 5 | 0 | ≤3 | | Suspend accrual | |
| 5 | 1 | 0 | Y | | |
| 5 | 1 | 1 | ≥0.39 | ≥0.39 | |
| 5 | 1 | 2 | ≥1.55 | ≥1.55 | |
| 5 | 1 | ≥3 | Suspend accrual | | |

| **No. Treated** | **No. DLTs** | **No. Data Pending** | **STFT** | | |
|---|---|---|---|---|---|
| | | | **Escalate** | **Stay** | **De-escalate** |
| 5 | 2,3 | ≥3 | | | Y |
| 5 | ≥4 | ≥1 | | | Y&Elim |
| 6 | 0 | ≤3 | Y | | |
| 6 | 0 | ≥4 | | Suspend accrual | |
| 6 | 1 | ≤1 | Y | | |
| 6 | 1 | 2 | ≥0.6 | <0.6 | |
| 6 | 1 | 3 | ≥1.96 | <1.96 | |
| 6 | 1 | ≥4 | | Suspend accrual | |
| 6 | 2 | 0 | | Y | |
| 6 | 2 | 1 | | >0.73 | ≤0.73 |
| 6 | 2 | 2 | | >1.8 | ≤1.8 |
| 6 | 2 | 3 | | >2.87 | ≤2.87 |
| 6 | 2 | ≥4 | | Suspend accrual | |
| 6 | 3 | ≤3 | | | Y |
| 6 | ≥4 | ≤2 | | | Y&Elim |
| 7 | 0 | ≤3 | Y | | |
| 7 | 0 | ≤4 | | Suspend accrual | |
| 7 | 1 | ≤2 | Y | | |
| 7 | 1 | 3 | ≥0.81 | ≥0.81 | |
| 7 | 1 | ≥4 | | Suspend accrual | |
| 7 | 2 | ≥3 | | Y | |
| 7 | 2 | ≥4 | | Suspend accrual | |
| 7 | 3,4 | ≥4 | | | Y |

| **No. Treated** | **No. DLTs** | **No. Data Pending** | **STFT** | | |
|---|---|---|---|---|---|
| | | | **Escalate** | **Stay** | **De-escalate** |
| 7 | ≥5 | ≥2 | | | Y&Elim |
| 8 | 0 | ≤4 | Y | | |
| 8 | 0 | ≥5 | Suspend accrual | | |
| 8 | 1 | ≤3 | Y | | |
| 8 | 1 | 4 | ≥1.01 | <1.01 | |
| 8 | 1 | ≥5 | Suspend accrual | | |
| 8 | 2 | ≤4 | | Y | |
| 8 | 2 | ≥5 | Suspend accrual | | |
| 8 | 3, 4 | ≤5 | | | Y |
| 8 | ≥5 | ≤3 | | | Y&Elim |
| 9 | 0 | ≤4 | Y | | |
| 9 | 0 | ≥5 | | Suspend accrual | |
| 9 | 1 | ≤4 | Y | | |
| 9 | 1 | ≥5 | | Suspend accrual | |
| 9 | 2 | 0 | Y | | |
| 9 | 2 | 1 | ≥0.59 | <0.59 | |
| 9 | 2 | 2 | ≥1.65 | <1.65 | |
| 9 | 2 | 3 | ≥2.71 | <2.71 | |
| 9 | 2 | 4 | ≥3.77 | <3.77 | |
| 9 | 2 | ≥5 | | Suspend accrual | |
| 9 | 3 | 0 | | Y | |
| 9 | 3 | 1 | | >0.58 | ≤0.58 |
| 9 | 3 | 2 | | >1.65 | ≤1.65 |

| No. Treated | No. DLTs | No. Data Pending | STFT | | |
|---|---|---|---|---|---|
| | | | Escalate | Stay | De-escalate |
| 9 | 3 | 3 | | >2.72 | ≤2.72 |
| 9 | 3 | 4 | | >3.79 | ≤3.79 |
| 9 | 3 | ≥5 | | Suspend accrual | |
| 9 | 4 | ≤5 | | | Y |
| 9 | ≥5 | ≤4 | | | Y&Elim |
| 10 | 0 | ≤5 | Y | | |
| 10 | 0 | ≥6 | Suspend accrual | | |
| 10 | 1 | ≤5 | Y | | |
| 10 | 1 | ≥6 | Suspend accrual | | |
| 10 | 2 | ≤1 | Y | | |
| 10 | 2 | 2 | ≥0.84 | <0.84 | |
| 10 | 2 | 3 | ≥2.01 | <2.01 | |
| 10 | 2 | 4 | ≥3.18 | <3.18 | |
| 10 | 2 | 5 | ≥4.35 | <4.35 | |
| 10 | 2 | ≥6 | Suspend accrual | | |
| 10 | 3 | ≤1 | | Y | |
| 10 | 3 | 2 | | >0.91 | ≤0.91 |
| 10 | 3 | 3 | | >2.1 | ≤2.1 |
| 10 | 3 | 4 | | >3.28 | ≤3.28 |
| 10 | 3 | 5 | | >4.47 | ≤4.47 |
| 10 | 3 | ≥6 | Suspend accrual | | |
| 10 | 4, 5 | ≤6 | | | Y |
| 10 | ≥6 | ≤4 | | | Y&Elim |

| No. Treated | No. DLTs | No. Data Pending | STFT | | |
|---|---|---|---|---|---|
| | | | Escalate | Stay | De-escalate |
| 11 | 0 | ≤5 | Y | | |
| 11 | 0 | ≥6 | | Suspend accrual | |
| 11 | 1 | ≤5 | Y | | |
| 11 | 1 | ≥6 | | Suspend accrual | |
| 11 | 2 | ≤2 | Y | | |
| 11 | 2 | 3 | ≥1.08 | <1.08 | |
| 11 | 2 | 4 | ≥2.36 | <2.36 | |
| 11 | 2 | 5 | ≥3.64 | <3.64 | |
| 11 | 2 | ≥6 | | Suspend accrual | |
| 11 | 4, 5 | ≤7 | | | Y |
| 11 | ≥6 | ≤5 | | | Y&Elim |
| 12 | 0 | ≤6 | Y | | |
| 12 | 0 | ≥7 | | Suspend accrual | |
| 12 | 1 | ≤6 | Y | | |
| 12 | 1 | ≥7 | | Suspend accrual | |
| 12 | 2 | ≤3 | Y | | |
| 12 | 2 | 4 | ≥1.33 | <1.33 | |
| 12 | 2 | 5 | ≥2.72 | <2.72 | |
| 12 | 2 | 6 | ≥4.11 | <4.11 | |
| 12 | 2 | ≥7 | | Suspend accrual | |
| 12 | 3 | ≤6 | | Y | |
| 12 | 3 | ≥7 | | Suspend accrual | |
| 12 | 4 | 0 | | Y | |

| No. Treated | No. DLTs | No. Data Pending | STFT | | |
|---|---|---|---|---|---|
| | | | **Escalate** | **Stay** | **De-escalate** |
| 12 | 4 | 1 | | >0.43 | ≤0.43 |
| 12 | 4 | 2 | | >1.5 | ≤1.5 |
| 12 | 4 | 3 | | >2.57 | ≤2.57 |
| 12 | 4 | 4 | | >3.65 | ≤3.65 |
| 12 | 4 | 5 | | >4.72 | ≤4.72 |
| 12 | 4 | 6 | | >5.79 | ≤5.79 |
| 12 | 4 | ≥7 | | Suspend accrual | |
| 12 | 5, 6 | ≤7 | | | Y |
| 12 | ≥7 | ≤5 | | | Y&Elim |

Note: "No. treated" is the total number of patients treated at the current dose level, "No. DLTs" is the number of patients who experienced DLT at the current dose level, "No. data pending" denotes the number of patients whose DLT data are pending at the current dose level, "STFT" is the standardized total follow-up time for the patients with data pending. "Y" represents "Yes", and "Y&Elim" represents "Yes and Eliminate".

Y&Elim – de-escalate and eliminate the current and higher doses.

If current dose is the lowest planned and table indicates de-escalate but not eliminate, treat new patients at that lowest dose level.

If the current dose level is the highest planned and table indicates escalate, treat new patients at that highest dose level.

If at the current dose level, more than 50% of the patients' DLT outcomes are pending, suspend accrual to wait for more data.

The STFT is defined as

$$\text{STFT} = \frac{\text{sum of the followup time for pending patients at the current dose}}{\text{length of the DLT assessment window}}$$

# APPENDIX 4. MEDICATIONS OR SUBSTANCES TO BE AVOIDED OR USED WITH CAUTION DURING TREATMENT WITH SITRAVATINIB

## Examples of Drugs with a Known Risk of Torsades de Pointes*

*Substantial evidence supports the conclusion that these drugs* ***prolong the QT interval*** *AND are* ***clearly associated with a risk of Torsades de Pointes****, even when taken as directed in official labeling.*

| Generic Name | Brand Names (Partial List) | Drug Class | Therapeutic Use |
|---|---|---|---|
| Aclarubicin (Only on Non US Market) | Aclacin, Aclacinomycine, Aclacinon, Aclaplastin, Jaclacin | Anti-cancer | Cancer |
| Amiodarone | Cordarone, Pacerone, Nexterone | Antiarrhythmic | Arrhythmia |
| Anagrelide | Agrylin, Xagrid | Phosphodiesterase 3 inhibitor | Thrombocythemia |
| Arsenic trioxide | Trisenox | Anti-cancer | Cancer (leukemia) |
| Astemizole (Removed from US Market) | Hismanal | Antihistamine | Allergic rhinitis |
| Azithromycin | Zithromax, Zmax | Antibiotic | Bacterial infection |
| Bepridil | Vascor | Antianginal | Angina Pectoris (heart pain) |
| Cesium Chloride | Energy Catalyst | Toxin | Alternative therapy cancer |
| Chloroquine | Aralen | Antimalarial | Malaria |
| Chlorpromazine | Thorazine, Largactil, Megaphen | Antipsychotic / Antiemetic | Nausea, Schizophrenia, many others |
| Chlorprothixene (Only on Non US Market) | Truxal | Antipsychotic | Schizophrenia |
| Cilostazol | Pletal | Phosphodiesterase 3 inhibitor | Intermittent claudication |
| Ciprofloxacin | Cipro, Cipro-XR, Neofloxin | Antibiotic | Bacterial infection |
| Cisapride (Removed from US Market) | Propulsid | GI stimulant | Increase GI motility |
| Citalopram | Celexa, Cipramil | Antidepressant, SSRI | Depression |
| Clarithromycin | Biaxin, Prevpac | Antibiotic | Bacterial infection |
| Cocaine | Cocaine | Local anesthetic | Anesthesia (topical) |
| Disopyramide | Norpace | Antiarrhythmic | Arrhythmia |
| Dofetilide | Tikosyn | Antiarrhythmic | Arrhythmia |

| Generic Name | Brand Names (Partial List) | Drug Class | Therapeutic Use |
|---|---|---|---|
| Domperidone (Only on Non US Market) | Motilium, Motillium, Motinorm Costi, Nomit | Antiemetic | Nausea, vomiting |
| Donepezil | Aricept | Cholinesterase inhibitor | Dementia (Alzheimer's Disease) |
| Dronedarone | Multaq | Antiarrhythmic | Arrhythmia |
| Droperidol | Inapsine, Droleptan, Dridol, Xomolix | Antipsychotic / Antiemetic | Anesthesia (adjunct), nausea |
| Erythromycin | E.E.S., Robimycin, EMycin, Erymax, Ery- Tab, Eryc Ranbaxy, Erypar, Eryped, Erythrocin Stearate Filmtab, Erythrocot, E-Base, Erythroped, Ilosone, MY-E, Pediamycin, Abboticin, Abboticin-ES, Erycin, PCE Dispertab, Stiemycine, Acnasol, Tiloryth | Antibiotic | Bacterial infection, increase GI motility |
| Escitalopram | Cipralex, Lexapro, Nexito, Anxiset-E, Exodus, Esto, Seroplex, Elicea, Lexamil, Lexam, Entact, Losita, Reposil, Animaxen, Esitalo, Lexamil | Antidepressant, SSRI | Depression (major), anxiety disorders |
| Flecainide | Tambocor, Almarytm, Apocard, Ecrinal, Flécaine | Antiarrhythmic | Arrhythmia |
| Fluconazole | Diflucan, Trican | Antifungal | Fungal infection |
| Gatifloxacin (Removed from US Market) | Tequin | Antibiotic | Bacterial infection |
| Grepafloxacin (Removed from US Market) | Raxar | Antibiotic | Bacterial infection |
| Halofantrine (Only on Non US Market) | Halfan | Antimalarial | Malaria |
| Haloperidol | Haldol, Aloperidin, Bioperidolo, Brotopon, Dozic, Duraperidol, Einalon S, Eukystol, Halosten, Keselan, Linton, Peluces, Serenace, Serenase, Sigaperidol | Antipsychotic | Schizophrenia, agitation |
| Hydroquinidine (Dihydroquinidine) (Only on Non US Market) | Serecor | Antiarrhythmic | Arrhythmia |
| Hydroxychloroquine | Plaquenil, Quineprox | Antimalarial, Anti-inflammatory | Malaria, SLE, rheumatoid arthritis |

| Generic Name | Brand Names (Partial List) | Drug Class | Therapeutic Use |
|---|---|---|---|
| Ibogaine (Only on Non US Market) | | Psychedelic | Narcotic addiction, unproven |
| Ibutilide | Corvert | Antiarrhythmic | Arrhythmia |
| Levofloxacin | Levaquin, Tavanic | Antibiotic | Bacterial infection |
| Levomepromazine (Methotrimeprazine) (Only on Non US Market) | Nosinan, Nozinan, Levoprome | Antipsychotic | Schizophrenia |
| Levomethadyl acetate (Removed from US Market) | Orlaam | Opioid agonist | Narcotic dependence |
| Levosulpiride (Only on Non US Market) | Lesuride, Levazeo, Enliva | Antipsychotic | Schizophrenia |
| Mesoridazine (Removed from US Market) | Serentil | Antipsychotic | Schizophrenia |
| Methadone | Dolophine, Symoron, Amidone, Methadose, Physeptone, Heptadon | Opioid agonist | Narcotic dependence, pain |
| Moxifloxacin | Avelox, Avalox, Avelon | Antibiotic | Bacterial infection |
| Nifekalant (Only on Non US Market) | Shinbit | Antiarrhythmic | Arrhythmia |
| Ondansetron | Zofran, Anset, Ondemet, Zuplenz, Emetron, Ondavell, Emeset, Ondisolv, Setronax | Antiemetic | Nausea, vomiting |
| Oxaliplatin | Eloxatin | Anti-cancer | Cancer |
| Papaverine HCl (Intra- coronary) | | Vasodilator, Coronary | Diagnostic adjunct |
| Pentamidine | Pentam | Antifungal | Fungal infection (Pneumocystis pneumonia) |
| Pimozide | Orap | Antipsychotic | Tourette's Disorder |
| Probucol (Removed from US Market) | Lorelco | Antilipemic | Hypercholesterolemia |
| Procainamide | Pronestyl, Procan | Antiarrhythmic | Arrhythmia |
| Propofol | Diprivan, Propoven | Anesthetic, general | Anesthesia |
| Quinidine | Quinaglute, Duraquin, Quinact, Quinidex, Cin-Quin, Quinora | Antiarrhythmic | Arrhythmia |
| Roxithromycin (Only on Non US Market) | Rulide, Xthrocin, Roxl-150, Roxo, Surlid, Rulide, Biaxsig, Roxar, Roximycinv, Roxomycin, Rulid, Tirabicin, Coroxin | Antibiotic | Bacterial infection |

| Generic Name | Brand Names (Partial List) | Drug Class | Therapeutic Use |
|---|---|---|---|
| Sevoflurane | Ultane, Sojourn | Anesthetic, general | Anesthesia |
| Sotalol | Betapace, Sotalex, Sotacor | Antiarrhythmic | Arrhythmia |
| Sparfloxacin (Removed from US Market) | Zagam | Antibiotic | Bacterial infection |
| Sulpiride (Only on Non US Market) | Dogmatil, Dolmatil, Eglonyl, Espiride, Modal, Sulpor | Antipsychotic, atypical | Schizophrenia |
| Sultopride (Only on Non US Market) | Barnetil, Barnotil, Topral | Antipsychotic, atypical | Schizophrenia |
| Terfenadine (Removed from US Market) | Seldane | Antihistamine | Allergic rhinitis |
| Terlipressin (Only on Non US Market) | Teripress, Glypressin, Terlipin, Remestyp, Tresil, Teriss | Vasoconstrictor | Septic shock |
| Terodiline (Only on Non US Market) | Micturin, Mictrol | Muscle relaxant | Bladder spasm |
| Thioridazine | Mellaril, Novoridazine, Thioril | Antipsychotic | Schizophrenia |
| Vandetanib | Caprelsa | Anti-cancer | Cancer (thyroid) |

* Woosley RL, Heise CW, Gallo T, Tate J, Woosley D and Romero KA, www.CredibleMeds.org, QTdrugs List, [Accession Date: 10 April 2020], AZCERT, Inc. 1822 Innovation Park Dr., Oro Valley, AZ 85755; for the most current information, access the website: www.CredibleMeds.org.

## Examples of Drugs with <u>Conditional</u> Risk of Torsades de Pointes*

*Substantial evidence supports the conclusion that these drugs are associated with a risk of Torsades de Pointes BUT only under certain conditions (eg, excessive dose, hypokalemia, congenital long QT or by causing a drug-drug interaction that results in excessive QT interval prolongation)*

| Generic Name | Brand Names (Partial List) | Drug Class | Therapeutic Use |
|---|---|---|---|
| Abiraterone | Zytiga, Abiratas, Abretone, Abirapro | Anti-androgen | Cancer (Prostate) |
| Amantadine | Symmetrel, Symadine | Antiviral | Viral infection (Influenza), Parkinson's disease |
| Amisulpride | Barhemsys, Solian, Supitac, Soltus, Amitrex, Amazeo | Antiemetic, Antipsychotic | Nausea and vomiting, postoperative |
| Amitriptyline | Elavil (Discontinued 6/13), Tryptomer, Tryptizol, Laroxyl, Saroten, Sarotex Lentizol, Endep | Antidepressant, Tricyclic | Depression |

| **Generic Name** | **Brand Names (Partial List)** | **Drug Class** | **Therapeutic Use** |
|---|---|---|---|
| Amphotericin B | Fungilin, Fungizone, Abelcet, AmBisome, Fungisome, Amphocil, Amphotec | Antifungal | Fungal infection |
| Amsacrine (Acridinyl anisidide)(Only on Non US Market) | Amsidine | Antineoplastic Agent | Cancer (Acute Lymphoblastic Leukemia) |
| Atazanavir | Reyataz, Evotaz | Antiviral | Viral infection (HIV/AIDS) |
| Bendroflumethiazide (Bendrofluazide) | Aprinox, Corzide | Diuretic, thiazide | Hypertension, diuresis |
| Chloral hydrate | Aquachloral, Novo-Chlorhydrate, Somnos, Noctec, Somnote | Sedative | Sedation, insomnia |
| Cimetidine | Tagamet | Antacid | Gastric hyperacidity, GERD |
| Clomipramine | Anafranil | Antidepressant, Tricyclic | Depression |
| Diphenhydramine | Benadryl, Nytol, Unisom, Sominex, Dimedrol, Daedalon, Banophen | Antihistamine | Allergic rhinitis, insomnia |
| Doxepin | Sinequan, Silenor, Aponal, Adapine, Doxal, Deptran, Sinquan | Antidepressant, Tricyclic | Depression |
| Eperisone (Only on Non US Market) | Myonal, Epry | Antispasmodic | Spasticity |
| Esomeprazole | Nexium, Nexum, Inexium | Proton Pump Inhibitor | Gastric hyperacidity, GERD |
| Famotidine | Pepcid, Fluxid, Quamatel | H2-receptor antagonist | Gastric hyperacidity, GERD |
| Fluoxetine | Prozac, Sarafem, Fontex | Antidepressant, SSRI | Depression |
| Fluvoxamine | Faverin, Fevarin, Floxyfral, Dumyrox, Luvox | Selective Serotonin Reuptake Inhibitor | Depression, Obsessive Compulsive Disorder |
| Furosemide (frusemide) | Lasix, Fusid, Frumex, Lasilix | Diuretic | Hypertension, diuresis |
| Galantamine | Reminyl, Nivalin, Razadyne-ER, Lycoremine | Cholinesterase inhibitor | Dementia (Alzheimer's Disease) |
| Garenoxacin (Only on Non US Market) | Geninax | Antibiotic | Bacterial infection |
| Hydrochlorothiazide | Apo-Hydro, Aquazide H, BP Zide, Dichlotride, Hydrodiuril, HydroSaluric, Microzide, Esidrex, Oretic | Diuretic | Hypertension, diuresis |

| **Generic Name** | **Brand Names (Partial List)** | **Drug Class** | **Therapeutic Use** |
|---|---|---|---|
| Hydroxyzine | Atarax, Vistaril, Aterax, Alamon, Durrax, Equipose, Masmoran, Orgatrax, Paxistil Quiess, Tran-Q, Tranquizine | Antihistamine | Allergic reaction, anxiety disorders |
| Indapamide | Lozol, Natrilix, Insig | Diuretic | Hypertension, diuresis |
| Itraconazole | Sporanox, Onmel | Antifungal | Fungal infection |
| Ivabradine | Procoralan, Coralan, Corlentor, Coraxan, Ivabid, Bradia | Antianginal | Angina Pectoris (heart pain) |
| Ketoconazole | Nizoral, Sebizole, Ketomed, Keton | Antifungal | Fungal infection |
| Lansoprazole | Prevacid, Ogast | Proton Pump Inhibitor | Gastric hyperacidity, GERD |
| Loperamide | Imodium | Opioid agonist | Diarrhea |
| Metoclopramide | Reglan, Afipran, Maxolon, Cerucal, Clopamon, Clopra, Maxeran, Maxolon, Metozolv, Plasil, Pramin, Primperan, Perinorm | Antiemetic | Nausea, vomiting |
| Metolazone | Zytanix, Zaroxolyn, Mykrox | Diuretic | Hypertension, diuresis |
| Metronidazole | Flagyl | Antibiotic | Trichomoniasis, amebiasis, bacterial infection |
| Nelfinavir | Viracept | Antiviral | Viral infection (HIV/AIDS) |
| Olanzapine | Zyprexa, Zydis, Relprevv | Antipsychotic, atypical | Schizophrenia, bipolar disorder |
| Omeprazole | Losec, Prilosec, Zegerid, Mopral | Proton Pump Inhibitor | Gastric hyperacidity, GERD |
| Pantoprazole | Protonix, Inipomp, Eupantol | Proton Pump Inhibitor | Gastric hyperacidity, GERD |
| Paroxetine | Paxil, Aropax, Pexeva, Seroxat, Sereupin, Seroxat, Deroxat | Antidepressant, SSRI | Depression |
| Piperacillin/Tazobactam | Tazosyn, Zosyn | Antibiotic | Bacterial infection |
| Posaconazole | Noxafil, Posamol | Antifungal | Fungal infection |
| Propafenone | Rythmol SR, Rytmonorm | Sodium channel blocker | Arrhythmia |
| Quetiapine | Seroquel | Antipsychotic, atypical | Schizophrenia |
| Quinine sulfate | Qualaquin, Hexaquine | Antimalarial | Malaria, leg cramps |
| Ranolazine | Ranexa, Ranozex | Antianginal | Angina Pectoris (heart pain) |

| Generic Name | Brand Names (Partial List) | Drug Class | Therapeutic Use |
|---|---|---|---|
| Risperidone | Risperdal | Antipsychotic, atypical | Schizophrenia |
| Sertraline | Zoloft, Lustral | Antidepressant, SSRI | Depression |
| Solifenacin | Vesicare | Muscle relaxant | Bladder spasm |
| Telaprevir | Incivo, Incivek | Antiviral | Viral infection (hepatitis C) |
| Torsemide (Torasemide) | Demadex, Diuver, Examide | Diuretic | Hypertension, diuresis |
| Trazodone | Desyrel, Oleptro, Beneficat, Deprax, Desirel, Molipaxin, Thombran, Trazorel, Trialodine, Trittico, Mesyrel | Antidepressant, SARI | Depression, insomnia |
| Voriconazole | VFend | Antifungal | Fungal infection |
| Ziprasidone | Geodon, Zeldox | Antipsychotic, atypical | Schizophrenia |

* Woosley RL, Heise CW , Gallo T, Tate J, Woosley D and Romero KA, www.CredibleMeds.org, QTdrugs List, [Accession Date: 10 April 2020], AZCERT, Inc. 1822 Innovation Park Dr., Oro Valley, AZ 85755; for the most current information, access the website: www.CredibleMeds.org.

## Examples of Sensitive Substrates and Substrates with Narrow Therapeutic Index for P-gp and BCRP transporters

| Enzyme | |
|---|---|
| P-gp | Aliskiren, ambrisentan, colchicine, dabigatran etexilate, digoxin, *everolimus*, fexofenadine, imatinib, lapatinib, maraviroc, nilotinib, posaconazole, ranolazine, saxagliptin, sirolimus, sitagliptin, talinolol, tolvaptan, topotecan |
| BCRP | Methotrexate, mitoxantrone, imatinib, irinotecan, lapatinib, rosuvastatin, sulfasalazine, topotecan |

## Examples of Sensitive Substrates and Substrates with Narrow Therapeutic Index for the indicated CYP3A4 Enzymes

| Enzyme | |
|---|---|
| CYP3A4 | Alfentanil, avanafil, budesonide, buspirone, conivaptan, darifenacin, darunavir, dasatinib dronedarone, ebastine, eletriptan, eplerenone, everolimus, felodipine, ibrutinib, indinavir, lomitapide, lovastatin, lurasidone, maraviroc, midazolam, naloxegol, nisoldipine, quetiapine, saquinavir, sildenafil, simvastatin, sirolimus, tacrolimus, ticagrelor, tolvaptan, triazolam, vardenafil |

# APPENDIX 5. ABBREVIATED PRESENTATION OF RECIST VERSION 1.1 GUIDELINES

***A modification to RECIST 1.1 has been made to account for the possibility of temporary changes resulting from tumor necrosis, cavitation, flare response or pseudoprogression.***

***Categorizing Lesions at Baseline***

***Measurable Lesions***

- Accurately measured in at least one dimension.
- When assessed by CT or MRI, longest diameter at least 10 mm or greater (slice thickness 5-8 mm), measured in the axial plane. If the slice thickness is greater than 5 mm (including any inter-slice gap), the longest diameter must be at least twice the slice thickness.
- Malignant lymph nodes with a short axis (defined as the largest measurement perpendicular to the longest diameter of the lesion) 15 mm or greater when assessed by CT or MRI.

The shortest axis is used as the diameter for malignant lymph nodes, longest axis for all other lesions.

***Non-Measurable Disease***

- Lesions too small to be considered measurable (including nodes with short axis between 10 and 14.9 mm) or truly non-measurable disease such as pleural or pericardial effusions, ascites, inflammatory breast disease, leptomeningeal disease, lymphangitic involvement of skin or lung, and abdominal masses identified by physical exam that are not measurable by reproducible imaging techniques.
- Bone disease is non-measurable with the exception of soft tissue components that can be evaluated by CT or MRI and meet the definition of measurability at baseline.
- Previously irradiated lesions (or those subjected to other local treatment) are non-measurable unless they have progressed since completion of treatment.

***Normal Lesions***

- Non-malignant simple cysts should not be recorded either as target or non-target disease. Cystic lesions thought to represent cystic metastases can be measurable lesions, if they meet the specific definition above.

- Lymph nodes with short axis <10 mm are considered normal and should not be followed as disease.

***Tumor Assessments***

All sites of disease must be assessed at baseline. Baseline assessments should be done as close as possible prior to study start. All required scans must be done within the window of time specified in the Schedule of Assessments prior to treatment. If the baseline assessment is inadequate, subsequent statuses generally should be indeterminate.

The determination of whether lesions are measurable is performed only at baseline. “Measurable” at baseline means eligible for selection as target lesions, and thus for quantitative assessment throughout the trial. Once selected as a target lesion, a lesion remains target throughout the trial.

***Target Lesions***

All measurable lesions up to a maximum of 2 lesions per organ, 5 lesions in total, representative of all involved organs, should be identified as target lesions at baseline. Target lesions should be selected on the basis of size (longest lesions) and suitability for accurate repeated measurements. Record the longest diameter for each lesion, except in the case of pathological lymph nodes for which the short axis should be recorded. The sum of the diameters (longest for non-nodal lesions, short axis for nodal lesions) for all target lesions at baseline will be the basis for comparison to look for PR at later assessments.

- If 2 target lesions coalesce the longest diameter measurement of the coalesced mass is used. If a large target lesion splits, the sum of the parts is used.

- Measurements for target lesions that become small should continue to be recorded. If a target lesion becomes too small to measure, 0 mm should be recorded if the lesion is considered to have disappeared; otherwise a default value of 5 mm should be recorded.

- When nodal lesions decrease to <10 mm (normal), the actual measurement should still be recorded.

***Non-Target Lesions***

All non-measurable disease is non-target. All measurable lesions not identified as target lesions are also included as non-target disease. Measurements are not required but rather qualitative evaluations of status will be recorded. Multiple non-target lesions in one organ may be recorded as a single item on the CRF (eg, ‘multiple liver metastases’).

***Objective Response Status at Each Evaluation***

Disease sites must be assessed using the same technique as baseline, including consistent administration of contrast. If not, subsequent objective statuses may be indeterminate.

***Target Disease***

- Complete Response (CR): Complete disappearance of all target lesions with the exception of nodal disease. All target nodes must decrease to normal size (short axis <10 mm). All target lesions must be assessed.
- Partial Response (PR): Greater than or equal to 30% decrease under baseline of the sum of diameters of all target measurable lesions. The short diameter is used in the sum for target nodes, while the longest diameter is used in the sum for all other target lesions. All target lesions must be assessed.
- Stable Disease (SD): Does not qualify for CR, PR or Progression. All target lesions must be assessed. Stable can follow PR only in the rare case that the sum increases by less than 20% from the nadir, but enough that a previously documented 30% decrease no longer holds.
- Progressive Disease (PD): 20% increase in the sum of diameters of target measurable lesions above the smallest sum observed (over baseline if no decrease in the sum is observed during therapy) with a minimum absolute increase of 5 mm.
- Indeterminate: Progression has not been documented, and
  - one or more target lesions have not been assessed,
  - or assessment methods used were inconsistent with those used at baseline and impaired assessment,
  - or one or more target lesions cannot be measured accurately (eg, poorly visible unless due to being too small to measure),
  - or one or more target lesions were excised or irradiated and have not reappeared or increased.

***Non-Target Disease***

- CR: Disappearance of all non-target lesions and normalization of tumor marker levels. All lymph nodes must be ‘normal’ in size (<10 mm short axis).
- Non-CR/Non-PD: Persistence of any non-target lesions and/or tumor marker level above the normal limits.

- PD: Unequivocal progression of preexisting lesions. Generally, the overall tumor burden must increase sufficiently to merit discontinuation of therapy. In the presence SD or PR in target disease, progression due to unequivocal increase in non-target disease should be rare.

- Indeterminate: Progression has not been determined and one or more non-target sites were not assessed or assessment methods were inconsistent with those used at baseline.

***New Lesions***

The appearance of any new unequivocal malignant lesion indicates PD. If a new lesion is equivocal, for example due to its small size, continued assessment will clarify the etiology. If repeat assessments confirm the lesion, then progression should be recorded on the date of the initial assessment. A lesion identified in an area not previously scanned will be considered a new lesion.

***Lesion Changes That May Be Transient***

Potential exists for individual tumor lesions to develop necrosis, cavitate, have a flare response to treatment, demonstrate pseudoprogression or become otherwise difficult to evaluate for a period of time as the result of study treatment impact. For example, tumor necrosis, cavitation, flare or pseudoprogression may result in increase in overall size of individual lesions, unclear tumor margins, or observation of apparently new lesions prior to recovery to smaller lesions, development of scar tissue, or complete resolution. The true tumor measurements of lesions should be recorded but the conclusion of PD may be suspended until continued assessment clarifies the nature of the tumor change. If repeat assessments indicate progression of disease, then PD should be recorded on the date of the first assessment giving the impression of progression. If repeat assessments indicate that the change was a process of transition, then NE should be recorded during the period of transition, and PR or CR may be recorded for subsequent evaluations. The CRF will collect information on the observations during the period of transition to support the assessment conclusions.

***Supplemental Investigations***

- If CR determination depends on a residual lesion that decreased in size but did not disappear completely, it is recommended the residual lesion be investigated with biopsy or fine needle aspirate. If no disease is identified, objective status is CR.

- 

- ***Best Objective Response***

| Target Lesions | Non-Target Lesions | New Lesion | Point in Time Response | Best Response |
|---|---|---|---|---|
| CR | CR | No | CR | CR and PR require confirmation at least 4 weeks after first observation |
| CR | Non-CR/Non-PD | No | PR | |
| PR | Non-PD | No | PR | |
| SD | Non-PD | No | SD | SD requires an on-study assessment after at least 6 weeks on treatment.<br>Unconfirmed PR or CR are reported as SD. |
| PD | Any | Yes or No | PD | |
| Any | PD | Yes or No | PD | |
| Any | Any | Yes | PD | |

***Subjective Progression***

Patients requiring discontinuation of treatment due to worsening health status attributable to advancement of the malignancy under study but without objective evidence of disease progression should not be reported as PD on tumor assessment CRFs. This should be indicated in the CRF as off treatment due to Global Deterioration of Health Status.

# APPENDIX 6. COVID-19 PANDEMIC CHANGES TO STUDY CONDUCT

The table below summarizes temporary changes to specific protocol assessments and procedures that are allowable during the COVID-19 pandemic (FDA-2021); these changes will not be recorded as protocol deviations unless otherwise specified. This appendix should be considered in effect until notification from Mirati is provided via Administrative Letter. Upon such notification, this appendix will no longer be applicable, and study conduct in accordance with the language in the body of the protocol will resume.

| **Assessment/Procedure** | **Change During COVID-19 Pandemic** |
|---|---|
| Visit Window | • Visit widows of up to ± 5 days are allowed for Day 1 (beyond Cycle 1) and Day 15 clinic visits. |
| Clinic Visits | • Clinic visits may be conducted remotely by telephone/video conference; any missed assessments or procedures (eg, tumor assessment, laboratory assessments, vital signs, study drug administration, etc.) should be documented as Protocol Deviations.<br>• Clinic visits may be conducted at the patient's residence by qualified home health care professionals; any missed assessments or procedures (eg, laboratory assessments, vital signs, study drug administration, etc.) should be documented as Protocol Deviations. |
| Safety Laboratory Assessments | Safety laboratory assessments may be performed at the patient's nearest CAP or CLIA certified local laboratory if a remote visit is being conducted, whenever feasible. |
| Nivolumab Dosing | In order to reduce the need for Day 15 visits, Investigators should:<br>• Consider switching all patients who are on the Q2 week 240 mg nivolumab regimen to the Q4 week 480 mg regimen instead, as possible.<br>• Start all newly enrolled patients on the Q4 week 480 mg regimen. |
| Day 15 Sample Collection for PK | In order to eliminate the Day 15 clinic visit:<br>• C1D15 PK samples, and triplicate ECG may be collected at C2D1 instead. |
| PK Sample Collections | The PK collection required post-dose at 7 hours (5-9 hours) on C1D1 and C1D15 may not be collected in order to reduce the time that patients need to remain in the clinic for the blood draw and ECGs. |
| Optional Tumor Tissue Collections | If biopsies are logistically problematic due to temporary institutional changes, the fresh tumor tissue collection at Screening/Baseline, on Cycle 2 Day 1, and at disease progression (clinical or objective; for patients who have a confirmed response) may be skipped, as these are optional. |

**Action and Reporting in the Event of Documented or Suspected COVID-19 Infection**

*Study Treatment*

- Patients in Screening for Study Entry – Patients who exhibit symptoms consistent with COVID-19 infection or have recent COVID-19 test results consistent with active viral replication should delay study entry and start of study treatment until resolution of symptoms and evaluation by the Investigator. Questions should be directed to the Sponsor's Medical Monitor.

- Patients in Study Treatment – For patients on study treatment exhibiting symptoms consistent with COVID-19 infection, contact the Sponsor's Medical Monitor as soon as feasible.
