# Supplementary material for: Sitravatinib in combination with nivolumab plus ipilimumab in patients with advanced clear cell renal cell carcinoma: a phase 1 trial
Source: Nat Commun. 2025 Jan 10;16:578. doi: 10.1038/s41467-024-55642-8 (PMC11724043; doi:10.1038/s41467-024-55642-8)
Supplement: Supplementary file 2 — Reporting Summary [file 41467_2024_55642_MOESM2_ESM.pdf]

nature portfolio

Corresponding author(s): Pavlos Msaouel, Linghua Wang, Jianjun Gao

Last updated by author(s): Nov 27, 2024

Reporting Summary

Nature Portfolio wishes to improve the reproducibility of the work that we publish. This form provides structure for consistency and transparency in reporting. For further information on Nature Portfolio policies, see our Editorial Policies and the Editorial Policy Checklist.

Statistics

For all statistical analyses, confirm that the following items are present in the figure legend, table legend, main text, or Methods section.

n/a	Confirmed	
☐	☒	The exact sample size (*n*) for each experimental group/condition, given as a discrete number and unit of measurement
☐	☒	A statement on whether measurements were taken from distinct samples or whether the same sample was measured repeatedly
☐	☒	The statistical test(s) used AND whether they are one- or two-sided<br>*Only common tests should be described solely by name; describe more complex techniques in the Methods section.*
☒	☐	A description of all covariates tested
☒	☐	A description of any assumptions or corrections, such as tests of normality and adjustment for multiple comparisons
☐	☒	A full description of the statistical parameters including central tendency (e.g. means) or other basic estimates (e.g. regression coefficient) AND variation (e.g. standard deviation) or associated estimates of uncertainty (e.g. confidence intervals)
☐	☒	For null hypothesis testing, the test statistic (e.g. *F*, *t*, *r*) with confidence intervals, effect sizes, degrees of freedom and *P* value noted<br>*Give P values as exact values whenever suitable.*
☐	☒	For Bayesian analysis, information on the choice of priors and Markov chain Monte Carlo settings
☒	☐	For hierarchical and complex designs, identification of the appropriate level for tests and full reporting of outcomes
☒	☐	Estimates of effect sizes (e.g. Cohen's *d*, Pearson's *r*), indicating how they were calculated

Our web collection on statistics for biologists contains articles on many of the points above.

Software and code

Policy information about availability of computer code

Data collection	Prometheus informatics system
Data analysis	R Version 4.3.2

For manuscripts utilizing custom algorithms or software that are central to the research but not yet described in published literature, software must be made available to editors and reviewers. We strongly encourage code deposition in a community repository (e.g. GitHub). See the Nature Portfolio guidelines for submitting code & software for further information.

Data

Policy information about availability of data

All manuscripts must include a data availability statement. This statement should provide the following information, where applicable:

- Accession codes, unique identifiers, or web links for publicly available datasets
- A description of any restrictions on data availability
- For clinical datasets or third party data, please ensure that the statement adheres to our policy

The trial protocol is available as a Supplementary File with this submission. Requests to access data should be forwarded to the corresponding authors at PMsaouel@mdanderson.org and/or jgao1@mdanderson.org and/or lwang22@mdanderson.org. All sequencing data are available at https://www.ncbi.nlm.nih.gov/geo/query/acc.cgi?acc=GSE264586 (GEO accession number GSE264586). Source data are provided with this paper.

Research involving human participants, their data, or biological material

Policy information about studies with human participants or human data. See also policy information about sex, gender (identity/presentation), and sexual orientation and race, ethnicity and racism.

Reporting on sex and gender	Findings apply to both sexes. Sex was not considered in study design; both males and females were eligible for study. Sex was determined based on self-reporting.<br>For the enrolled population on trial protocol (N=22)<br>Sex, n (%)<br>Male, 17 (77.3)<br>Female, 5 (22.7)<br>See manuscript for details.
Reporting on race, ethnicity, or other socially relevant groupings	Findings apply to all ethnicities. Ethnicity was not considered in study design; all ethnicities were eligible for study. Ethnicity was determined based on self-reporting.<br>For the enrolled population on trial protocol (N=22)<br>Ethnicity, n (%)<br>White, 17 (77.3)<br>Black, 2 (9.1)<br>East Asian, 2 (9.1)<br>South Asian, 1 (4.5)<br>See manuscript for details.
Population characteristics	Eligible patients were ≥ 18 years old, had pathologic confirmation of advanced or metastatic, poor- or intermediate-risk renal cell carcinoma (RCC) with a clear cell component. Eligible patients had no prior treatment with systemic therapy with the following exception: One prior adjuvant or neoadjuvant therapy for completely resectable RCC was allowed if such therapy did not include an agent that targets VEGF or VEGF receptors or any other antibody or drug targeting T-cell co-stimulation or checkpoint pathways (including, but not limited to: an anti-PD-1, anti-PD-L1, anti-PDL2, anti-CD137, or anti-CTLA-4 antibody), and if recurrence occurred at least 6 months after the last dose of adjuvant or neoadjuvant therapy. Additional key inclusion criteria were measurable disease according to Response Evaluation Criteria in Solid Tumors (RECIST version 1.1) and an Eastern Cooperative Oncology Group performance status score of 0 or 1. Patients were excluded if they had brain metastases, conditions requiring corticosteroids (>10 mg daily of prednisone or equivalent) or other immunosuppressive medication, autoimmune disease, or history of deep vein thrombosis or pulmonary embolism within the past 6 months (unless stable and treated with appropriate anticoagulation with the exception of warfarin).
Recruitment	Patients were recruited on this clinical trial (NCT04518046) by medical oncologists who treat clear cell renal cell carcinoma at MD Anderson. The total number of patients enrolled was 22. All patients provided written informed consent to participate based on the principles of the Declaration of Helsinki.
Ethics oversight	This clinical trial (NCT04518046) was approved by the Institutional Review Board (IRB) of MD Anderson (protocol 2020-0264). Patient samples for correlative studies were approved by the same IRB and MD Anderson protocol.

Note that full information on the approval of the study protocol must also be provided in the manuscript.

Field-specific reporting

Please select the one below that is the best fit for your research. If you are not sure, read the appropriate sections before making your selection.

☒ Life sciences ☐ Behavioural & social sciences ☐ Ecological, evolutionary & environmental sciences

For a reference copy of the document with all sections, see nature.com/documents/nr-reporting-summary-flat.pdf

Life sciences study design

All studies must disclose on these points even when the disclosure is negative.

Sample size	The time-to-event Bayesian optimal interval (TITE-BOIN) design was used to determine whether sequential dose escalation/de-escalation steps for sitravatinib in combination with nivolumab and ipilimumab described in Appendix 3 of the trial protocol (available in the supplementary note) should be undertaken and to identify the maximum tolerated dose (MTD). The TITE-BOIN design is well suited for use in dose escalation studies involving treatments associated with late onset toxicity, such as the triplet immunotherapy regimen evaluated in the present study, because it allows dose escalation decisions for new patients while some patients continue evaluation for dose-limiting toxicity (DLT) at the previous dose level, thus shortening the overall duration of the trial. The model predicts the DLT outcome for ongoing patients based on their remaining follow-up time. Implementation is similar to the traditional 3+3 Phase 1 design but is more flexible and possesses superior operating characteristics comparable to more complex model-based designs.<br><br>The TITE-BOIN model implemented in this study was based on the following assumptions:<br>•The MTD is defined to have 0.3 probability of DLT;<br>•Initial cohort size is 3 patients;<br>• The overall duration of DLT assessment window is 9 weeks.

	The approximate sample size was 27 DLT evaluable patients, defined as patients enrolled in the Phase 1 dose escalation portion of the study who experienced a DLT or who cleared the DLT period. Further information on the operating characteristics of the TITE-BOIN design for dose finding based on 1000 simulations of the trial, the decision schema and the decision table for escalation/de-escalation is included in the trial protocol available in the supplementary note.
Data exclusions	All enrolled patients who started treatment on trial were included in the analysis. Correlative data was included based on tissue/blood sample availability and passing quality control metrics.
Replication	For the clinical study, no efforts to verify reproducibility were attempted. See sample size section for rationale on target patient enrollment. For correlative studies, efforts were made to identify trends across patients in the study. All preclinical experiments were performed with at least two or more replicates.
Randomization	Allocation was not random as this was a single arm study.
Blinding	Blinding was not applicable to the clinical trial as it was a single arm study. All preclinical studies were planned and performed to ensure that each experiment contains all groups and appropriate controls. Investigators were not blinded to group allocation during data collection and/or analysis.

Reporting for specific materials, systems and methods

We require information from authors about some types of materials, experimental systems and methods used in many studies. Here, indicate whether each material, system or method listed is relevant to your study. If you are not sure if a list item applies to your research, read the appropriate section before selecting a response.

Materials & experimental systems

n/a	Involved in the study
☒	☐ Antibodies
☒	☐ Eukaryotic cell lines
☒	☐ Palaeontology and archaeology
☒	☐ Animals and other organisms
☐	☒ Clinical data
☒	☐ Dual use research of concern
☒	☐ Plants

Methods

n/a	Involved in the study
☒	☐ ChIP-seq
☒	☐ Flow cytometry
☒	☐ MRI-based neuroimaging

Clinical data

Policy information about clinical studies

All manuscripts should comply with the ICMJE guidelines for publication of clinical research and a completed CONSORT checklist must be included with all submissions.

Clinical trial registration	NCT04518046
Study protocol	Available with the manuscript as a Supplementary Note
Data collection	A total of 22 patients with treatment-naive clear cell renal cell carcinoma were enrolled from September 2020 to July 2022
Outcomes	The primary study objective was to evaluate safety of the triplet combination regimen, as characterized by type, incidence, severity, timing, seriousness, and relationship to study treatment of AEs, and laboratory abnormalities. Secondary objectives and endpoints were clinical activity, as characterized by objective response rate (ORR), duration of response (DOR), disease control rate (DCR) termed in the trial protocol as clinical benefit rate (CBR) and defined as the percent of patients documented to have a best overall response of CR, PR, or SD; progression-free survival (PFS), one-year survival probability, and overall survival (OS); and pharmacokinetics (PK) of sitravatinib when administered in the triplet combination. Exploratory endpoints included gene expression signatures and immune cell populations in the tumour.

Plants

Seed stocks	N/A
Novel plant genotypes	N/A
Authentication	N/A
