# Supplementary material for: Sitravatinib in combination with nivolumab plus ipilimumab in patients with advanced clear cell renal cell carcinoma: a phase 1 trial
Source: Nat Commun. 2025 Jan 10;16:578. doi: 10.1038/s41467-024-55642-8 (PMC11724043; doi:10.1038/s41467-024-55642-8)
Supplement: Supplementary file 3 — Description of Additional Supplementary Files [file 41467_2024_55642_MOESM3_ESM.pdf]

- **Supplementary Data file 1.** Treatment-related adverse events in patients enrolled in cohort 1 (n = 7).
- **Supplementary Data file 2.** Treatment-related adverse events in patients enrolled in cohort 2 (n = 3).
- **Supplementary Data file 3.** Treatment-related adverse events in patients enrolled in cohort 3 (n = 3).
- **Supplementary Data file 4.** Treatment-related adverse events in patients enrolled in cohort 4 (n = 9).
- **Supplementary Data file 5.** Treatment-related adverse events in patients enrolled in all cohorts (n = 22).
- **Supplementary Data file 6.** Sitravatinib-related adverse events in patients enrolled in all cohorts (n = 22).
- **Supplementary Data file 7.** Nivolumab/ipilimumab-related adverse events in patients enrolled in all cohorts (n = 22).
- **Supplementary Data file 8.** Sitravatinib pharmacokinetic (PK) concentrations by cohort and timepoint. Concentrations are in units of ng/ml.
- **Supplementary Data file 9.** Top 50 expressed markers for each epithelial cell cluster. Differentially expressed markers between cell types were identified using Seurat's FindAllMarkers function with the default two-sided wilcoxon rank-sum test.
